# Dependency Resolution Difficulty Increases with Distance in Persian Separable Complex Predicates: Evidence for Expectation and Memory-Based Accounts

**DOI:** 10.3389/fpsyg.2016.00403

**Published:** 2016-03-30

**Authors:** Molood S. Safavi, Samar Husain, Shravan Vasishth

**Affiliations:** ^1^International Doctorate for Experimental Approaches to Language and Brain (IDEALAB), University of Potsdam, Germany / University of Groningen, Netherlands / University of Trento, Italy / University of Newcastle, UK / Macquarie University SydneyAustralia; ^2^Department of Humanities and Social Sciences, Indian Institute of TechnologyNew Delhi, India; ^3^Department of Linguistics, University of PotsdamPotsdam, Germany

**Keywords:** locality, expectation, surprisal, entropy, Persian, complex predicates, self-paced reading, eye-tracking

## Abstract

Delaying the appearance of a verb in a noun-verb dependency tends to increase processing difficulty at the verb; one explanation for this locality effect is decay and/or interference of the noun in working memory. Surprisal, an expectation-based account, predicts that delaying the appearance of a verb either renders it no more predictable or more predictable, leading respectively to a prediction of no effect of distance or a facilitation. Recently, Husain et al. (2014) suggested that when the exact identity of the upcoming verb is predictable (strong predictability), increasing argument-verb distance leads to facilitation effects, which is consistent with surprisal; but when the exact identity of the upcoming verb is not predictable (weak predictability), locality effects are seen. We investigated Husain et al.'s proposal using Persian complex predicates (CPs), which consist of a non-verbal element—a noun in the current study—and a verb. In CPs, once the noun has been read, the exact identity of the verb is highly predictable (strong predictability); this was confirmed using a sentence completion study. In two self-paced reading (SPR) and two eye-tracking (ET) experiments, we delayed the appearance of the verb by interposing a relative clause (Experiments 1 and 3) or a long PP (Experiments 2 and 4). We also included a simple Noun-Verb predicate configuration with the same distance manipulation; here, the exact identity of the verb was not predictable (weak predictability). Thus, the design crossed Predictability Strength and Distance. We found that, consistent with surprisal, the verb in the strong predictability conditions was read faster than in the weak predictability conditions. Furthermore, greater verb-argument distance led to slower reading times; strong predictability did not neutralize or attenuate the locality effects. As regards the effect of distance on dependency resolution difficulty, these four experiments present evidence in favor of working memory accounts of argument-verb dependency resolution, and against the surprisal-based expectation account of Levy (2008). However, another expectation-based measure, entropy, which was computed using the offline sentence completion data, predicts reading times in Experiment 1 but not in the other experiments. Because participants tend to produce more ungrammatical continuations in the long-distance condition in Experiment 1, we suggest that forgetting due to memory overload leads to greater entropy at the verb.

## 1. Introduction

A long-standing claim in sentence processing is that increasing distance in a linguistic dependency, such as a noun-verb dependency, leads to greater processing difficulty (Chomsky, 1965; Just and Carpenter, 1992; Gibson, 2000; Lewis and Vasishth, 2005); it is common to refer to this increase in processing difficulty as the locality effect. One explanation for the locality effect is in terms of constraints imposed by working memory. According to one account, the Dependency Locality Theory (DLT; Gibson, 1998), the processing difficulty experienced when resolving a long dependency depends on the decay experienced by the noun; a related account by Lewis and Vasishth (2005) attributes the locality effect to decay and/or interference. Constraints on working memory may be a plausible explanation given that individuals' working memory capacity seems to affect the processes involved in dependency resolution (Caplan and Waters, 2013; Nicenboim et al., 2015). Although there is evidence consistent with the memory-based explanation in English, German, Chinese, Russian, and Hindi, (Hsiao and Gibson, 2003; Grodner and Gibson, 2005; Bartek et al., 2011; Vasishth and Drenhaus, 2011; Levy et al., 2013; Husain et al., 2014, 2015), research on some of these languages has also uncovered evidence that increasing noun-verb distance facilitates processing at the verb (Konieczny, 2000; Vasishth, 2003; Vasishth and Lewis, 2006; Jaeger et al., 2008; Vasishth and Drenhaus, 2011; Levy and Keller, 2013; Husain et al., 2014; Jäger et al., 2015). One explanation for these anti-locality effects is in terms of surprisal (Hale, 2001; Levy, 2008). Surprisal extends and formalizes the old idea of predictive sentence processing—which has been extensively investigated in the EEG literature (e.g., Kutas and Hillyard, 1984)—in terms of probabilistic parse continuations (also see Jurafsky, 1996). The surprisal account assumes that the comprehender maintains and uses linguistic knowledge probabilistically to parse a sentence incrementally. Surprisal is the claim that rare transitions are difficult: increased processing difficulty is predicted when a parser is required to build a low-probability syntactic structure. Formally, surprisal is defined as the negative log probability of encountering a particular part of speech or word given previous context. We will refer to surprisal as the expectation-based account, following the terminology of Levy (2008)^1^.

In many of these studies, evidence has been found for both the memory-based accounts and the expectation-based account. One conclusion that has emerged is that both memory and expectation play a role. For example, in his eye-tracking (ET) study investigating processing difference in English object vs. subject relative clauses, Staub (2010) finds evidence for both expectation-based processing and locality constraints, although these occur in different regions of the target sentence. An example of Staub's design is provided below. In this study, processing difficulty was found on the noun phrase *the fireman* in the ORC (object relative clause) 1b, compared to the SRC (subject relative clause) 1a; this is consistent with the expectation account because the reader would be forced to build a rare object relative in the ORC condition when he/she encounters the noun phrase. However, this study also found greater processing difficulty at the relative clause verb in ORCs than SRCs, which is predicted by memory accounts.

(1) a. The employees that noticed the fireman hurried across the open field.
    b. The employees that the fireman noticed hurried across the open field.

As further examples, both Vasishth and Drenhaus (2011) and Levy and Keller (2013) have argued that locality effects may appear when high working memory load is experienced; anti-locality effects may be present when the load is low.

In a recent development, Husain et al. (2014) argue that the strong predictability for a head (predicting an exact lexical item) can neutralize the locality effect; locality may manifest itself only when predictability strength is weak, that is, when only a verb phrase is predicted, and not the exact identity of the verb. In their self-paced reading (SPR) study, Husain et al. (2014) used a 2 × 2 design, crossing Predictability and Dependency Distance to investigate locality and anti-locality effects. In the strong predictability conditions, Hindi complex predicates (CPs) were used. In these noun-verb sequences, the noun strongly predicted the upcoming light verb, e.g., the noun *khayaal*, “care,” strongly predicts the verb *rakhnaa*, “put,” in *khayaal rakhnaa*, literally, “care put” (“to take care of”). The weak predictability condition, on the other hand, used the same verb used in the complex predicate, but the noun did not predict the verb. An example is *gitaar rakhnaa*, “guitar put”; “to put (down) a guitar”; here, the verb retains its literal meaning. Thus, when the reader sees *gitaar*, they cannot predict the exact identity of the verb, because many other verbs are possible here (e.g., bought). To summarize, in the strong predictability condition, the noun predicted the exact identity of the verb, while in the weak predictability condition the exact identity of the verb was not predicted with high certainty—although *a* verb was predicted. The second factor, dependency distance, was manipulated by placing one to two adverbials between the nominal predicate/object and the verb in the short condition. The long condition had two to three intervening adverbials. Reading time was measured at the verb. The results showed that CP light verbs were read faster in long vs. short distance conditions, but for the non-CP verb there was a tendency toward a slowdown in long vs. short conditions. Finally, there was weak evidence for an interaction (estimate on the log ms scale: 0.03, Bayesian 95% credible interval [−0.02, 0.07], posterior probability of the effect being greater than 0 was 0.77). That is, there was some indication that with increased distance there was a speedup at the light verb in the CP conditions and a slowdown in the non-CP conditions. Although these results can also be interpreted as showing no interaction, Husain and colleagues suggested that strong predictability of the head could be canceling the locality effect, with the locality effect manifesting itself only when predictability strength was weak.

In the present study, we build on the work by Husain et al. (2014) described above. Husain and colleagues' work suggested that the strength of the predictability may modulate whether locality effects occur or not; we investigate the cross-linguistic generality of this claim using Persian, which, like Hindi, also has a complex predicate construction that allows us to manipulate strong and weak predictability. We turn next to a short discussion of the complex predicate construction in Persian as it relates to our experiments.

## 2. Complex predicates in persian

CPs consist of a sequence containing a non-verbal element (e.g., a noun) and a verb, where the meaning of the sequence is non-compositional (Samvelian, 2001). An example is shown in (2).

(2) Maryam be man latme    zad
    Maryam to  me  damage hit
    ‘Maryam caused damage to me (Maryam harmed me).’

The verb, often called a light verb, lacks sufficient semantic force to function as an independent predicate (Vahedi-Langrudi, 1996; Karimi-Doostan, 1997, 2005) and can be combined with different types of non-verbal items such as nominal, adjectivals or prepositional phrases (Dabir-Moghaddam, 1997).

In our study, we used separable CPs as defined by Karimi-Doostan (2011). According to Karimi-Doostan, a complex predicate can be separated if it satisfies both of the following two conditions: (1) if the nominal part is a noun to which adjectives, demonstratives, and wh-words, etc. can be attributed, and (2) if this noun has an internal argument structure (referring to an action or event). From this perspective, Persian CPs are categorized in three groups: (1) predicative verbal nouns (e.g., *anja:m da:dan*, perform+to give), (2) predicative nouns (e.g., *latme zadan*, damage+to hit), and (3) non-predicative nouns (e.g., *gush da:dan*, ear+to do). Among these three types, only the second one satisfies both of the conditions.

We began by independently validating our assumption that the CPs we used in our experiments are predictable and separable. We first conducted a norming study (a sentence completion task), to establish that the light verbs (of the separated CPs) are highly predictable when the nominal is provided, as compared to non-CP verbs in simple predicate conditions. We then conducted an acceptability rating study to determine how acceptable Persian CPs are when they get separated.

## 3. Norming studies

In order to prepare appropriate stimuli, two norming studies were run. The first study involved offline sentence completion and served to validate (i) whether the identity of the verb in the complex predicate is highly predictable, and (ii) whether the identity of the verb in the control conditions is not predictable.

The second study involved offline acceptability rating; the goal was to choose CPs for our experiments which are separable. That is, we wanted to identify CPs which native speakers would find acceptable even if an intervener occurs between the noun-verb sequence. Instructions for both studies are provided in the supplementary Datasheet 1.

The sentence completion study was carried out to derive the predictions of the expectation account. Previous work on expectation effects suggests that sentence completion data may be useful for this purpose. For example, Levy and Keller (2013) used sentence completion data to complement their corpus analyses for deriving their predictions. In their study, the key issue was whether the intervening material (e.g., a dative marked NP) leads to a prediction of a dative verb. Their Table 4 shows that the intervening material sharpened the expectation for the type of verb predicted. This shows that sentence completion data can be used to determine empirically whether the prediction for a specific verb or a verb type is sharpened by intervening material; in the Levy and Keller case, it makes sense that the intervener sharpens the expectation, but clearly the nature and content of the intervening phrases will be crucial in determining whether expectations are sharpened (Konieczny, 2000; Grodner and Gibson, 2005)^2^. Similarly, Husain et al. (2014) used sentence completion to establish that the identity of the verb in a complex predicate is highly predictable given the preceding context, but the identity of the verb in a simple predicate is not (see their Table 4). A third example is Jäger et al. (2015); they used both corpus data and sentence completion to establish that a sentence starting with a determiner, classifier, and an adverb leads to the prediction of a relative clause continuation in Chinese, and that the conditional probability of a subject relative continuation is higher than that of an object relative continuation (see their Table 2). Given these previous results, we assume that sentence completion data is informative about the predictions of the expectation-based account.

### 3.1. Sentence completion studies

Two groups (32 participants each) of Persian native speakers, who did not take part in any of the other experiments, participated in two sentence completion pre-tests in which they were asked to complete the sentences after they were presented the sentence fragment until the pre-critical word. For example, as shown in example 3, subjects were shown incomplete sentences which they had to complete; in this example, the missing verb is shown in parentheses. The participants were allowed to complete the sentence with as many words as they wanted, but our interest was only in the first word that they would write, which would most likely be a verb. This allowed us to calculate the proportion of continuations in which the exact verb was produced.

(3) a. Ali a:rezouyee    bara:ye man (kard)     …
    Ali wish-INDEF for      1.S   (do-PST …
    ‘Ali (made) a wish for me …’
  b. Ali a:rezouyee     ke     besya:r doost-da:sht-am
    Ali wish-INDEF that    a lot     like-1.S-PST
    bara:ye man (kard)    …
    for       1.S (do-PST) …
    ‘Ali (made) a wish that I liked a lot for me …’

The materials were exactly the same as the ones used in the experiments presented below. For the Experiment 1 items, the average prediction accuracy for the exact verb in the strong predictability conditions was 64.46% for the short condition and 59.44% for the long condition; for the Experiment 2 items, it was 65.28 and 62.85% for the short and long conditions respectively. By contrast, the average prediction accuracy for the exact verb in the weak predictability conditions in Experiment 1 was 35.42 and 34.03% for the short and long conditions; and in Experiment 2, it was 36.36 and 30.21% for the short and long conditions. As shown in Tables 1, 2, an analysis using Bayesian generalized linear mixed models with a binomial link function shows a main effect of predictability in both the first experiment and the second experiment.

**Table 1:** **Model results from the Bayesian linear mixed model for the sentence completion study (Experiment 1)**.

**Comparison**	**Mean**	**Lower**	**Upper**	***P*(*b* < 0)**
Intercept	−0.2055	−0.8375	0.407	0.744
Distance	−0.1584	−0.4709	0.143	0.8548
Predictability	0.9635	0.3184	1.6186	0.0025
Distance × Predictability	−0.1246	−0.4688	0.216	0.766

*Shown are the mean and 95% uncertainty intervals, and the probability of the parameter being less than 0*.

**Table 2:** **Model results from the Bayesian linear mixed model for the sentence completion study (Experiment 2)**.

**Comparison**	**Mean**	**Lower**	**Upper**	***P*(*b* < 0)**
Intercept	−0.142	−0.7587	0.4698	0.677
Distance	−0.16	−0.4042	0.0843	0.9035
Predictability	1.1188	0.4919	1.7495	2e-04
Distance × Predictability	0.0365	−0.2102	0.2727	0.3682

*Shown are the mean and 95% uncertainty intervals, and the probability of the parameter being less than 0*.

In the Bayesian models, we used weakly informative priors for the fixed effects (a Student t-distribution with 2 degrees of freedom), and for the random effects (a so-called LKJ prior on the correlation matrix of the random effects' variance-covariance matrix). For an introduction specifically for psycholinguistics, see Sorensen and Vasishth (2015); Nicenboim and Vasishth (2016). One way to interpret whether there is an effect of a particular factor in Bayesian (G)LMMs is to check that the 95% uncertainty interval does not contain zero.

As is clear from the mean percentages for each condition, the light verbs used in the complex predicate conditions were relatively predictable, and the heavy verbs used in the simple predicate conditions were relatively unpredictable. It is also clear from this study that, in our materials, increasing the amount of intervening material does not render the upcoming verb more predictable. The additional information provided by the intervening material for predicting the upcoming verb has been suggested by Konieczny (2000) as one possible explanation for shorter reading times at the verb in long- vs. short-distance conditions. Although this proposal is likely to be correct for some constructions (see discussion in Grodner and Gibson, 2005), in our materials, the sentence completion data do not provide any evidence that the intervening words we used in our design sharpen the expectation for the verb^3^.

### 3.2. Acceptability rating of separable vs. inseparable CPs

Because the noun-verb sequences must be separable for our design to work, we also carried out an acceptability rating pre-test to make sure that the separability of the CPs used in our study is acceptable to native speakers. We tested for the acceptability of different types of noun-verb dependencies by interposing a short prepositional phrase between them. Taking Karimi-Doostan's classification of CPs into account, 36 items from each of the three categories were selected and randomized to test 50 native speakers of Persian (these participants did not take part in any other experiments reported here). They were asked to rate the sentences from 1 (unacceptable) to 7 (completely acceptable). Every participant saw all items. The average acceptability ratings for predicative verbal nouns, predicative nouns and non-predicative nouns were 3.23 (first quartile 1, third quartile 5), 6.08 (first quartile 6, third quartile 7), and 3.12 (first quartile 1, third quartile 5) respectively. That is, items with predicative nouns were the most acceptable. We used all the 36 items of the predicative noun condition in our Experiments 1, 2, and 32 items in Experiments 3, 4 (see the Section 6.1 of Experiment 3 for an explanation).

## 4. Experiment 1

### 4.1. Method

#### 4.1.1. Participants

Forty-two participants aged between 17 and 40 years old (mean 24 years) participated in this experiment in Tehran, Iran. All participants were native speakers of Persian and were unaware of the purpose of the study. This study was carried out in accordance with the Helsinki Declaration, and letters of consent were obtained from all the participants.

#### 4.1.2. Materials

We created 36 experimental sentences with a 2 × 2 factorial design, manipulating predictability strength and distance between the object noun and verb. The short intervener was a prepositional phrase and the long intervener was a relative clause added before the prepositional phrase. In order to mask the experiment, we included 100 filler sentences with varying syntactic structures (see supplementary materials). Here is an example of the target sentences:

(4) a. Strong predictability, short distance (PP)
    Ali a:rezouyee    bara:ye man kard      va…
    Ali wish-INDEF for      1.S   do-PST and…
    ‘Ali made a wish for me and…’
  b. Strong predictability, long distance (RC+PP)
    Ali a:rezouyee     ke   besya:r doost-da:sht-am
    Ali wish-INDEF that a lot      like-1.S-PST
    bara:ye man kard      va…
for       1.S  do-PST and…
    ‘Ali made a wish that I liked a lot for me and…’
  c. Weak predictability, short distance (PP)
    Ali shokola:ti           bara:ye man xarid        va…
    Ali chocolate-INDEF for    1.S   buy-PST and…
    ‘Ali bought a chocolate for me and …’
  d. Weak predictability,   long distance (RC+PP)
    Ali shokola:ti              ke    besya:r doost-da:sht-am
    Ali chocolate-INDEF that  a lot      like-1.S-PST
    bara:ye man xarid        va…
    for       1.S   buy-PST and…
    ‘Ali bought a chocolate that I liked a lot for me and….’

The critical region is the verb (*kard* and *xarid*).

Each sentence (including fillers) was followed by a yes/no comprehension question which targeted different thematic roles in the sentence. Half the questions had a yes answer and half had a no answer. The questions used for the target sentences are provided in the supplementary material.

#### 4.1.3. Procedure

Participants were tested individually using a PC. They were explained the task before they performed the SPR experiment. The participants were instructed to read for comprehension in a normal manner and had a practice session of five sentences. All the sentences were displayed on a single line and were presented in 22 pt Persian Arial font using Linger software (http://tedlab.mit.edu/~dr/Linger/). In order to read each word of a sentence successively in a moving window display, participants had to press the space bar; then the word seen previously was masked and the next word was shown. After each sentence, they were asked to answer a comprehension question to ensure that the participants paid attention to the complete sentence.

#### 4.1.4. Data analysis

The data analysis was conducted in the R programming environment (R Development Core Team, 2013), using Bayesian hierarchical (so-called linear mixed) models using Stan (Stan Development Team, 2014; Gabry and Goodrich, 2016). Sum contrasts were used to code main effects and interactions. In addition, a nested contrast was defined for a secondary analysis in order to look at the effect of distance in CPs vs. the control conditions separately; these were also coded as sum contrasts. We fit full variance-covariance matrices for participants and items (the so-called maximal model, Barr et al., 2013; Bates et al., 2015). All data and code are available from http://www.ling.uni-potsdam.de/~vasishth/code/SafaviEtAl2016DataCode.zip.

### 4.2. Predictions

Based on the Husain et al. (2014) results, in Experiment 1, we expected that increasing noun-verb distance would lead to faster reading time at the verb in the strong predictable conditions, but slower reading time in the weak predictable conditions. Thus, we expected to obtain a cross-over interaction.

The memory based accounts (Just and Carpenter, 1992; Gibson, 2000; Lewis and Vasishth, 2005) predict that increasing distance should lead to a slowdown at the verb; these accounts make no predictions about the strength of predictability.

There are two alternative predictions possible for the expectation account, depending on how one operationalizes expectation. First, if sentence completion probabilities are a reasonable proxy for conditional probabilities—and the previous research reported above (Levy and Keller, 2013; Husain et al., 2014; Jäger et al., 2015) suggests that they may be—then we predict (a) no difference in reading time at the verb as a function of distance, and (b) faster reading time at the verb in the strong predictable conditions than the weak predictable conditions. Prediction (a) arises because, in the sentence completion data, we see no effect of distance on the predictability of the upcoming verb, in either the strong or weak predictability conditions; prediction (b) arises due to the difference in predictability of the exact verb that we see in the strong vs. weak predictability conditions (see the results of the sentence completion studies).

An alternative possible prediction of the expectation account is that increasing distance should facilitate processing at the verb. Surprisal predicts facilitation with increasing distance whenever distance causes the number of possible parses to decrease; this decrease in the number of possible parses leads to the probability mass being reassigned among the remaining parses. In our materials, when a participant reads the noun in the noun-verb complex predicate, they are expecting the light verb with high probability (nearly 1). However, in the long distance condition, the next word begins a relative clause; this leads to an expectation that the light verb will appear *after* the relative clause verb. But what appears after the relative clause verb is a PP that modifies the upcoming light verb. For a facilitation to be predicted in this long-distance condition by the surprisal metric, it would have to be the case that the conditional probability of the light verb following the RC and PP would be higher than the conditional probability of the light verb in the short-distance (PP) condition. In order to get a sense of how the conditional probabilities change in the noun-light verb conditions as a function of distance, we extracted all light verb sentences from a Persian corpus (Seraji, 2015) and then counted, for different numbers of modifying phrases, the proportion of cases that a verb followed the intervening phrase. For example, in a Persian sentence such as *John in the morning went*, there is one intervening phrase, the PP. As shown in Table 3, we find that the conditional probability of the verb appearing next is always high, but goes to 1 with increasing distance. This suggests that in general, increasing distance tends to sharpen the expectation for an upcoming verb. We also did this calculation using the number of intervening words as a metric, rather than the number of intervening phrases. The result, shown in Table 4, is substantially the same as in Table 3. Of course, these corpus counts don't give us any direct information about the predictions regarding our particular experiment design.

**Table 3:** **The conditional probability of a light verb appearing given the complex predicate noun and *n* intervening phrases between the noun and the light verb**.

***n* Intervening phrases**	**Probability of verb**
0	3826∕4003 = 0.95
1	131∕133 = 0.98
2	28∕31 = 0.90
3	5∕5 = 1
4	2∕2 = 1
6	1∕1 = 1

**Table 4:** **The conditional probability of a light verb appearing given the complex predicate noun and *n* intervening words between the noun and the light verb**.

***n* Intervening words**	**Probability of verb**
0	3826/4003 = 0.96
1	104/104 = 1
2	36/39 = 0.92
3	4/5 = 0.8
4	9/10 = 0.9
5	3/3 = 1
6	3/3 = 1
7	1/1 = 1
8	2/2 = 1
10	2/2 = 1
12	1/1 = 1
13	1/1 = 1
14	1/1 = 1

Regarding the strong vs. weak predictability conditions, note that the expectation account of Hale and Levy does not predict that processing should be facilitated when the exact identity of the upcoming verb is predicted (strong predictability condition), compared to the case when just some verb is predicted (weak predictability condition). This is because the surprisal metric is usually calculated using the conditional probability of the part-of-speech (verb) given preceding context, and this will be the same in both the strong and weak predictability conditions. However, it is possible to subsume the difference between strong and weak predictability under the surprisal account by reframing the conditional probabilities in terms of the exact identity of the verb. In this case, the expectation account would predict faster reading times in the strong predictability conditions compared to the weak predictability conditions, regardless of distance.

To summarize, regarding the distance manipulation, the expectation account predicts either no effect or a facilitation at the verb as a function of distance; and regarding the predictability manipulation, the expectation account (appropriately formulated to include the conditional probability of the exact lexical item predicted) would predict a main effect of predictability.

### 4.3. Results

#### 4.3.1. Comprehension accuracy

Participants answered correctly on average 93% of all comprehension questions (excluding fillers). Accuracy was 91, 94, 95, and 91% respectively for the four conditions in (1). As shown in Table 5, a Bayesian generalized linear mixed model of the binary responses showed no evidence for an effect of distance or predictability, or an interaction between predictability and distance.

**Table 5:** **Means, 95% uncertainty intervals, and *P*(*b* < 0), the probability of the estimate being less than 0, in the question-response accuracy analysis for Experiment 1**.

**Comparison**	**Mean**	**Lower**	**Upper**	***P*(*b* < 0)**
Intercept	3.3271	2.8738	3.8724	0
Distance	0.033	−0.3242	0.4033	0.4212
Predictability	−0.174	−0.6128	0.2239	0.8002
Distance × Predictability	0.158	−0.1147	0.4385	0.1345

#### 4.3.2. Reading time

**Figure 1:**
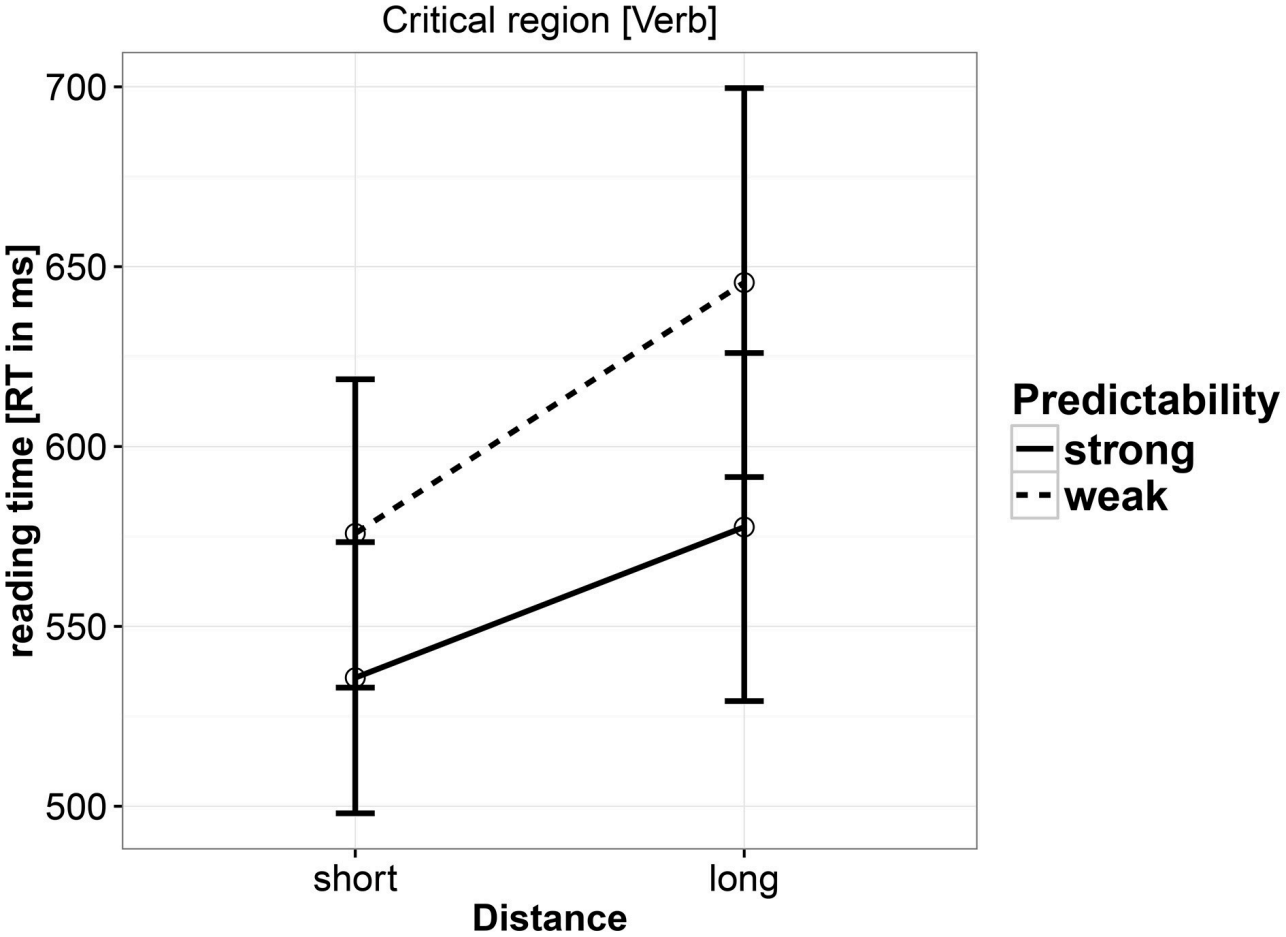
**Reading times at the critical verb in Experiment 1**.

Reading times (RTs) were analyzed at the verb. As shown in Table 6 and Figure 1, there was a main effect of distance, such that increasing distance led to longer reading times. There was also a main effect of predictability: the complex predicate conditions were read faster overall. A weak interaction was also seen: stronger locality effects were seen in the control conditions than in the complex predicate conditions. A nested analysis shows that the distance effect was driven by the control (weak predictability) condition. The estimates for the strong predictability condition were coef. = 0.0218, [−0.0094, 0.0524], *P*(*b* < 0) = 0.0875); and the estimates for weak predictability were coef. = 0.0581, 95% uncertainty intervals [0.0261, 0.0912], *P*(*b* < 0) = 2e-04.

**Table 6:** **Means, 95% uncertainty intervals, and *P*(*b* < 0), the probability of the estimate being less than 0, in the reading time analysis for Experiment 1**.

**Comparison**	**Mean**	**Lower**	**Upper**	***P*(*b* < 0)**
Intercept	6.2434	6.1644	6.3226	0
Distance	0.0397	0.0174	0.0619	2e-04
Predictability	−0.0328	−0.0566	−0.0096	0.998
Distance × Predictability	−0.0179	−0.0405	0.0046	0.942

### 4.4. Discussion

Experiment 1 found a main effect of predictability such that the strong predictability conditions were read faster than the weak predictability conditions, and a main effect of distance, such that the short conditions were read faster than the long conditions. A nested contrast showed that this effect of distance was driven by the weak predictability conditions, i.e., reading time at the verb in condition c was faster than the reading time in condition d. A weak interaction suggests that the locality effect may be somewhat stronger in the weak predictability condition. The suggestion of an interaction seems to provide only weak support, if any, for the idea that strong predictability can at least attenuate locality effects (Husain et al., 2014). The overall effect of distance is consistent with memory-based accounts, which correctly predict a slowdown at the verb in the long conditions, i.e., a main effect of distance. However, as the nested comparison shows, the main effect of distance is driven only by the weak predictability (non-complex predicate) conditions. Memory-based theories would be unable to explain this because they predict a slowdown in long conditions irrespective of predictability strength. However, note that the absence of an interaction makes this absence of a distance effect in the strong predictability conditions difficult to interpret. The expectation account's prediction regarding distance, that increasing the argument-verb distance would either have no effect or result in a facilitation, was clearly not validated; however, the main effect of predictability is consistent with a version of the expectation account that uses the conditional probability of the exact lexical item (verb) appearing given the preceding context.

Our original motivation for this study was to attempt a replication of the Husain et al. (2014) findings. The results are not entirely inconsistent with those of Husain et al. (2014), but they are also not a strong validation of the expectation-memory cost tradeoff posited in that paper. As in the Husain et al. study, we see a main effect of predictability driven by the complex predicate condition. This effect could be explained in terms of reduced retrieval cost at the verb due to its high expectation. An obvious confounding factor here is that the verbs in the strong vs. weak predictability conditions are not identical; this prevents us from ruling out the possibility that low-level differences in the verbs might be responsible for the facilitation due to prediction strength.

We turn next to Experiment 2, in which we manipulate the type of intervener. Here, in the long distance condition, instead of a relative clause and prepositional phrase (PP) intervener, a long PP intervenes. The motivation was to increase distance without having different types of interveners in the short vs. long conditions, as this might be a fairer comparison.

## 5. Experiment 2

### 5.1. Method

#### 5.1.1. Participants

Forty-three participants, with the same criteria as in Experiment 1, participated in this experiment in Tehran, Iran. This study was carried out in accordance with Helsinki Declaration, and consent forms were obtained from all the participants.

#### 5.1.2. Materials

The stimuli and fillers were the same as in Experiment 1 except for the long conditions (b and d), where the intervener was a longer prepositional phrase (PP) instead of the combination of a relative clause and a PP as in the previous experiment. The PP was lengthened using several different structures, all of which had one or more instance of the ezafe possessive marker (Samvelian, 2007):

1. N-ez N-ez N/pronoun/proper name
2. N-ez adj-ez N/pronoun/proper name
3. N-ez adj-ez N
4. N-ez N-ez adj
5. N adj-ez adj
6. superlative adj N N/pronoun/proper name
7. N-ez pronoun

One set of examples using the first type of PP shown above is as follows:

(5) a. Strong predictability, short distance (PP)
    Ali a:rezouyee     bara:ye man kard     va…
     Ali wish-INDEF for       1.S do-PST and…
     ‘Ali made a wish for me and…’
   b. Strong predictability, long distance (longer PP)
     Ali a:rezouyee    bara:ye doost-e    xa:har-e   man
     Ali wish-INDEF for       friend-EZ sister-EZ 1.S
     kard      va…
     do-PST and…
     ‘Ali made a wish for my sister’s friend…’
   c. Weak predictability, short distance (PP)
     Ali shokola:ti              bara:ye man xarid        va…
     Ali chocolate-INDEF for       1.S   buy-PST and…
     ‘Ali bought a chocolate for me and…’
   d. weak predictability, long distance (longer PP)
     Ali shokola:ti              bara:ye doost-e    xa:har-e
     Ali chocolate-INDEF for        friend-EZ sister-EZ
     man xarid       va…
     1.S  buy-PST and…
     ‘Ali bought a chocolate for my sister’s friend and…’

More details about the PPs are provided in the supplementary materials.

#### 5.1.3. Procedure and data analysis

The procedure and data analysis methodology was the same as Experiment 1.

### 5.2. Predictions

In Experiment 2, the distance manipulation involves lengthening the PP. There are two possible predictions of surprisal. One is that surprisal may predict no difference at the verb; this would be because the end of the PP raises a strong expectation for a verb, and this strong expectation for a verb would be the same in both the short and long PP conditions. Another alternative possible prediction of surprisal is that lengthening the PP could lead to a facilitation. This prediction could hold if increasing distance, counted in terms of the number of intervening words, generally increases the predictability of the upcoming verb; this is a possibility given the corpus counts in Table 4.

### 5.3. Results

#### 5.3.1. Comprehension accuracy

Participants answered 93% of all comprehension questions correctly on average (excluding fillers). The accuracies by condition were 96, 92, 94, and 89% respectively for the four conditions in (2). As shown in Table 7, the Bayesian generalized linear mixed models of the responses showed a main effect of distance, such that accuracies were lower in the long conditions. No effect of predictability strength, and no interaction between predictability strength and distance were found.

**Table 7:** **Means, 95% uncertainty intervals, and *P*(*b* < 0), the probability of the estimate being less than 0, in the question-response accuracy analysis for Experiment 2**.

**Comparison**	**Mean**	**Lower**	**Upper**	***P*(*b* < 0)**
Intercept	3.1092	2.7277	3.5353	0
Distance	−0.4246	−0.7556	−0.133	0.9972
Predictability	0.1798	−0.1871	0.5605	0.157
Distance × Predictability	−0.0742	−0.3478	0.1832	0.7098

#### 5.3.2. Reading time

**Figure 2:**
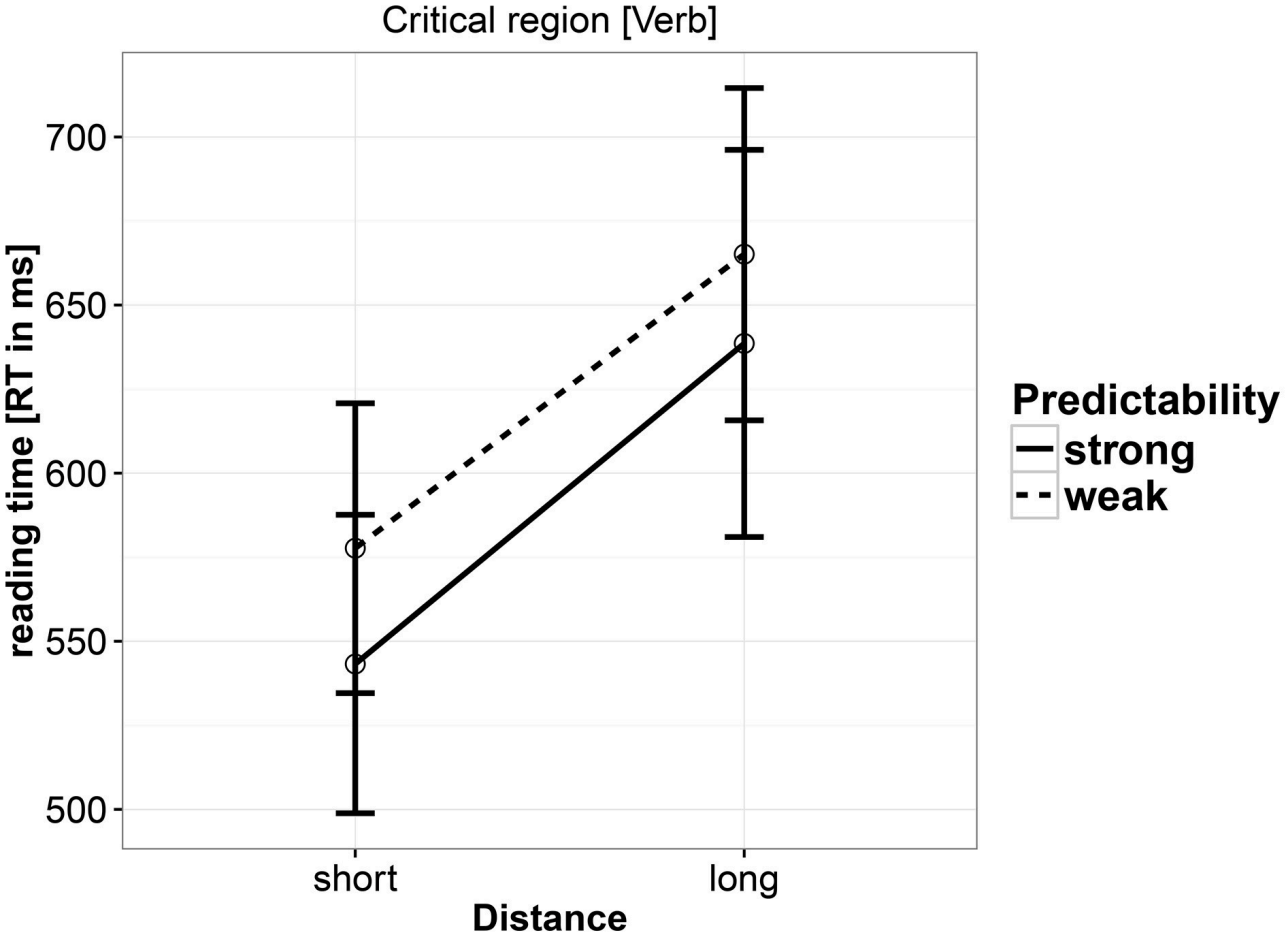
**Reading times at the critical verb in Experiment 2**.

As shown in Table 8 and Figure 2, the results showed a main effect of distance, with long distance conditions being read slower. There was only a weak effect of predictability, with the strong predictability condition being read faster than the weak predictability condition. No interaction was found between predictability and distance. A nested contrast showed that the distance effect is seen in both strong predictability (coef. = 0.0623, [0.0274, 0.0965], *P*(*b* < 0) = 0) and weak predictability (coef. = 0.0475, [0.0098, 0.085], *P*(*b* < 0) = 0.0078) conditions.

**Table 8:** **Means, 95% uncertainty intervals, and *P*(*b* < 0), the probability of the estimate being less than 0, in the reading time analysis for Experiment 2**.

**Comparison**	**Mean**	**Lower**	**Upper**	***P*(*b* < 0)**
Intercept	6.2676	6.1867	6.3488	0
Distance	0.0547	0.0269	0.0827	0
Predictability	−0.0203	−0.0417	0.0013	0.966
Distance × Predictability	0.0077	−0.016	0.0318	0.2585

### 5.4. Discussion

In this experiment, we replicated the locality effects found in Experiment 1, but we no longer see a weakening of the locality effect that was seen in Experiment 1 (a marginal interaction was found in Experiment 1). Nested contrasts showed that locality effects are equally strong in both the strong and weak predictability conditions. In Experiment 2, we also see an effect of predictability, with the strong predictable verb being read faster. Thus, regarding the distance manipulation, the working-memory account's prediction is validated, and the expectation-based account's prediction is not supported. The main effect of predictability does furnish evidence consistent with the expectation-based account.

A secondary analysis was conducted to compare the strength of the locality effect in the two experiments, and to determine whether an interaction between distance, predictability and experiment was present. The between-participant factor experiment was coded using sum coding: Experiment 1 was coded −1, and Experiment 2 was coded +1 (further details are available in the supplementary materials). The results are shown in Table 9. There isn't any convincing evidence for an interaction between distance and experiment; there is only weak evidence for a larger effect of distance in Experiment 2. We cannot therefore argue for a qualitative difference in the distance effects found in Experiments 1 vs. 2.

**Table 9:** **Comparison of Experiments 1 and 2**.

	**Mean**	**Lower**	**Upper**	***P*(*b* < 0)**
Intercept	6.2578	6.1974	6.3198	0
Distance	0.0475	0.0307	0.0647	0
Predictability	−0.0266	−0.0442	−0.0078	0.9958
Expt	0.0138	−0.0425	0.0653	0.299
Distance × Predictability	−0.0054	−0.0203	0.0101	0.761
Distance × Expt	0.0073	−0.0075	0.0219	0.1558
Predictability × Expt	0.0063	−0.0069	0.0193	0.171
Pred × Dist × Expt	0.0128	−0.0012	0.0264	0.0357

In Experiment 2, the intervener was a long, uninterrupted prepositional phrase whereas in Experiment 1, the intervener consisted of a short RC followed by a PP. One can speculate as to why Experiment 2 shows equally strong distance effects in both predictability conditions: processing a single long intervening phrase may be harder than processing two different phrases because it may be harder to chunk a single long phrase compared to two shorter phrases; this is predicted by the Sausage Machine proposal of Frazier and Fodor (1978). If this is correct, then the complexity of the intervener may indeed be a relevant factor in determining whether strong expectation can weaken locality effects. It is possible to test this claim by using an intervener that is much easier to process; an example would be an adverb containing no noun phrases.

We were motivated by the recent replication crisis in psychology (Open Science Collaboration, 2015) to attempt to replicate our results using a different method. Furthermore, replications using ET would be very informative because it is possible that SPR overburdens the working-memory system in an unnatural manner. If this is the case, one prediction would be that the ET data would not necessarily show locality effects. We describe these experiments next.

## 6. Experiment 3

### 6.1. Method

#### 6.1.1. Participants

Forty participants, with the same criteria for inclusion as in the previous experiments, participated in the ET study in University of Potsdam, Germany.

#### 6.1.2. Materials

The experimental items were exactly the same as Experiment 1 (SPR), except that the following four items from Experiment 1 were removed: item id 5, *sheka:yat kardan* (complain + to do), item id 9, *sahm bordan* (share + to win), item id 26, *pishraft kardan* (progress + to do), and item id 32, *hes kardan* (feel + to do). The reason for removal was that the results of the sentence-completion studies suggested that these light verbs had lower predictability than the other light verbs in the stimuli. It could be that this lower predictability is due to the existence of some other alternative light verbs with which the nominal part can combine to make other possible CPs. The last two CPs also had a lower acceptability rating (item 26 had 4.7, and item 32 had 3.5). As a consequence, in our ET study, we had thirty-two experimental items and 64 fillers. All items, including fillers are available in the supplementary materials.

#### 6.1.3. Procedure

An ET study was prepared using Experiment-Builder software, and participants' eye-movements were recorded using an EyeLink 1000 tracker, with a connection to a PC. Before the experiment started, the participants were instructed to read the sentences silently at a normal pace and had a practice block consisting of five sentences. After answering the comprehension questions of the practice block, they were provided with feedback indicating whether or not the answer was correct. A 21-inch monitor was placed 60 centimeters from the participants' eyes. In order to reduce head movements, the participants were asked to use the chin-rest. They viewed the sentences with both eyes, but only the right eye was recorded. The items were presented in one line and in 18 points Persian Arial font (from right to left). First, they had to fixate on a dot at the right edge of the screen so that the sentence appeared. After they finished reading, they had to fixate on the dot in the bottom left corner of the screen; once they fixated on the dot, the comprehension question was presented. Unlike the practice items, they were not provided with any feedback. Calibration was performed at the beginning of the experiment, after their 5-min break (which occurred after they had were halfway through the experiment), and whenever it was necessary.

#### 6.1.4. Data analysis

Raw gaze duration data was obtained using the Data Viewer software^4^. This data was then processed to get different ET measures using the em2 package (Logačev and Vasishth, 2014). As discussed earlier, Bayesian linear mixed models were used for the analysis. All analyses were carried out using log-transformed data. Zero ms reading times were removed before carrying out the analysis.

### 6.2. Results

#### 6.2.1. Comprehension accuracy

On average, participants correctly answered 92% of the target comprehension questions. Mean accuracy by condition was 91 % for condition a, 91% for condition b, 95% for condition c, and 89% for condition d. We found no effects of distance and predictability, and no interaction.

#### 6.2.2. Reading time

The critical region was the verb, as in Experiments 1 and 2. The same sum contrast coding was used as in Experiments 1 and 2; in addition, nested contrast coding was used to investigate the effect of distance within the two predictability conditions. We present results for first-pass reading time and regression path duration.

**Figure 3:**
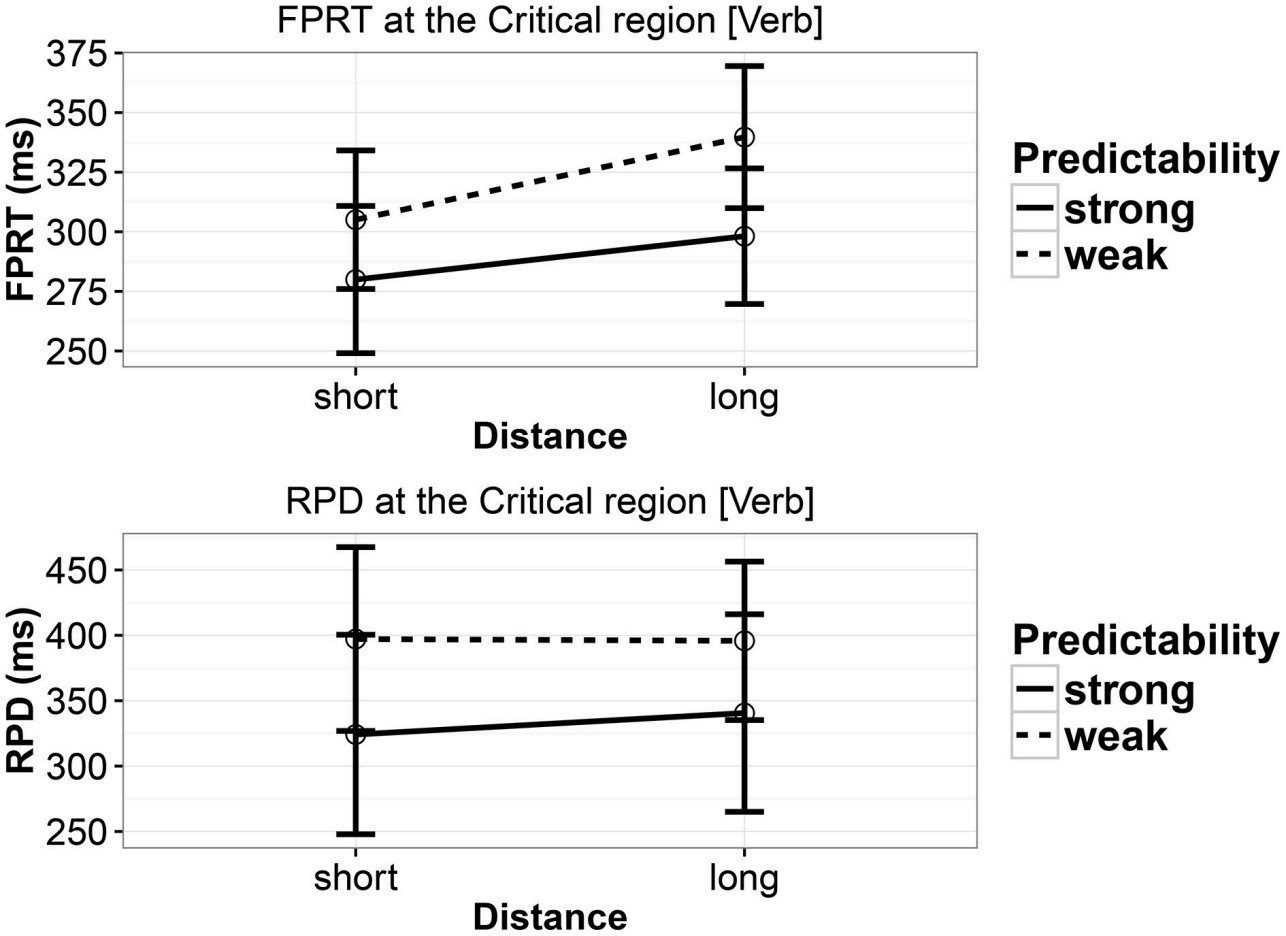
**First-pass reading time and regression path duration in Experiment 3 at the critical verb**. Error bars show 95% confidence intervals.

The effect of predictability, seen in Experiments 1 and 2, is also present in first-pass reading time (FPRT) and regression path duration (RPD); the strong-predictability conditions had shorter reading times. There was also an effect of distance in FPRT but only a weak effect in RPD; the long-distance conditions had longer reading times. Figure 3 and Table 10 show the details of the analyses. A nested contrast showed that in FPRT the distance effect was present in the weak-predictability conditions (coef. = 0.0613, [0.0155,0.1081], and *P*(*b* < 0) = 0.004); in the strong-predictability conditions the effect was weak (coef. = 0.0423, [−0.0022,0.0865], and *P*(*b* < 0) = 0.0318). RPD showed only a weak effect of distance within the two predictability levels. For the weak-predictability level, coef. = 0.0359, [−0.0169,0.0884], *P*(*b* < 0) = 0.0891; and for the strong-predictability level, coef. = 0.0294, [−0.0305,0.0883], *P*(*b* < 0) = 0.1596.

**Table 10:** **Means, 95% uncertainty intervals, and *P*(*b* < 0), the probability of the estimate being less than 0, in the reading time analysis for Experiment 3**.

**ET measure**	**Comparison**	**Mean**	**Lower**	**Upper**	***P*(*b* < 0)**
FPRT	Intercept	5.623	5.5627	5.6833	0
	Distance	0.0504	0.0123	0.0868	0.0062
	Predictability	−0.0522	−0.0844	−0.0189	0.9968
	Distance × Predictability	−0.01	−0.039	0.0196	0.7455
RPD	Intercept	5.7286	5.646	5.8105	0
	Distance	0.0331	−0.0139	0.0814	0.074
	Predictability	−0.0754	−0.1248	−0.0265	0.9992
	Distance × Predictability	−0.0032	−0.0374	0.0316	0.5742

### 6.3. Discussion

In the ET Experiment 3, we replicated the locality effects found in the Experiment 1 in first-pass reading time. Nested contrasts showed that the locality effect appeared in weak-predictability conditions, which is similar to the result in Experiment 1. A main effect of predictability was found in FPRT and RPD, replicating the effect in Experiment 1.

Since we failed to find any interaction between predictability and distance, we cannot conclude, as Husain et al. (2014) did, that expectation effects can cancel locality effects. The locality effects are consistent with working memory accounts (Gibson, 2000; Lewis and Vasishth, 2005) and inconsistent with the distance-based predictions of the expectation account (Levy, 2008). As in the SPR experiments, we have evidence consistent with a version of the expectation account that predicts that strong predictability conditions will be read faster than the weak predictability conditions.

In sum, the main result in Experiment 3 is that we have replicated the locality effect and the facilitation due to strong predictability.

## 7. Experiment 4

### 7.1. Method

#### 7.1.1. Participants

Forty participants, with the same criteria as in the previous experiments, participated in the ET study in Golm campus, University of Potsdam, Germany.

#### 7.1.2. Materials

The experimental items were exactly the same as Experiment 2 (SPR), but with 32 items (see the explanation for Experiment 3 regarding the four items that were removed). The experimental items were complemented with 64 filler sentences with varying syntactic structures (see supplementary materials).

#### 7.1.3. Procedure and data analysis

The procedure and data analysis were exactly the same as Experiment 3 (ET).

### 7.2. Results

#### 7.2.1. Comprehension accuracy

On average, participants answered 90% of comprehension questions correctly. They had 94% response accuracy for condition a, 88% for condition b, 94% for condition c, and 86% for condition d. None of the factors had an effect on accuracy.

#### 7.2.2. ET measures

**Figure 4:**
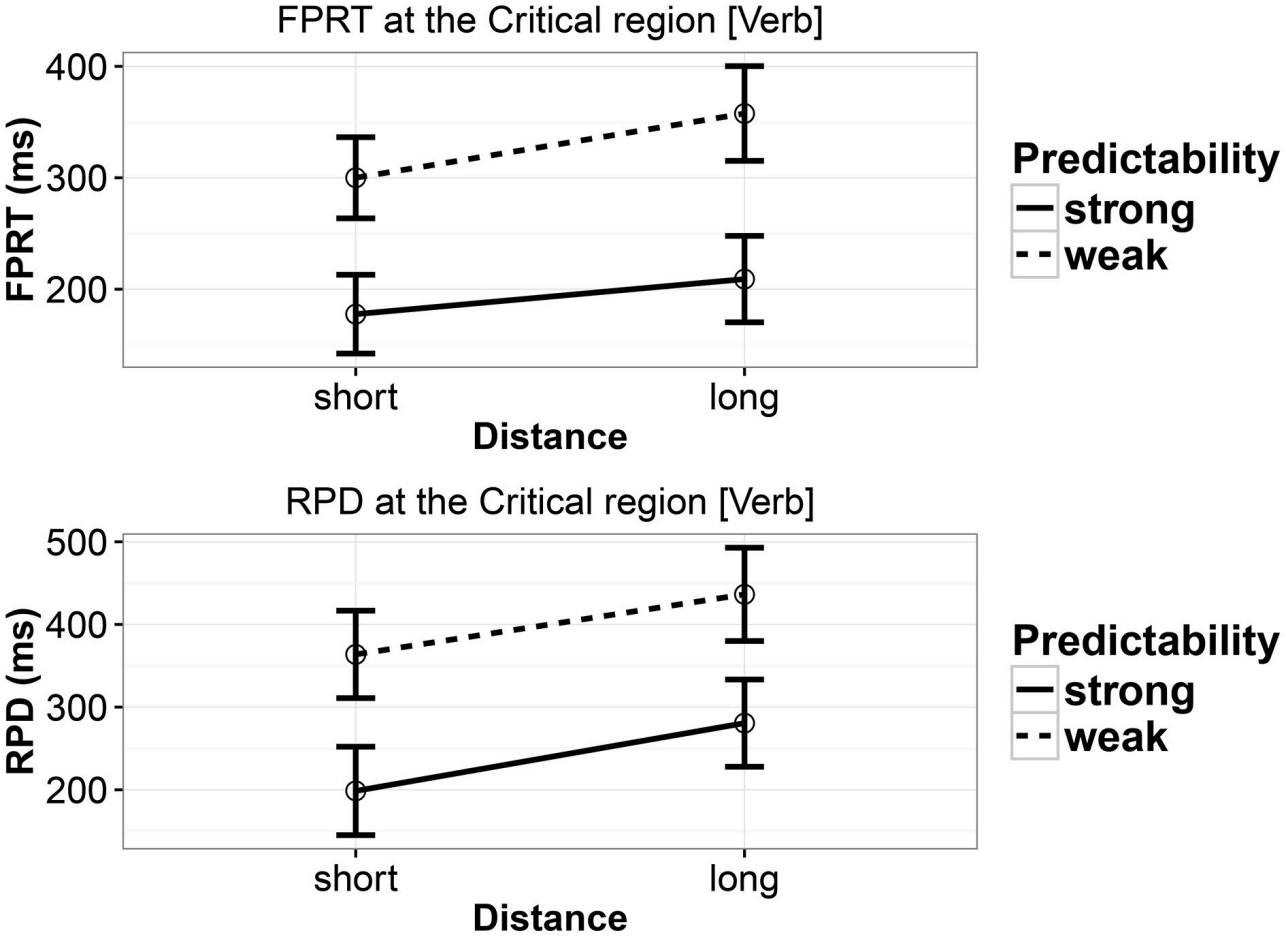
**First-pass reading time and regression path duration in Experiment 4 at the critical verb**. Error bars show 95% confidence intervals.

The reading times at the critical region are summarized in Figure 4. Unlike Experiment 3, in the current experiment, we found effects of distance and predictability in both the measures (see Table 11). In other words, in the two measures reported, the long conditions (b and d) were read slower than the short conditions (a and c), and the weak predictability conditions (c and d) were read slower than the strong predictability conditions (a and b). None of the measures showed any interaction between predictability and distance.

**Table 11:** **Means, 95% uncertainty intervals, and *P*(*b* < 0), the probability of the estimate being less than 0, in the reading time analysis for Experiment 4**.

**ET measure**	**Comparison**	**Mean**	**Lower**	**Upper**	***P*(*b* < 0)**
FPRT	Intercept	5.6731	5.6015	5.7448	0
	Distance	0.0557	0.0099	0.1013	0.0082
	Predictability	−0.1079	−0.1512	−0.0638	1
	Distance × Predictability	−0.0046	−0.0397	0.0315	0.6088
RPD	Intercept	5.7958	5.7113	5.8799	0
	Distance	0.0767	0.0316	0.1225	5e-04
	Predictability	−0.1108	−0.1588	−0.0626	1
	Distance × Predictability	0.0089	−0.0381	0.0547	0.3452

Nested comparisons showed that in first-pass reading time, the locality effect was seen in the strong-predictability condition (coef. = 0.0507, [0.0011, 0.1003], *P*(*b* < 0) = 0.022), but there was a weaker tendency toward a locality effect in the weak-predictability condition (coef. = 0.061, [−0.0046, 0.1261], *P*(*b* < 0) = 0.0355). In regression-path duration, both strong- and weak-predictability conditions showed a locality effect (strong-predictability: coef. = 0.0858, [0.0253, 0.1492], *P*(*b* < 0) = 0.0031; low-predictability: coef. = 0.0675, [0.0027, 0.1317], *P*(*b* < 0) = 0.0211.

### 7.3. Discussion

Experiment 4 replicated the results of Experiment 2: a main effect of distance and a main effect of predictability, with no evidence for an interaction. The effects in FPRT and RPD showed essentially the same patterns as in the first ET study. However, the locality effects were even stronger, in the same way that the second SPR study showed stronger locality effects. Also, these effects are equally strong in both strong and weak predictability conditions, mirroring our finding in the second SPR study.

Overall, regarding the distance manipulation, the results are consistent with memory-based accounts, and inconsistent with the expectation account. The main effect of predictability is consistent with the expectation account, as discussed earlier. In Experiment 4, we don't see any evidence consistent with the Husain et al. (2014) proposal; if anything, the locality effect is *stronger* in the strong-predictability conditions.

## 8. General discussion

**Figure 5:**
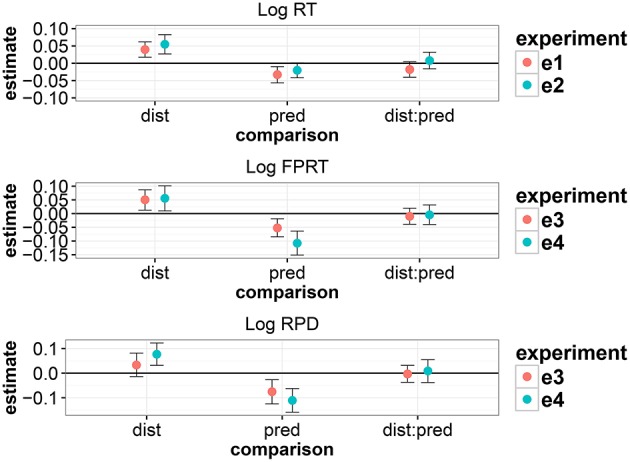
**Summary of the magnitudes of effects (derived from the linear mixed models) across the four experiments**. The error bars show 95% uncertainty intervals and show the range within which we can be 95% certain that the true parameter lies given the data.

As summarized graphically in Figure 5, our main finding from the four Persian studies is that the locality effect predicted by memory accounts is upheld, but there is no evidence for the expectation-based account's prediction of facilitation in longer distance conditions. We consistently see a main effect of predictability, which is consistent with expectation accounts. Finally, there is no compelling evidence in the Persian data that strong expectations cancel locality effects.

There is also suggestive evidence that the complexity of intervening material could strengthen the locality effect: when the intervener is an RC followed by a PP, we see a marginal interaction between distance and predictability, but when the intervener is a single long PP, we see no evidence for an interaction between distance and predictability strength, and we tend to see stronger effects.

We consistently found a main effect of predictability in all four experiments: the strong predictability conditions were read faster at the verb than the weak predictability conditions. This is consistent with the expectation-based account. Since the verbs in the strong and weak predictability conditions are not identical, we cannot rule out the possibility that word frequency or other such low-level factors are responsible for these effects. However, it is plausible that the highly predictable verb is processed faster than the less predictable verb. Thus, the main effect of predictability can be seen as evidence for expectation-based accounts, operationalized in terms of the conditional probabilities of the appearance of the exact verb given the preceding context.

It is possible that we were unable to replicate Husain et al's findings because of the nature of the intervener used in the Persian studies. Unlike, Husain et al. (2014) where the long distance condition had extra adverbials compared to the short condition, in Experiment 1 we have a more complex intervener, a relative clause. Another reason for finding the effects which are different from the study by Husain et al. (2014) could be that in Persian, separating the nominal part of the CP from the light verb occurs relatively rarely, compared to Hindi. There is some support for this in corpus data. Based on the Hindi dependency treebank (Bhatt et al., 2009), the average distance, counted as the number of intervening phrases, between an object and its (heavy) verb is 0.82 (with minimum 0 and maximum 15, and first and third quantiles 0 and 1), and the average distance between a noun and light verb is 0.07 (minimum 0 and maximum 18, with first and third quantiles 0 and 0). In the Persian dependency treebank (Seraji, 2015), the average distance between an object and (heavy) verb is 2.48 (with minimum 0 and maximum 9, and first and third quantiles 1 and 3), while the average distance between a noun and light verb is 0.05 (with minimum 0, and maximum 6, and first and third quantiles 0 and 0). Thus, the adjacency of CPs in Persian is strongly preferred (maximum 6 vs. Hindi's maximum 18), although as validated in the acceptability rating norming study, this separability is apparently acceptable and not considered ungrammatical^5^.

### 8.1. An alternative explanation of locality effects in terms of entropy

Could there be an alternative explanation for the locality effect seen in the four experiments, one that does not invoke greater memory cost in the long-distance conditions? One possibility is that entropy (uncertainty) increases with increasing distance. Entropy is an information-theoretic measure that essentially represents how uncertain we are of the outcome (Shannon, 2001). In the present case, this would translate to our uncertainty about the upcoming verb. If there are *n* possible ways to continue a sentence, and each of the possible ways has probability *p_i_*, where *i* = 1, …, *n*, then entropy is defined (Shannon, 2001) as $-\sum_{i}p_{i}\times \log_{2}(p_{i})$. The entropy associated with the upcoming verb can be calculated using our offline sentence completion data^6^.

#### 8.1.1. Evaluating the effect of entropy

**Figure 6:**
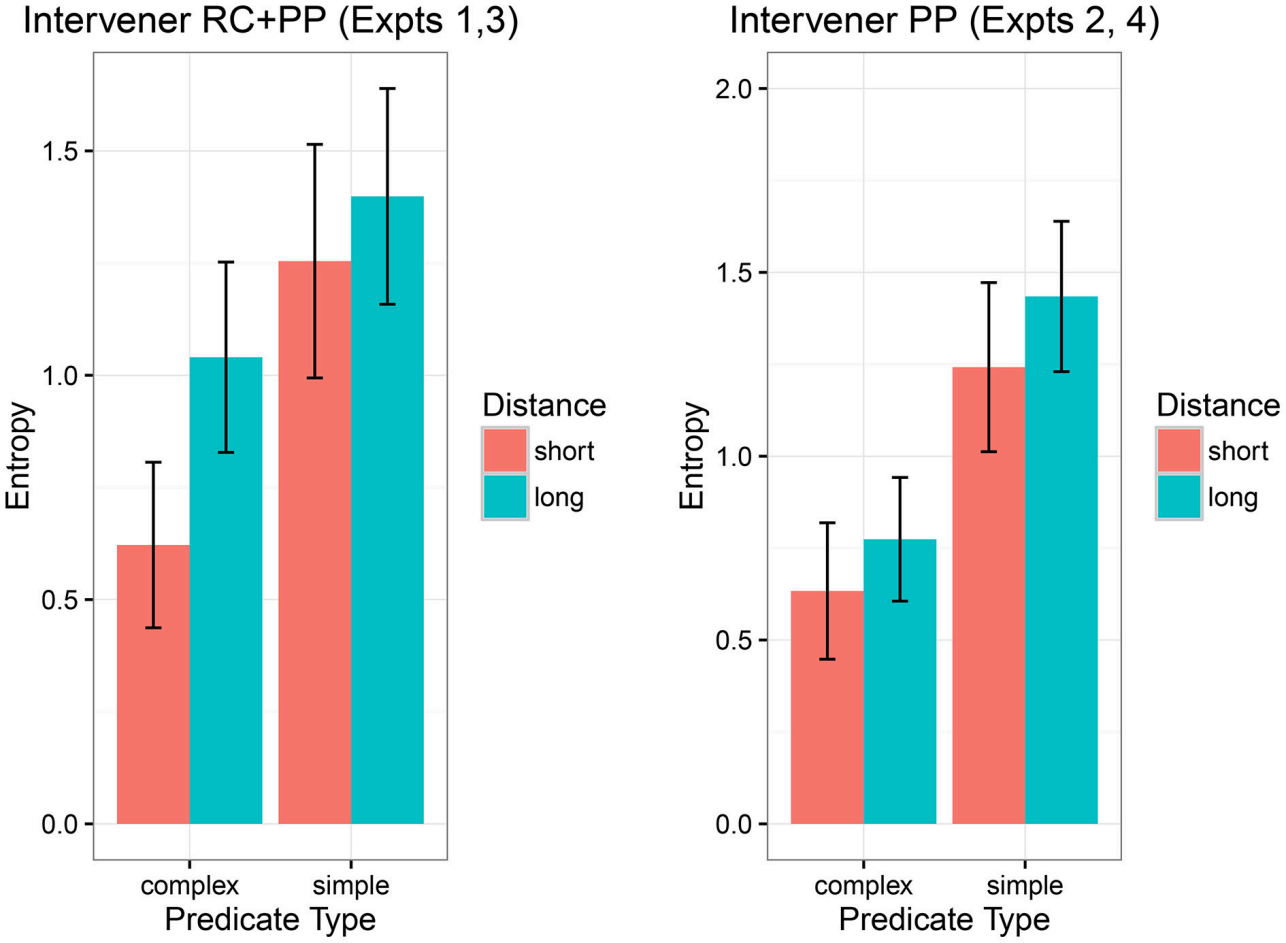
**The estimated entropy (with 95% confidence intervals), computed using the sentence completion data, for the two experiment designs**.

In order to evaluate whether entropy could explain the locality data, we computed entropy for each item in each condition for both experiments. The estimated entropies for each condition in the two experiment designs are shown in Figure 6. It is important to note here that entropy for each condition in Figure 6 is based on only nine data points per condition (we only have 9 × 4 = 36 items); for different items, there is substantial variability in the entropy patterns by condition. Nevertheless, in the figure we can see that in the items used for Experiments 1 and 3, the entropy is higher in the long-distance conditions. The effect of entropy is less clear for the items used in Experiments 2 and 4, because of the relatively wider confidence intervals. Clearly uncertainty is higher in the RC+PP experiment than in the long PP experiment. A closer look at the high predictability conditions shows that the entropy difference between the long and short distance conditions is larger in the RC+PP intervener items than the entropy difference in the long PP intervener items (it is larger by 0.14, with 95% uncertainty intervals −0.01 and 0.28, probability of the difference in entropy being less than 0 is 0.03). This is suggestive—if weak—evidence that the intervening RC may be responsible for creating a greater degree of uncertainty regarding the upcoming verb. This is a bit surprising because stronger locality effects were seen in the long PP experiments.

In order to investigate whether entropy affects reading times at the verb, we fit a maximal Bayesian linear mixed model with predicate type and distance as sum-coded factors, and entropy (centered) as a continuous predictor; all higher order interactions were also included. The dependent variable was log reading time at the critical verb. As shown in Table 12, in Experiment 1, in addition to the effects of predictability and distance, we find an effect of entropy, and an interaction between distance and entropy, such that long distance conditions lead to a greater effect of entropy. None of the other experiments showed any effects of entropy. Thus, although the evidence in favor of entropy is far from overwhelming, a potentially important finding here is that entropy could explain locality effects at least in our Experiment 1. To our knowledge, this is the first demonstration that locality effects may arise due to factors other than memory costs.

**Table 12:** **Model results from the Bayesian linear mixed model for the effect of entropy (apart from other predictors) on log reading times in Experiment 1**.

**Comparison**	**Mean**	**Lower**	**Upper**	***P*(*b* < 0)**
Intercept	6.23	6.16	6.3	0
Predictability	−0.02	−0.04	0	0.95
Distance	0.03	0.01	0.06	0.01
Entropy	0.05	0.02	0.09	0
Pred:Dist	−0.01	−0.03	0.02	0.71
Pred:Entropy	−0.02	−0.06	0.01	0.88
Dist:Entropy	0.04	0.01	0.07	0.01
Pred:Dist:Ent	0	−0.03	0.04	0.45

*Shown are the mean and 95% uncertainty intervals, and the probability of the parameter being less than 0*.

But why does entropy increase in longer-distance dependencies? A possible explanation suggests itself in terms of memory overload causing forgetting. It is possible that the participants forgot that a noun-verb dependency exists in the long-distance complex predicate condition. One prediction of this forgetting-inducing-entropy account would be that in the sentence completion study, participants would tend to produce more ungrammatical continuations in the long-distance condition than the short-distance condition. This is borne out in Experiment 1: the accuracy in the short condition was 97%, and in the long condition it was 92%. A Bayesian generalized linear mixed model was fit with a full variance-covariance matrix for participants and items^7^. The results of the model fit showed a reduction in grammaticality of sentence completions in the long vs. short conditions; the log odds were −0.9436 [−2.042, −0.1284], with a probability of the log odds being negative being 0.99. The sentence completion study corresponding to Experiment 2 (which showed no effect of entropy on reading times) showed no difference in grammaticality of completions; the short and long conditions had grammatical continuations with the proportions 0.97 and 0.98.

Thus, it is possible that in Experiment 1, the increase in entropy is due to participants forgetting the left context partially. Clearly, a planned experiment is called for to investigate this further. An important point to note here is that the increased entropy in the long-distance condition may be a *consequence* of forgetting, not a cause in itself: entropy itself would not predict any increase in ungrammatical continuations, but the forgetting hypothesis does.

#### 8.1.2. Does predictability of an upcoming verb increase with distance?

We showed above that increasing uncertainty about the upcoming verb may explain locality, at least in Experiment 1. One important question that arises, especially in the strong predictability conditions, is the following: does increasing distance nevertheless sharpen the expectation for the verb, as suggested by Konieczny (2000)? In order to address this question, we fit a Bayesian generalized linear mixed model (GLMM) with a logistic link function that investigated the change in probability mass for the target verb as a function of distance in the strong predictability conditions.

For the first sentence completion study (which had the RC+PP intervener in the long condition), in the long-distance condition, the probability of producing the target verb fell: on the log-odds scale, the mean and 95% uncertainty interval were −0.305 [−0.8127, 0.1591] and the posterior probability of the reduction being less than 0 was 0.9. The odds ratio of producing a target verb in the long vs. short condition was 0.74, with 95% uncertainty interval [0.44,1.17]. This means that in the long condition, participants are less likely to produce the target verb, but since the uncertainty interval for the odds ratio includes 1, the reduction in probability of target verb production is possibly unchanged in the short vs. long distance conditions. If anything, there is a weakening of the expectation for the target verb, contrary to the sharpened expectation proposal of Konieczny (2000).

For the second sentence completion study (which had a PP in the long condition), in the long-distance condition, the probability of producing the target verb also fell: the logs odds were −0.17 [−0.5,0.12]; and the posterior probability of the reduction being less than 0 was 0.86. The odds ratio of producing a target verb in the long vs. short condition was 0.84, with 95% uncertainty intervals [0.61, 1.13]. Thus, in the second sentence completion study, there is only weak evidence of a reduction in probability of producing the target verb in the long-distance condition.

To summarize, our sentence completion data for Experiments 1 and 2's strong predictability condition show that increasing distance tends to reduce the proportion of target verbs produced, although the evidence for this reduction is rather weak overall. Our data from Persian therefore seem to go against the suggestion by Konieczny (2000) that increasing distance leads to narrowing down the prediction to the target verb.

Caution is needed in interpreting these results based on the sentence completion data. The biggest issue with the sentence completion data is that it was an offline task; it is difficult to argue that offline completion data can inform us about online processes. It would be much more informative to run an online sentence completion study, forcing participants to make quicker decisions about the sentence completions. Further, most of our findings relating to the sentence completion data are *post-hoc* and based on exploratory analyses. It would also be very informative to carry out sentence completion studies for experiments such as those of Konieczny (2000); Grodner and Gibson (2005); Vasishth and Lewis (2006); Bartek et al. (2011); Vasishth and Drenhaus (2011); Levy and Keller (2013) in order to establish whether increasing distance can weaken expectation cross-linguistically.

In future work it may be worth investigating existing locality effects in English, German, and Hindi from the perspective of forgetting inducing entropy. A further possibility worth investigating is whether entropy reduction (Hale, 2006) rather than entropy can explain the locality effects cross-linguistically. In our Persian experiments, it is possible that the entropy at the word preceding the verb is higher than the entropy at the verb, and it is possible that the reduction in entropy is larger in the long-distance condition. Unfortunately, we have no way to test this in the present design, but future studies could compute entropy reduction empirically in the same way that we computed entropy using sentence completion data. Thus, in principle it is possible that entropy reduction could explain locality effects as well. A related issue that would then arise is whether entropy or entropy reduction furnishes a better explanation for locality effects.

A broader issue that the above discussion raises is, can all intervention effects be explained via an appeal to information-theoretic metrics? Levy (2008) had pointed out that information-theoretic metrics cannot explain all the results relating to intervention effects; he was mainly referring to locality effects, which can only be explained through memory-based accounts. In later work, Vasishth and Drenhaus (2011); Levy and Keller (2013); Levy et al. (2013) also find that both memory and expectation-based accounts are needed to explain the range of observed effects. It is because of the inability of information-theoretic metrics to explain locality effects that Levy (2008) argued for “two-factor” accounts. If entropy or some other entropy-based measure turns out be an explanation for locality effects, can we argue for a simpler account that only appeals to information-theoretic metrics? A major empirical problem for such a reductionist account would be the large range and variety of intervention effects (see Engelmann et al., Manuscript submitted). for a review and computational modeling) that can only be explained through memory-based accounts. Other recent results that would be impossible to explain via a reductionist account are the work by Nicenboim et al. (2015) and Nicenboim et al. (2016). Thus, a reductionist account that assumes that *all* effects can be explained by what is predicted next would always falter when it comes to explaining effects that arise not from predictive processes but from retrieval-based processes.

## 9. Concluding remarks

In conclusion, as regards the distance manipulation, the evidence from Persian is in favor of working-memory accounts, although forgetting-causing-entropy is also a candidate explanation. There is not much evidence from Persian that strong-predictability conditions cancel locality effects, as Husain and colleagues had suggested. Interestingly, there is no evidence in these experiments for the prediction of the expectation account regarding the distance manipulation, that increasing argument-verb distance facilitates processing due to increasing conditional probabilities of the upcoming verb. The suggestion in Levy et al. (2013) that “the verb-medial languages tend to exhibit the general patterns predicted by memory-based theories, whereas verb-final languages tend to exhibit the general patterns predicted by expectation-based theories” seems to be difficult to maintain (also see Husain et al., 2015, for locality effects in Hindi). One implication of our findings from Persian is that locality and expectation effects observed across studies seem to be highly conditional on the language and syntactic construction being considered—broad cross-linguistic generalizations may be difficult to make.

## Author contributions

MS, SV, and SH designed the experiments; MS prepared the items, recruited participants, and conducted all the studies reported; SH conducted all the corpus analyses and extracted the statistics from the corpora; SV, MS, and SH analyzed the data and prepared the final document.

## Funding

This work was supported by the IDEALAB program, and the University of Potsdam. We acknowledge the support of the Deutsche Forschungsgemeinschaft (German Research Foundation) and Open Access Publication Fund of Potsdam University.

### Conflict of interest statement

The authors declare that the research was conducted in the absence of any commercial or financial relationships that could be construed as a potential conflict of interest.

## Footnotes

^1^Another expectation-based account in the literature is the entropy reduction hypothesis or ERH (Hale, 2006); we do not investigate ERH in this paper, but we do discuss a related idea, entropy, in Section 8.

^2^We return to this point in the Section 8, where we discuss the effect of entropy on reading times.

^3^In fact, in our sentence completion data, as discussed in the Section 8, entropy increases with distance.

^4^http://www.sr-research.com/dv.html

^5^These intervening phrases have been computed using dependency treebanks. Consequently, phrasal boundaries are approximations. Also, because of annotation differences between the two treebanks, phrase boundary criteria sometimes differ for the two languages. The phrasal counts lead to the same conclusions regardless of whether one counts intervening phrases or words.

^6^See Linzen and Jaeger (2015) for a recent empirical investigation of entropy in sentence comprehension using corpus data instead of sentence completion data. Linzen and Jaeger calculated entropy in several ways, and also evaluated another metric called entropy reduction (ER), which was proposed by Hale, 2006; however, we cannot evaluate ER here because that would require knowing the entropy for the word preceding the verb.

^7^The predictor (short vs. long condition) was coded using sum contrasts, with the long condition coded as 1 and the short condition as −1; the dependent variable was binary and represented whether a target verb was produced by the participant for a particular item-condition combination or not. Participants and items were specified as partially crossed random factors, and a full variance-covariance matrix was fit for both random effects. The priors for the intercept and slope were the Student's t-distribution with 2 degrees of freedom, allowing a range of approximately −10 to 10 on the log odds scale, with 0 the most likely value. The prior on the variance-covariance matrices was defined via the LKJ prior (Stan Development Team, 2013, 2014) on the correlation matrix; see Sorensen and Vasishth (2015) for a tutorial intended for psycholinguists and cognitive scientists. The model was fit using the stan_lmer function from the rstanrarm package (Gabry and Goodrich, 2016).
