# Supplementary material for: Dependency Resolution Difficulty Increases with Distance in Persian Separable Complex Predicates: Evidence for Expectation and Memory-Based Accounts
Source: Front Psychol. 2016 Mar 30;7:403. doi: 10.3389/fpsyg.2016.00403 (PMC4812816; doi:10.3389/fpsyg.2016.00403)
Supplement: Supplementary file 1 [file DataSheet1.zip › SafaviEtAl2016DataCode/items_fillers/Expt1.glossing.pdf]

Items for Persian Experiment I

1

a.	مریم آرزویی برای من کرد که خیلی زود برآورده شد

b.	مریم آرزویی که همیشه دوست داشتم برای من کرد و خیلی زود برآورده شد

مریم	آرزویی	که	همیشه	دوست	داشتم	برای	من
Maryam	Arezooyee	ke	hamishe	doost	dashtam	baraye	man
Maryam	a wish	that	always	like	had (i)	for	me
Proper name	indef. Noun	relative marker	adverb	pre-verbal noun	verb	prep	pronoun
کرد	و	خیلی	زود	برآورده شد			
kard	va	kheili	zood	baravardeh shod			
did	and	very	soon	came true			
light verb	coordinator	quantifier	adverb	verb			

c.	مریم غذایی برای من پخت که خیلی خوشمزه بود

d.	مریم غذایی که همیشه دوست داشتم برای من پخت و خیلی خوشمزه بود

مریم	غذایی	که	همیشه	دوست	داشتم	برای	من
Maryam	Ghazaayee	ke	hamishe	doost	dashtam	baraaye	man
Maryam	a dish	that	always	like	had (i)	for	me
Proper name	indef. Noun	relative marker	adverb	pre-verbal noun	verb	prep	pronoun
پخت	و	خیلی	خوشمزه	بود			
pokht	va	kheili	khoshmazze	bood			
cooked	and	very	delicious	was			
heavy verb	coordinator	quantifier	adjective	verb			

2

a.	علی آهنگی برای من زد و مرا به سالهای نوجوانی برد

b.	علی آهنگی که مورد علاقه‌ام بود_را برای من زد و مرا به سالهای نوجوانی برد

علی	آهنگی	که	مورد	علاقه ام	بود	را	برای
Ali	Ahangi	ke	morede	alaghe'am	bood	ra	baraye
Ali	a (piece of) music	that	to	my interest	was	ra	for
Proper name	indef. Noun	relative marker	prep	possessive adj + noun	verb	accus. Marker	prep
من	زد	و	مرا	به	سالهای	نوجوانی	برد
man	zad	va	mara	be	salhaye	nojavani	bord
me	hit	and	me- ra	to	years of	teenage	took
obj. pronoun	light verb	coordinator	obj. pronoun + accus. Marker	prep	noun	noun	verb

c.	علی شکلاتی برای من خرید که مزه‌اش حرف نداشت

d.	علی شکلاتی که مورد علاقه‌ام بود_را برای من خرید و مزه‌اش حرف نداشت

علی	شکلاتی	که	مورد	علاقه ام	بود	را	برای
Ali	shokolati	ke	morede	alaghe'am	bood	ra	baraye

Ali	a chokolate	that	to	my interest	was	ra	for
Proper name	indef. Noun	relative marker	prep	possessive adj + noun	verb	accus. Marker	Prep
من	خرید	و	مزه اش	حرف	نداشت		
man	kharid	va	mazze'ash	harf	nadasht		
me	bought	and	its flavor	word	didn't have		
obj. pronoun	Heavy verb	coordinator	possessive adj + noun	noun	verb (negative)		

3

a. مادرم دعا برای من کرد تا همیشه موفق و سلامت باشم

b. مادرم دعایی که بسیار زیبا بود برای من کرد تا همیشه موفق و سلامت باشم

مادرم	دعایی	که	بسیار	زیبا	بود	برای	من
Madaram	do'ayee	ke	besyar	ziba	bood	baraye	man
My mother	a prayer	that	very	beautiful	was	for	me
Possessive adj + noun	indef. Noun	relative marker	quantifier	adjective	verb	prep	pronoun
کرد	تا	همیشه	موفق	باشم			
Kard	ta	hamishe	movaffagh	basham			
Did	so that	always	successful	am			
light verb	subordinator	adverb	adjective	verb			

c. مادرم لباسی برای من دوخت تا زمستان آن را بپوشم

d. مادرم لباسی که بسیار زیبا بود برای من دوخت تا زمستان آن را بپوشم

مادرم	لباسی	که	بسیار	زیبا	بود	برای	من
madaram	Lebasi	ke	besyar	ziba	bood	baraye	man
My mother	a dress	that	very	beautiful	was	for	me
Possessive adj + noun	indef. Noun	relative marker	quantifier	adjective	verb	prep	pronoun
دوخت	تا	زمستان	آن	را	بپوشم		
dookht	Ta	zemestan	an	ra	bepoosham		
sewed	so that	(in) winter	that	ra	wear (i)		
heavy verb	Subordinator	adverb	demonstrative pronoun	accus. Marker	verb		

4

a. حمید تقاضایی از مینا کرد و منتظر جواب شد

b. حمید تقاضایی که کاملا غیر منتظره بود از مینا کرد و منتظر جواب شد

حمید	تقاضایی	که	کاملا	غیرمنتظره	بود	از	مینا
Hamid	taghazayee	ke	kamelan	gheire-montazereh	bood	az	Mina
Hamid	a request	that	completely	Unexpected	was	from	Mina
Proper name	indef. Noun	relative marker	adverb	Adjective	verb	prep	Proper name
کرد	و	منتظر	جواب	شد			
kard	va	montazere	javab	Shod			
did	and	wait (for)	reply	Did			

light verb	coordinator	noun	noun	Verb			

c. حمید جوابی از مینا شنید و شوکه شد

d. حمید جوابی که کاملا غیر_منتظره بود از مینا شنید و شوکه شد

حمید	جوابی	که	کاملا	غیرمنتظره	بود	از	مینا
Hamid	javabi	ke	kamelan	gheire-montazere	bood	az	Mina
Hamid	a reply	that	completely	Unexpected	was	from	Mina
Proper name	indef. Noun	relative marker	adverb	Adjective	verb	prep	Proper name
شنید	و	شوکه	شد				
shenid	va	shokkeh	shod				
heard	and	shocked	got				
heavy verb	coordinator	adjective	verb				

5

a. حسن شکایتی از همسایه‌اش کرد اما بعد از او عذرخواهی کرد

b. حسن شکایتی که اصلا منطقی نبود از همسایه‌اش کرد اما بعد از او عذرخواهی کرد

حسن	شکایتی	که	اصلا	منطقی	نبود	از	همسایه اش
Hassan	shekayati	ke	aslan	manteghi	nabood	az	hamsaye'ash
Hassan	a complaint	that	at all	logical	was not	from	his neighbor
Proper name	indef. Noun	relative marker	quantifier	adjective	verb (negative)	prep	possessive adj + noun
کرد	اما	بعد	از	او	عذرخواهی	کرد	
kard	amma	ba'd	az	oo	ozr-khahi	kard	
did	but	then	from	him	apology	did	
light verb	coordinator	adverb	prep	obj pronoun	noun	verb	

c. حسن داستانی از همسایه‌اش شنید و نمی‌دانست باید آن را باور کند یا نه

d. حسن داستانی که اصلا منطقی نبود از همسایه‌اش شنید و نمی‌دانست باید آن را باور کند یا نه

حسن	داستانی	که	اصلا	منطقی	نبود	از	همسایه اش
Hassan	dastani	ke	aslan	manteghi	nabood	az	hamsaye'ash
Hassan	a story	that	at all	logical	was not	from	his neighbor
Proper name	indef. Noun	relative marker	quantifie r	adjective	verb (negative)	prep	possessive adj + noun
شنید	و	نمی دانست	باید	آن	را	باور کند	یا نه
shenid	va	nemidanest	bayad	An	ra	bavar konad	ya na
heard	and	didn't know (if)	must	that	ra	believe	or not
heavy verb	coordinato r	verb (negative)	modal	demonstrative pronoun	accus. Marker	verb	prep

6

a. خبرنگار برداشتی از حرفهای من کرد در حالی که درست نبود

b. خبرنگار برداشتی که خودش دلش می‌خواست از حرفهای من کرد در حالی که درست نبود

خبرنگار	برداشتی	که	خودش	دلش	می خواست	از	حرف
Khabarnegar	bardashti	ke	Khodash	delash	mikhast	az	harfe

The journalist	an impression	that	Himself	his heart	wanted	from	word (of)
def. noun	indef. Noun	relative marker	reflexive pronoun	possessive adj + noun	verb	prep	noun
من	کرد	در حالی که	درست	نبود			
man	kard	dar hali ke	Dorost	nabood			
me	did	while	Correct	was not			
obj. pronoun	light verb	while	Adjective	verb (negative)			

c. خبرنگار گزارشی از حرفهای من نوشت و آن را در روزنامه چاپ کرد

d. خبرنگار گزارشی که خودش دلش می‌خواست از حرفهای من نوشت و آن را در روزنامه چاپ کرد

خبرنگار	گزارشی	که	خودش	دلش	می خواست	از	حرف
Khabarnegar	gozareshi	ke	Khodash	delash	mikhast	az	harfe
The journalist	a report	that	Himself	his heart	wanted	from	word (of)
def. noun	indef. Noun	relative marker	reflexive pronoun	possessive adj + noun	verb	prep	noun
من	نوشت	در حالی که	درست	نبود			
man	nevesht	dar hali ke	Dorost	nabood			
me	wrote	while	Correct	was not			
obj. pronoun	Heavy verb	subordinator	Adjective	verb(negative)			

7

a. سپیده تحلیلی از مقاله کرد و آن را در کنفرانس ارائه_داد

b. سپیده تحلیلی که به نظر پیچیده می‌آمد از مقاله کرد و آن را در کنفرانس ارائه داد

سپیده	تحلیلی	که	به نظر	پیچیده	می آمد	از	مقاله
Sepideh	tahlili	ke	be nazar	pichideh	mi-amad	az	maghaleh
Sepideh	an analysis	that	to eye	complicated	seemed	from	(the) article
Proper name	indef. Noun	relative marker	pp	adjective	verb	prep	noun
کرد	و	آن	را	در	کنفرانس	ارائه داد	
kard	Va	an	ra	dar	conferans	era'eh dad	
did	And	that	ra	in	conference	presented	
light verb	coordinator	relative marker	accus. Marker	prep	noun	verb	

c. سپیده نقدی از مقاله خواند و در سخنرانیِ خود به آن اشاره کرد

d. سپیده نقدی که به نظر پیچیده می‌آمد از مقاله خواند و در سخنرانیِ خود به آن اشاره کرد

سپیده	نقدی	که	به نظر	پیچیده	می آمد	از	مقاله
Sepideh	Naghdi	ke	be nazar	pichideh	mi-amad	az	maghaleh
Sepideh	a critisism	that	to eye	complicated	seemed	from	(the) article
Proper name	indef. Noun	relative marker	pp	adjective	verb	prep	noun
خواند	و	در	سخنرانی	خود	به	آن	اشاره کرد
khand	Va	dar	sokhanranie	khod	be	an	eshare kard

read	And	in	presentation (of)	himself	to	that	pointed
heavy verb	Coordinato r	prep	noun	reflexive adj	prep	demonstrative pronoun	verb

8

a. سارا انتظاری از من داشت و من نمی‌توانستم آن را برآورده کنم

b. سارا انتظاری که برایم سخت بود از من داشت و من نمی‌توانستم آن را برآورده کنم

سارا	انتظاری	که	برایم	سخت	بود	از	من
Sara	Entezari	ke	barayam	sakht	bood	az	man
Sara	an expectation	that	for me	difficult	was	from	me
Proper name	indef. Noun	relative marker	pp	adjective	verb	prep	obj. pronoun
داشت	و	من	نمی توانستم	آن	را	برآورده کنم	
dasht	Va	man	nemitavanest	an	ra	baravardeh konam	
had	And	I	could not	that	ra	meet	
light verb	coordinator	subj. pronoun	past modal (negative)	demonstrative pronoun	accus. Marker	verb	

c. سارا درخواستی از من داشت و من نمی‌توانستم آن را برآورده‌کنم

d. سارا درخواستی که برایم سخت بود از من داشت و من نمی‌توانستم آن را برآورده‌کنم

سارا	درخواستی	که	برایم	سخت	بود	از	من
Sara	Darkhasti	ke	barayam	sakht	bood	az	man
Sara	a request	that	for me	difficult	was	from	me
Proper name	indef. Noun	relative marker	pp	adjective	verb	prep	obj. pronoun
داشت	و	من	نمی توانستم	آن	را	برآورده کنم	
dasht	Va	man	nemitavanestam	an	ra	baravardeh konam	
had	And	I	couldn't	that	ra	meet	
heavy verb	Coordinato r	subj. pronoun	past modal (negative)	demonstrative pronoun	accus. Marker	verb	

9

a. همکارم سهمی از شرکت برد در حالی که من بیشتر از او برای شرکت زحمت کشیده بودم

b. همکارم سهمی که بسیار ناعادلانه بود از شرکت برد در حالی که من بیشتر از او برای شرکت زحمت کشیده بودم

همکارم	سهمی	که	بسیار	ناعادلانه	بود	از	شرکت	
Hamkaram	sahmi	ke	besyar	na-adelane h	bood	az	sherkat	
My colleague	a share	that	very	unfair	was	from	(the) compan y	
Possessive adj + noun	indef. Noun	relative marker	quantifier	adjectiv e	verb	prep	noun	
برد	در حالی که	من	بیشتر	از	او	برای	شرکت	زحمت کشیده بودم
bord	dar hali ke	man	bishtar	az	oo	baray e	sherkat	zahmat keshideh boodam
won	while	I	more	than	him	for	(the) compan y	had toiled

light verb	subordinator	subj. pronoun	comparative adj	prep	obj. pronoun	prep	noun	verb

c.   همکارم انتقادی از شرکت داشت اما نتوانست هیئت مدیره را متقاعد کند

d.   همکارم انتقادی که بسیار ناعادلانه بود از شرکت داشت اما نتوانست هیئت مدیره را متقاعد کند

همکارم	انتقادی	که	بسیار	ناعادلانه	بود	از	شرکت
Hamkaram	enteghadi	ke	besyar	na-adelaneh	bood	az	sherkat
My colleague	a criticism	that	very	unfair	was	from	(the) company
Possessive adj + noun	indef. Noun	relative marker	quantifier	adj	verb	prep	noun
داشت	اما	نتوانست	هیئت مدیره	را	متقاعد کند		
dasht	amma	natavanest	heiat modireh	ra	motegha'ed konad		
had	But	could not	board of directors	ra	persuade		
heavy verb	Coordinator	past modal (negative)	noun	accus. Marker	verb	prep	noun

10

a.   من خواهشی از مهسا کردم اما او آن را رد کرد

b.   من خواهشی که بسیار ناچیز بود از مهسا کردم اما او آن را رد کرد

من	خواهشی	که	بسیار	ناچیز	بود	از	مهسا
Man	khaheshi	ke	besyar	na-chiz	bood	az	Mahsa
I	a wish	that	very	trifle	was	from	Mahsa
subj. pronoun	indef. Noun	relative marker	quantifier	adj	verb	prep	Proper name
کردم	اما	او	آن	را	رد کرد		
kardam	amma	oo	an	ra	rad kard		
did (i)	But	she	that	ra	rejected		
light verb	coordinator	subj. pronoun	demonstrative pronoun	accus. Marker	verb		

c.   من پولی از مهسا گرفتم چون کیفم را در خانه جا گذاشته بودم

d.   من پولی که بسیار ناچیز بود از مهسا گرفتم چون کیفم را در خانه جا گذاشته بودم

من	پولی	که	بسیار	ناچیز	بود	از	مهسا
man	Pooli	ke	besyar	na-chiz	bood	az	Mahsa
I	a money	that	very	trifle	was	from	Mahsa
subj. pronoun	indef. Noun	relative marker	quantifier	adj	verb	prep	Proper name
گرفتم	چون	کیفم	را	در	خانه	جا گذاشته بودم	
gereftam	Chon	kifam	ra	dar	khaneh	ja gozashte boodam	
got	because	my bag	ra	at	home	had left(i)	
Heavy verb	coordinator	possessive adj + noun	accus. Marker	prep	noun	verb	

11

a.   الهام عطری به لباسش زد که توجهِ همه را جلب کرده بود

b.   الهام عطری که هدیه تولدش بود را به لباسش زد و توجهِ همه را جلب کرده بود

الهام	عطری	که	هدیه	تولدش	بود	را	به
Elham	atri	ke	hedyeye	tavallodash	bood	ra	be
Elham	a perfume	that	gift (of)	her birthday	was	ra	to
Proper name	indef. Noun	relative marker	noun	possessive adj + noun	verb	accus. Marker	prep
لباسش	زد	و	توجه	همه	را	جلب کرد	
lebasash	zad	va	tavajjohe	hame	ra	jalb kard	
her dress	hit	and	attention (of)	all	ra	drew	
possessive adj + noun	light verb	coordinator	noun	noun	accus. Marker	verb	

c. الهام سنجاقی به لباسش آویخت که خیلی با لباسش هماهنگ بود

d. الهام سنجاقی که هدیه تولدش بود را به لباسش آویخت که خیلی با لباسش هماهنگ بود

الهام	سنجاقی	که	هدیه	تولدش	بود	را	به
Elham	sanjaghi	ke	hedyeye	tavallodash	bood	ra	be
Elham	a clips	that	gift (of)	her birthday	was	ra	to
Proper name	indef. Noun	relative marker	noun	possessive adj + noun	verb	accus. Marker	prep
لباسش	آویخت	و	توجه	همه	را	جلب کرد	
lebasash	avikht	va	tavajjohe	hame	ra	jalb kard	
her dress	hang	and	attention (of)	all	ra	drew	
possessive adj + noun	heavy verb	coordinator	noun	noun	accus. Marker	verb	

12

a. محسن اتهامی به من زد اما نتوانست آن را اثبات کند

b. محسن اتهامی که اصلا انتظارش را نداشتم به من زد اما نتوانست آن را اثبات کند

محسن	اتهامی	که	اصلا	انتظارش	را	نداشتم	به
Mohsen	ettehami	ke	aslan	entezarash	ra	nadashtam	be
Mohsen	an accusation	that	at all	expectation (of that)	ra	didn't (i)	to
Proper name	indef. Noun	relative marker	quantifier	noun	accus. Marker	verb (negative)	prep
من	زد	اما	نتوانست	آن	را	ثابت کند	
Man	zad	amma	natavanest	an	ra	sabet konad	
Me	hit	but	could not	that	ra	prove	
obj. pronoun	light verb	coordinator	past modal (negative)	demonstrative pronoun	accus. Marker	verb	

c. محسن رازی به من گفت و حسابی مرا در فکر فرو برد

d. محسن رازی که انتظارش را نداشتم به من گفت و حسابی مرا در فکر فرو برد

محسن	رازی	که	اصلا	انتظارش	را	نداشتم	به
Mohsen	razi	ke	aslan	entezarash	ra	nadashtam	be
Mohsen	a secret	that	at all	expectation (of that)	ra	didn't have(i)	to
Proper name	indef. Noun	relative marker	quantifie r	noun	accus. Marker	verb(negative)	prep
من	گفت	و	حسابی	مرا	در	فکر	فرو برد

Man	goft	va	hesabi	mara	dar	fekr	foroo-bord
Me	said	and	so much	me- ra	in	thought	Drowned
obj. pronoun	heavy verb	coordinator	quantifier	obj. pronoun + accus. Marker	prep	noun	verb

13

a. رضا لبخندی به من زد و من هرگز آن روز را از یاد نمی‌برم

b. رضا لبخندی که سرشار از آرامش بود به من زد و من هرگز آن روز را از یاد نمی‌برم

رضا	لبخندی	که	سرشار	از	آرامش	بود	به
Reza	labkhandi	ke	sarshar	az	aramesh	bood	be
Reza	a smile	that	full	of	comfort	was	to
Proper name	indef. Noun	coordinator	adj	prep	noun	verb	prep
من	زد	و	من	هرگز	آن	را	فراموش نمی کنم
Man	zad	va	man	hargez	an	ra	faramoosh nemikonam
Me	hit	and	I	never	that	ra	forget
obj. pronoun	light verb	coordinator	subj. pronoun	adverb	demonstrative pronoun	accus. Marker	verb

c. رضا باغی به من فروخت و از این شهر رفت

d. رضا باغی که سرشار از آرامش بود به من فروخت و از این شهر رفت

رضا	باغی	که	سرشار	از	آرامش	بود	به
Reza	baghi	ke	sarshar	az	aramesh	bood	be
Reza	a garden	that	full	of	comfort	was	to
Proper name	indef. Noun	relative marker	adj	prep	noun	verb	prep
من	فروخت	و	از	این	شهر	رفت	
Man	forookht	va	az	in	shahr	raft	
Me	sold	and	from	this	city	went	
obj. pronoun	heavy verb	coordinator	prep	demonstrative pronoun	noun	verb	

14

a. مژگان تهمتی به من زد و باعث شد با او قطع رابطه کنم

b. مژگان تهمتی که برایم باورنکردنی بود به من زد و باعث شد با او قطع رابطه کنم

مژگان	تهمتی	که	برایم	باورنکردنی	بود	به	من
Mojgan	tohmati	ke	barayam	bavar-nakardani	bood	be	man
Mojgan	an insult	that	for me	unbelievable	was	to	me
Proper name	indef. Noun	relative marker	pp	adj	verb	prep	obj pronoun
زد	و	باعث شد	با	او	قطع رابطه	کنم	
Zad	va	ba'es shod	ba	oo	ghat'e rabeteh	konam	
Hit	and	caused	with	her	cut-relation	do (i)	
light verb	coodinator	verb	prep	obj pronoun	noun	verb	

c. مژگان هدیه‌ای به من داد و من حسابی ذوقزده شدم

d. مژگان هدیه‌ای که برایم باورنکردنی بود به من داد و من حسابی ذوقزده شدم

مژگان	هدیه ای	که	برایم	باورنکردنی	بود	به	من
Mojgan	hedyei	ke	barayam	bavar-nakardani	bood	be	man
Mojgan	a gift	that	for me	unbelievable	was	to	me
Proper name	indef. Noun	relative marker	pp	adj	verb	prep	obj pronoun
داد	و	من	حسابی	ذوق زده	شدم		
Dad	va	man	hesabi	zogh-zadeh	shodam		
gave	and	I	so much	excited	got		
heavy verb	coodinator	subj. pronoun	quantifier	adj	verb		

15

a. همسرم ضربه‌ای به من زد و زندگی مرا از این رو به آن رو کرد

b. همسرم ضربه ای که انتظار نداشتم به من زد و زندگی مرا از این رو به آن رو کرد

همسرم	ضربه ای	که	اصلا	انتظار	نداشتم	به	زندگیم
hamsaram	zarbei	ke	aslan	entezar	nadashtam	be	zendegiam
My spouse	a damage	that	at all	expect	didn't have (i)	to	my life
possessive adj + noun	indef. Noun	relative marker	quantifier	noun	verb (negative)	prep	possessive adj + noun
زد	و	زندگی	مرا	از	این	رو	به
zad	va	zendegie	mara	az	in	roo	be
hit	and	life (of)	me- ra	from	this	face	to
light verb	coodinator	noun	obj pronoun + accus. Marker	prep	demonstrative adj	noun	prep
آن	رو	کرد					
an	roo	kard					
that	face	did					
demons. Pronoun	noun	verb					

c. همسرم شادی به زندگی من آورد و زندگی مرا از این رو به آن رو کرد

d. همسرم شادی که انتظار نداشتم به زندگی من آورد و زندگی مرا از این رو به آن رو کرد

همسرم	شادی	که	اصلا	انتظار	نداشتم	به	زندگیم
Hamsaram	shadi	ke	aslan	entezar	nadashtam	be	zendegiam
My spouse	a happiness	that	at all	expect	didn't have (i)	to	my life
possessive adj + noun	indef. Noun	relative marker	quantifier	noun	verb(negative)	prep	possessive adj + noun
آورد	و	زندگی	مرا	از	این	رو	به
avard	va	zendegie	mara	az	in	roo	be
brought	and	life (of)	me- ra	from	this	face	to
heavy verb	coodinator	noun	obj pronoun + accus. Marker	prep	demons. Adj	noun	prep
آن	رو	کرد					
aan	roo	kard					
that	face	did					

demons. Pronoun	noun	verb					

16

a. فرزاد حرفی به من زد و از اتاق خارج شد

b. فرزاد حرفی که بسیار جالب بود به من زد و از اتاق خارج شد

فرزاد	حرفی	که	بسیار	جالب	بود	به	من
Farzad	harfi	ke	besyar	jaleb	bood	be	man
Farzad	a word	that	very	interesting	was	to	me
Proper name	indef. Noun	relative marker	quantifier	adj	verb	prep	obj pronoun
زد	و	از	اتاق	خارج شد			
Zad	va	az	otagh	kharej shod			
Hit	and	from	(the) room	went out			
light verb	coodinator	prep	noun	verb			

c. فرزاد کتابی به من داد اما هنوز فرصت نکردم آن را بخوانم

d. فرزاد کتابی که بسیار جالب بود به من داد اما هنوز فرصت نکردم آن را بخوانم

فرزاد	کتابی	که	بسیار	جالب	بود	به	من
Farzad	ketabi	ke	besyar	jaleb	bood	be	man
Farzad	a book	that	very	interesting	was	to	me
Proper name	indef. Noun	relative marker	quantifier	adj	verb	prep	obj pronoun
داد	اما	هنوز	فرصت	نکردم	آن	را	بخوانم
Dad	amma	hanooz	forsat	nakardam	an	ra	bekhanam
Gave	but	still	chance	didn't have	that	ra	read
heavy verb	coodinator	adverb	noun	verb	demons. Pronoun	accus. Marker	verb

17

a. معلم توصیه‌ای به من کرد و من سعی کردم آن را به کار ببندم

b. معلم توصیه‌ای که بسیار مفید بود به من کرد و من سعی کردم آن را به کار ببندم

معلم	توصیه ای	که	بسیار	مفید	بود	به	من
moallemam	tosieii	ke	besyar	mofid	bood	be	man
My teacher	a recommendation	that	very	useful	was	to	me
possessive adj + noun	indef. Noun	relative marker	quantifier	adj	verb	prep	obj pronoun
کرد	و	من	سعی کردم	آن	را	به کار ببندم	
kard	va	man	say kardam	an	ra	be kar bebandam	
did	an	I	tried	that	ra	put into practice	
light verb	coodinator	subj. pronoun	verb	demonstrative pronoun	accus. Marker	verb	

c. معلم درسی به من آموخت و من هرگز آن را فراموش نمی‌کنم

d. معلم درسی که بسیار مفید بود به من آموخت و من هرگز آن را فراموش نمی‌کنم

معلمم	درسی	که	بسیار	مفید	بود	به	من
moallemam	darsi	ke	besyar	mofid	bood	be	man
My teacher	a lesson	that	very	useful	was	to	me
possessive adj + noun	indef. Noun	relative marker	quantifier	adj	verb	prep	obj pronoun
آموخت	و	من	آن	را	هرگز	فراموش نمی کنم	
amookht	va	man	an	ra	hargez	faramoosh nemikonam	
taught	and	I	that	ra	never	forget	
heavy verb	coodinato r	subj. pronoun	demons. Pronoun	accus. Marker	adverb	verb	

18

a. کوروش نصیحتی به من کرد و من آن را گوش‌دادم

b. کوروش نصیحتی که به نفعم بود به من کرد و من آن را گوش دادم

کوروش	نصیحتی	که	به	نفعم	بود	به	من
Kourosh	nasihati	ke	be	naf'am	bood	be	man
Kourosh	an advice	that	to	my benefit	was	to	me
Proper name	indef. Noun	relative marker	prep	possessive adj + noun	verb	prep	obj pronoun
کرد	و	من	آن	را	گوش دادم		
Kard	va	man	an	ra	goosh dadam		
Did	and	I	that	ra	listened		
light verb	coodinator	subj. pronoun	demons. Pronoun	accus. Marker	verb		

c. کوروش نکته‌ای به من فهماند و من تازه حقیقت را فهمیدم

d. کوروش نکته‌ای که به نفعم بود به من فهماند و من تازه حقیقت را فهمیدم

کوروش	نکته ای	که	به	نفعم	بود	به	من
Kourosh	noktei	ke	be	naf'am	bood	be	man
Kourosh	a point	that	to	my interest	was	to	me
Proper name	indef. Noun	relative marker	prep	possessive adj + noun	verb	prep	obj pronoun
فهماند	و	من	تازه	حقیقت	را	فهمیدم	
fahmand	va	man	taze	haghighat	ra	fahmidam	
made across	and	I	just	(the) truth	ra	understood	
heavy verb	coodinator	subj. pronoun	adverb	noun	accus. Marker	verb	

19

a. شبنم پیشنهادی به من کرد و من آن را قبول کردم

b. شبنم پیشنهادی که به نظر باارزش می‌آمد به من کرد و من آن را قبول کردم

شبنم	پیشنهادی	که	به نظر	با ارزش	می آمد	به	من
Shabnam	pishnahadi	ke	be nazar	ba-arzesh	mi-amad	be	man
Shabnam	a suggestion	that	to eye	valuable	seemed	to	me
Proper name	indef. Noun	relative marker	pp	adj	verb	prep	obj pronoun
کرد	و	من	آن	را	قبول کردم		

Kard	va	man	an	ra	ghabool kardam		
Did	and	I	that	ra	accepted		
light verb	coodinator	subj. pronoun	demons. Pronoun	accus. Marker	verb		

c. شبنم تابلویی به من بخشید و من آن را به دیوارِ اتاقِ نشیمن زدم

d. شبنم تابلویی که به نظر باارزش می‌آمد به من بخشید و من آن را به دیوارِ اتاقِ نشیمن زدم

شبنم	تابلویی	که	به نظر	با ارزش	می آمد	به	من
Shabnam	tablo'i	ke	be nazar	ba-arzesh	mi-amad	be	man
Shabnam	a picture	that	to-eye	valuable	seemed	to	me
Proper name	indef. Noun	relative marker	pp	adj	verb	prep	obj pronoun
بخشید	و	من	آن	را	به	دیوار	زدم
bakhshid	va	man	an	ra	be	divar	zadam
spared	and	I	that	ra	to	(the) wall	attached (i)
heavy verb	coodinator	subj. pronoun	demons. Pronoun	accus. Marker	prep	noun	verb

20

a. بهنام راهنمایی به من کرد و باعث شد بتوانم تصمیمِ نهایی را بگیرم

b. بهنام راهنمایی که دنبالش بودم را به من کرد و باعث شد بتوانم تصمیمِ نهایی را بگیرم

بهنام	راهنمایی	که	مدت ها	دنبالش	بودم	را	به
Behnam	rahnamayee	ke	moddat-ha	donbalash	boodam	ra	be
Behnam	a guidance	that	(for) long time	looking for	was (i)	ra	to
Proper name	indef. Noun	relative marker	adverb	noun	verb	accus. Marker	prep
من	کرد	و	باعث شد	بتوانم	تصمیم	را	بگیرم
Man	kard	va	ba'es shod	betavanam	tasmimam	ra	begiram
Me	did	and	caused (that)	Can (i)	my decision	ra	make (i)
obj. pronoun	light verb	coordinator	verb	modal	noun	accus. Marker	verb

c. بهنام مقاله‌ای به من داد و حسابی کارم را جلو انداخت

d. بهنام مقاله‌ای که دنبالش بودم را به من داد و حسابی کارم را جلو انداخت

بهنام	مقاله ای	که	مدت ها	دنبالش	بودم	را	به
Behnam	maghale'ii	ke	moddat-ha	donbalash	boodam	ra	be
Behnam	an aricle	that	(for) long time	looking for	was (i)	ra	to
Proper name	indef. Noun	relative marker	adverb	noun	verb	accus. Marker	prep
من	داد	و	حسابی	کارم	را	جلو انداخت	
Man	dad	va	hesabi	karam	ra	jolo andakht	
Me	gave	and	so much	my work	ra	progressed	
obj. pronoun	heavy verb	coordinator	quantifier	possessive adj + noun	accus. Marker	verb	

21

a. همسایه‌ام تذکری به دوستم داد و از او خواست کمتر سیگار بکشد

b. همسایه‌ام تذکری که کاملا به‌موقع بود به دوستم داد و از او خواست کمتر سیگار بکشد

همسایه ام	تذکری	که	کاملا	به موقع	بود	به	دوستم
hamsaye'am	tazakkori	ke	kamelan	be moghe'	bood	be	doostam
My neighbor	a point	that	completely	timely	was	to	my friend
possessive adj + noun	indef. Noun	relative marker	adverb	adj	verb	prep	possessive adj + noun
داد	و	از	او	خواست	کمتر	سیگار بکشد	
dad	va	az	oo	khast	kamtar	sigar bekeshad	
gave	and	from	him/her	wanted	less	smoke	
light verb	coodinator	prep	obj pronoun + accus. Marker	verb	adj	verb	

c. همسایه‌ام دوایی به دوستم رساند و جانِ او را نجات داد
d. همسایه‌ام دوایی که کاملا به‌موقع بود به دوستم رساند و جان او را نجات داد

همسایه ام	دوایی	که	کاملا	به موقع	بود	به	دوستم
Hamsaye'am	davayee	ke	kamelan	be moghe	bood	be	doostam
My neighbord	a medication	that	completely	timely	was	to	my friend
possessive adj + noun	indef. Noun	relative marker	adverb	adj	verb	prep	possessive adj + noun
رساند	و	جان	او	را	نجات داد		
resand	va	jane	oo	ra	nejat dad		
fetched	and	life (of)	him/her	ra	saved		
heavy verb	coodinator	noun	obj pronoun + accus. Marker	accus. Marker	verb		

22

a. نیما سفارشی به من کرد و از من خواست که مراقبِ خواهرش باشم
b. نیما سفارشی که بسیار حیاتی بود به من کرد و از من خواست که مراقبِ خواهرش باشم

نیما	سفارشی	که	بسیار	حیاتی	بود	به	من
Nima	sefareshi	ke	besyar	hayati	bood	be	man
Nima	a request	that	very	life-saving	was	to	me
Proper name	indef. Noun	relative marker	quantifier	adj	verb	prep	obj pronoun
کرد	و	از	من	خواست	که	مراقب	خواهرش
Kard	va	az	man	khast	ke	moraghebe	khaharash
Did	and	from	me	wanted	that	looking after	his sister
light verb	coodinator	prep	obj pronoun + accus. Marker	verb	relative marker	adj	possessive adj + noun
باشم							
Basham							
am (i)							

Verb							

c. نیما امانتی به من سپرد و از من خواست تا آن را به دستِ خواهرش برسانم

d. نیما امانتی که بسیار حیاتی بود به من سپرد و از من خواست تا آن را به دستِ خواهرش برسانم

نیما	امانتی	که	بسیار	حیاتی	بود	به	من
Nima	amanati	ke	besyar	hayati	bood	be	man
Nima	a borrowing	that	very	life-saving	was	to	me
Proper name	indef. Noun	relative marker	quantifier	adj	verb	prep	obj pronoun
سپرد	و	از	من	خواست	تا	آن	را
sepord	va	az	man	khast	ta	an	ra
Gave	and	from	me	wanted	so that	that	ra
heavy verb	coodinator	prep	obj pronoun	verb	subordinator	demons. Pronoun	accus. Marker
به	دست	خواهرش	برسانم				
Be	daste	khaharash	beresanam				
To	hand (of)	his sister	take (i)				
Prep	noun	possessive adj + noun	verb				

23

a. آدمرباها هشداری به من دادند و از من خواستند تا پول را هرچه زودتر آماده کنم وگرنه پسرم را می‌کشند

b. آدمرباها هشداری که بسیار جدی بود به من دادند و از من خواستند تا پول را هرچه زودتر آماده کنم وگرنه پسرم را می‌کشند

آدم رباها	هشداری	که	بسیار	جدی	بود	به	من
adam-roba-ha	hoshdari	ke	besyar	jeddi	bood	be	man
The kidnappers	a warning	that	very	serious	was	to	me
Noun	indef. Noun	relative marker	quantifier	adj	verb	prep	obj pronoun
دادند	و	از	من	خواستند	تا	پول	را
dadand	va	az	man	khastand	ta	pool	ra
gave	and	from	me	wanted	so that	(the) money	ra
light verb	coodinator	prep	obj. pronoun	verb	subordinator	noun	accus. Marker
هر چه	زودتر	آماده کنم	وگرنه	پسرم	را	می کشند	
har che	zoodtar	amadeh konam	vagarna	pesaram	ra	mikoshand	
whatever	sooner	prepare(i)	otherwise	my son	ra	kill(they)	
quantifier	adverb	verb	coordinator	possessive adj + noun	accus. Marker	verb	

c. آدمرباها اخطاری به من فرستادند و از من خواستند تا پول را هرچه زودتر آماده کنم وگرنه پسرم را می‌کشند

d. آدمرباها اخطاری که بسیار جدی بود به من فرستادند و از من خواستند تا پول را هرچه زودتر آماده کنم وگرنه پسرم را می‌کشند

آدم رباها	اخطاری	که	بسیار	جدی	بود	به	من
Adam-roba-ha	ekhtari	ke	besyar	jeddi	bood	be	man
The kidnappers	a warning	that	very	serious	was	to	me

noun	indef. Noun	relative marker	quantifier	adj	verb	prep	obj pronoun
فرستادند	و	از	من	خواستند	تا	پول	را
ferestadand	va	az	man	khastand	ta	pool	ra
sent (they)	and	from	me	wanted	so that	(the) money	ra
heavy verb	coodinato r	prep	obj pronoun	verb	subordinato r	noun	accus. Marker
هر چه	زودتر	آماده کنم	وگرنه	پسرم	را	می کشند	
harche	zoodtar	amadeh konam	vagarna	pesaram	ra	mikoshan d	
whatever	sooner	prepare	otherwise	my son	ra	kill (they)	
quantifier	adverb	verb	coordinato r	possessive adj + noun	accus. Marker	verb	

24

a. فرهاد قولی به من داد و دیگر خیالم راحت شد

b. فرهاد قولی که بسیار امیدبخش بود به من داد و دیگر خیالم راحت شد

فرهاد	قولی	که	بسیار	امیدبخش	بود	به	من
Farhad	gholi	ke	besyar	omid-bakhsh	bood	be	man
Farhad	a promise	that	very	promising	was	to	me
Proper name	indef. Noun	relative marker	quantifier	adj	verb	prep	obj pronoun
داد	و	دیگر	خیالم	راحت شد			
Dad	va	digar	khialam	rahat shod			
Gave	and	from that time	my soul	got relaxed			
light verb	coodinator	adverb	possessive adj + noun	verb			

c. فرهاد خبری به من داد و مرا از نگرانی درآورد

d. فرهاد خبری که بسیار امیدبخش بود به من داد و مرا از نگرانی درآورد

فرهاد	خبری	که	بسیار	امیدبخش	بود	به	من
Farhad	khabari	ke	besyar	omid-bakhsh	bood	be	man
Farhad	a (piece of) news	that	very	promising	was	to	me
Proper name	indef. Noun	relative marker	quantifier	adj	verb	prep	obj pronoun
داد	و	مرا	از	نگرانی	درآورد		
Dad	va	mara	az	negarani	dar-avard		
Gave	and	me-ra	from	worry	took-out		
heavy verb	coodinator	obj pronoun + accus. Marker	prep	noun	verb		

25

a. میترا دروغی به من گفت اما بعد دستش رو شد

b. میترا دروغی که واقعی به‌نظر می‌رسید به من گفت اما بعد دستش رو شد

میترا	دروغی	که	واقعی	به نظر	می رسید	به	من
Mitra	dorooghi	ke	vaghei	be nazar	miresid	be	man

Mitra	a lie	that	real	to eye	seemed	to	me
Proper name	indef. Noun	relative marker	adj	pp	verb	prep	obj pronoun
گفت	اما	بعد	دستش	رو شد			
Goft	amma	ba'd	dastash	roo shod			
Told	but	then	her hands	got caught			
light verb	coodinator	adverb	noun + possesive adj	verb			

c. میترا عروسکی به من داد و من او را از خودم جدا نمی‌کردم

d. میترا عروسکی که واقعی به‌نظر می‌رسید به من داد و من او را از خودم جدا نمی‌کردم

میترا	عروسکی	که	واقعی	به نظر	می رسید	به	من
Mitra	aroosaki	ke	vaghei	be nazar	miresid	be	man
Mitra	a doll	that	real	to eye	seemed	to	me
Proper name	indef. Noun	relative marker	adj	pp	verb	prep	obj pronoun
داد	و	من	آن	را	از	خودم	جدا نمی کردم
Dad	va	man	an	ra	az	khodam	joda nemikardam
Gave	and	I	that	ra	from	my self	didn't separate
heavy verb	coodinator	subj. pronoun	demons. Pronoun	accus. Marker	prep	demons. Pronoun	verb

26

a. نیلوفر پیشرفتی در مدرسه کرد و از معلمش جایزه گرفت

b. نیلوفر پیشرفتی که قولش را داده بود در مدرسه کرد و از معلمش جایزه گرفت

نیلوفر	پیشرفتی	که	قولش	را	داده بود	در	مدرسه
Niloofar	pishrafti	ke	gholash	ra	dadeh bood	dar	madreseh
Niloofar	a progress	that	promise (of that)	ra	had given	at	school
Proper name	indef. Noun	relative marker	noun+possesive adj	accus. Marker	verb	prep	noun
کرد	و	از	معلمش	جایزه	گرفت		
Kard	va	az	moallemash	jayezeh	gereft		
Did	and	from	her teacher	prize	got		
light verb	coodinator	from	noun+possesive adj	noun	verb		

c. نیلوفر دکلمه‌ای در مدرسه خواند و جایزه‌ی بهترین دکلمه‌ی منطقه را گرفت

d. نیلوفر دکلمه‌ای که قولش را داده بود در مدرسه خواند و جایزه‌ی بهترین دکلمه‌ی منطقه را گرفت

نیلوفر	دکلمه ای	که	قولش	را	داده بود	در	مدرسه
Niloofar	deklamei	ke	gholash	ra	dadeh bood	dar	madrese
Niloofar	a composition	that	promise (of that)	ra	had given	at	school
Proper name	indef. Noun	relative marker	noun+demons. Adj	accus. Marker	verb	prep	noun
خواند	و	جایزه	بهترین	دکلمه	منطقه	را	گرفت
khand	va	jayezeye	behtarin	deklameye	mantagheh	ra	gereft
Read	and	prize (of)	(the) best	composition (of)	(the) region	ra	got

Heavy Verb	coodinator	noun	adj	noun	noun	accus. Marker	verb

27

a. مجید لطفی به من کرد و من همیشه به او مدیونم

b. مجید لطفی که دور از ذهن بود به من کرد و من همیشه به او مدیونم

مجید	لطفی	که	دور	از	ذهن	بود	به
Majid	lotfi	ke	door	az	zehn	bood	be
Majid	a favor	that	far	from	mind	was	to
Proper name	indef. Noun	relative marker	adj	prep	noun	verb	prep
من	کرد	و	من	همیشه	به	او	مدیونم
man	kard	va	man	hamishe	be	oo	madioonam
Me	did	and	I	always	to	him	owe (i)
obj. pronoun	light verb	coordinator	subj. pronoun	adverb	prep	obj. pronoun	prep

c. مجید ناسزایی به من گفت و من تا عمر دارم او را نمی‌بخشم

d. مجید ناسزایی که دور از ذهن بود به من گفت و من تا عمر دارم او را نمی‌بخشم

مجید	ناسزایی	که	دور	از	ذهن	بود	به
Majid	nasezayee	ke	door	az	zehn	bood	be
Majid	a swearword	that	far	from	mind	was	to
Proper name	indef. Noun	relative marker	adj	prep	noun	verb	prep
من	گفت	و	من	تا	عمر دارم	فراموش نمی کنم	
Man	goft	va	man	ta	omr daram	faramoosh nemikonam	
Me	said	and	I	till	alive am	don't forget	
obj. pronoun	Heavy verb	coordinator	subj. pronoun	prep	verb	verb	

28

a. استاد صحبتی با شاگردش کرد و او را متقاعد کرد که تصمیمش را عوض کند

b. استاد صحبتی که بسیار اثرگذار بود با شاگردش کرد و او را متقاعد کرد که تصمیمش را عوض کند

استاد	صحبتی	که	بسیار	اثرگذار	بود	با	شاگردش	
ostad	Sohbati	ke	besyar	asargozar	bood	ba	shagerdash	
(The) professor	a word	that	very	impressive	was	to	his/her student	
Noun	indef. Noun	relative marker	quantifier	adj	verb	prep	noun+possessive adj	
کرد	و	او	را	متقاعد کرد	که	تصمیمش	را	عوض کند
kard	Va	oo	ra	motegha'ed kard	ke	tasmimash	ra	avaz konad
did	And	him/her	ra	persuaded	that	his/her decision	ra	change
light verb	Coodinator	subj. pronoun	accus. Marker	verb	relative marker	noun + possessive pronoun	accus. Marker	verb

c. استاد قطعه‌ای با شاگردش نواخت و جمعیت حسابی تشویقشان کردند

d. استاد قطعه‌ای که بسیار اثرگذار بود با شاگردش نواخت و جمعیت حسابی تشویقشان کردند

استاد	قطعه ای	که	بسیار	اثرگذار	بود	با	شاگردش
Ostad	ghat'ei	ke	besyar	asar-gozar	bood	ba	shagerdash
(the) professor	a piece	that	very	impressive	was	with	his/her student
Noun	indef. Noun	relative marker	quantifie r	adj	verb	prep	noun+possessive adj
نواخت	و	جمعیت	حسابی	تشویقشان کردند			
navakht	Va	jamiat	hesabi	tashvigheshan kardan			
played	and	(the) crowed	so much	clapped (for them)			
heavy verb	Coodinato r	noun	quantifie r	verb			

29

a. سینا قراری با ندا گذاشت اما او سرِ قرار نیامد

b. سینا قراری که انتظارش را می‌کشید با ندا گذاشت اما او سرِ قرار نیامد

سینا	قراری	که	انتظارش	را	می کشید	با	ندا
Sina	gharari	ke	entezarash	ra	mikeshid	ba	Neda
Sina	an appointment	that	expected	ra	had	with	Neda
Proper name	indef. Noun	relative marker	noun+possesive adj	accus. Marker	verb	prep	Proper Name
گذاشت	اما	او	سر	قرار	نیامد		
gozasht	amma	oo	sare	gharar	nayamad		
put	But	she	to	(the) appointment	didn't come		
light verb	coodinator	subj. pronoun	prep	noun	verb		

c. سینا سفری با ندا رفت و به آنها خیلی خوش گذشت

d. سینا سفری که انتظارش را می‌کشید با ندا رفت و به آنها خیلی خوش گذشت

سینا	سفری	که	انتظارش	را	می کشید	با	ندا
Sina	safari	ke	entezarash	ra	mikeshid	ba	Neda
Sina	a travel	that	expected	ra	had	with	Neda
Proper name	indef. Noun	relative marker	noun+possesive adj	accus. Marker	verb	prep	Proper Name
رفت	و	به	آنها	خیلی	خوش گذشت		
raft	Va	be	anha	kheili	khosh gozasht		
went	And	to	them	very much	fun was		
heavy verb	coodinator	prep	demons. Pronoun	quantifier	verb		

30

a. من حدسی در موردِ ستاره زدم ولی خیلی دیر متوجه شدم که حدسم درست نبوده است

b. من حدسی که اشتباه از آب درآمد در موردِ ستاره زدم ولی خیلی دیر متوجه شدم که حدسم درست نبوده است

من	حدسی	که	اشتباه	از آب	درآمد	در مورد	ستاره
Man	hadsi	ke	wrong	az ab	dar-amad	dar morede	Setareh
I	a guess	that	mistake	from water	came out	about	Setareh

Subj. pronoun	indef. Noun	relative marker	adj	pp	verb	prep	Proper Name
زدم	و	خیلی	دیر	متوجه	شدم	که	حدسم
zadam	va	kheili	dir	motevajje h	shodam	ke	hadsam
hit (i)	and	very	late	realize	did	that	my guess
light verb	coodinator	quantifier	adverb	adj	verb	subordinato r	noun+possessive adj
درست	نبوده است						
dorost	naboodeh ast						
correct	was not						
adj	verb						

c. من شایعه‌ای در موردِ ستاره شنیدم و خیلی عصبانی شدم

d. من شایعه‌ای که اشتباه از آب درآمد در موردِ ستاره شنیدم و خیلی عصبانی شدم

من	شایعه ای	که	اشتباه	از آب	درآمد	در مورد	ستاره
man	shaye'ei	ke	eshtebah	az ab	dar-amad	dar morede	Setareh
I	a rumor	that	wrong	from water	came-out	about	Setareh
Subj. pronoun	indef. Noun	relative marker	noun	pp	verb	prep	Proper Name
شنیدم	و	خیلی	عصبانی	شدم			
shenidam	va	kheili	asabani	shodam			
Heard (i)	and	very	angry	was (i)			
heavy verb	coodinator	quantifier	adj	verb			

31

a. پژمان قضاوتی در موردِ من کرد اما متوجهِ اشتباهش شد

b. پژمان قضاوتی که اصلا منصفانه نبود در موردِ من کرد اما متوجهِ اشتباهش شد

پژمان	قضاوتی	که	اصلا	منصفانه	نبود	در مورد	من
Pejman	ghezavati	ke	aslan	monsefaneh	nabood	dar morede	man
Pejman	a judgement	that	at all	fair	was not	about	me
Proper name	indef. Noun	relative marker	quantifier	adj	verb	prep	obj pronoun
کرد	اما	متوجه	اشتباهش	شد			
kard	amma	motevajjehe	eshtebahash	shod			
did	But	realize	his mistake	got			
light verb	coodinator	adj	noun+possesive adj	verb			

c. پژمان خبری در موردِ من شنید اما آن را باور نکرد

d. پژمان خبری که اصلا منصفانه نبود در موردِ من شنید اما آن را باور نکرد

پژمان	خبری	که	اصلا	منصفانه	نبود	در مورد	من
Pejman	khabari	ke	aslan	monsefaneh	nabood	dar morede	man
Pejman	a (piece of) news	that	at all	fair	was not	about	me
Proper	indef. Noun	relative marker	quantifier	adj	verb	prep	obj

name							pronoun
شنید	اما	آن	را	باور نکرد			
shenid	amma	an	ra	bavar nakard			
heard	but	that	ra	didn't believe			
heavy verb	coodinator	demonstrative pronoun	accus. Marker	verb			

32

a. کاوه حسی در موردِ پریسا می‌کرد و من این را از نگاهش می‌خواندم

b. کاوه حسی که برایش عجیب بود در موردِ پریسا می‌کرد و من این را از نگاهش می‌خواندم

کاوه	حسی	که	برایش	عجیب	بود	در مورد	پریسا
Kaveh	Hessi	ke	barayash	ajib	bood	dar morede	Parisa
Kaveh	a feeling	that	for him	weird	was	about	Parisa
Proper name	indef. Noun	relative marker	pp	adj	verb	prep	Proper Name
می کرد	و	من	این	را	از	نگاهش	می خواندم
mi-kard	Va	man	in	ra	az	negahash	mikhandam
did	And	I	this	ra	from	his look	read
light verb	Coodinator	subj. pronoun	demons. Pronoun	accus. Marker	prep	noun+possessive adj	verb

c. کاوه واقعیتی در موردِ پریسا فهمید اما به رویِ خودش نیاورد

d. کاوه واقعیتی که برایش عجیب بود در موردِ پریسا فهمید اما به روی خودش نیاورد

کاوه	واقعیتی	که	برایش	عجیب	بود	در مورد	پریسا
Kaveh	vagheiati	ke	barayash	ajib	bood	dar morede	Parisa
Kaveh	a truth	that	for him	weird	was	about	Parisa
Proper name	indef. Noun	relative marker	pp	adj	verb	about	Proper Name
فهمید	اما	به	روی	خودش	نیاورد		
Fahmid	amma	be	rooye	khodash	nayavard		
understood	but	to	face (of)	face	didn't bring		
heavy verb	coodinator	prep	noun	noun	verb		

33

a. حامد استدلالی در موردِ مسئله کرد ولی نتوانست استادش را مجاب کند

b. حامد استدلالی که درست به نظر می‌رسید در مورد مسئله کرد ولی نتوانست استادش را مجاب کند

حامد	استدلالی	که	درست	به نظر	می رسید	در مورد	مسئله
Hamed	estedlali	ke	dorost	be nazar	mi-resid	dar morede	mas'aleh
hamed	a reasoning	that	right	to eye	seemed	about	(the) matter
Proper name	indef. Noun	relative marker	adj	pp	verb	about	noun
کرد	ولی	نتوانست	استادش	را	مجاب کند		
kard	Vali	natavanest	ostadash	ra	mojab konad		
did	But	could not	his professor	ra	convince		

light verb	coodinator	modal (negative)	noun+possesive adj	accus. Marker	verb		

c. حامد چاره‌ای در موردِ مسئله اندیشید و اصرار داشت آن را عملی کند

d. حامد چاره‌ای که درست به نظر می‌رسید در موردِ مسئله اندیشید و اصرار داشت آن را عملی کند

حامد	چاره ای	که	درست	به نظر	می رسید	در مورد	مسئله
Hamed	charei	ke	dorost	be nazar	miresid	dar morede	mas'aleh
Hamed	a solution	that	right	to eye	seemed	about	(the) matter
Proper name	indef. Noun	relative marker	adj	pp	verb	prep	noun
اندیشید	و	اصرار داشت	آن	را	عملی کند		
andishid	Va	esrar dasht	an	ra	amali konad		
thought	And	insisted	that	ra	execute		
heavy verb	coodinator	verb	relative marker	accus. Marker	verb		

34

a. حسین اشتباهی در موردِ همسرش کرد و آن اشتباه باعثِ جدايیِ آنها شد

b. حسین اشتباهی که بسیار بزرگ بود در موردِ همسرش کرد و آن اشتباه باعثِ جدايیِ آنها شد

حسین	اشتباهی	که	بسیار	بزرگ	بود	در مورد	همسرش
Hossein	eshtebahi	ke	besyar	bozorg	bood	dar morede	hamsarash
Hossein	a mistake	that	very	big	was	about	his wife
Proper name	indef. Noun	relative marker	quantifier	adj	verb	about	noun+possessive adj
کرد	و	آن	اشتباه	باعث	جدایی	آنها	شد
kard	Va	an	eshtebah	ba'ese	jodayie	an-ha	shod
did	And	that	mistake	cause (of)	separation (of)	them	was
light verb	Coodinator	demons. Adj	noun	prep	noun	demons. Pronoun	verb

c. حسین نکته‌ای در موردِ همسرش فهمید و باعث شد احساسش در موردِ او تغییر کند

d. حسین نکته‌ای که بسیار بزرگ بود در موردِ همسرس فهمید و باعث شد احساسش در موردِ او تغییر کند

حسین	نکته ای	که	بسیار	بزرگ	بود	در مورد	همسرش
Hossein	nokte'i	ke	besyar	bozorg	bood	dar morede	hamsarash
Hossein	a point	that	very	big	was	about	his wife
Proper name	indef. Noun	relative marker	quantifier	adj	verb	prep	noun+possessive adj
فهمید	و	باعث شد	احساسش	در مورد	او	تغییر کند	
fahmid	Va	ba'es shod	ehsasash	dar morede	oo	taghir konad	
understood	And	caused	his feeling	about	her	change	
heavy verb	Coodinator	verb	noun+possesive adj	prep	obj. pronoun	verb	

35

a. نازنین تصمیمی در موردِ خودش گرفت و همه‌ی تلاشش را به کار بست تا آن را اجرا کند

b. نازنین تصمیمی که بسیار تلخ بود در موردِ خودش گرفت و همه‌ی تلاشش را به کار بست تا آن را اجرا کند

نازنین	تصمیمی	که	بسیار	تلخ	بود	در مورد	خودش
Nazanin	tasmimi	ke	besyar	talkh	bood	dar morede	khodash
Nazanin	a decision	that	very	bitter	was	about	herself
Proper name	indef. Noun	relative marker	quantifier	adj	verb	prep	reflexive pronoun
گرفت	و	همه	تلاشش	را	کرد	تا	آن
gereft	va	hameye	talashash	ra	kard	ta	an
got	and	all (of)	her effeort	ra	did	so that	that
light verb	coodinator	quantifier	noun+possesive adj	accus. Marker	verb	subordinato r	demons. Pronoun
را	اجرا کند						
ra	ejra konad						
ra	accomplis h						
accus. Marker	verb						

c. نازنین واقعیتی در موردِ خودش فهمید و فکرش حسابی مشغول شد
d. نازنین واقعیتی که بسیار تلخ بود در موردِ خودش فهمید و فکرش حسابی مشغول شد

نازنین	واقعیتی	که	بسیار	تلخ	بود	در مورد	خودش
Nazanin	vagheiati	ke	besyar	talkh	bood	dar morede	khodash
Nazanin	a truth	that	very	bitter	was	about	her self
Proper name	indef. Noun	relative marker	quantifie r	adj	verb	prep	reflexive pronoun
فهمید	و	فکرش	حسابی	مشغول	شد		
fahmid	va	fekrash	hesabi	mashghoo l	shod		
Understoo d	and	her mind	so much	busy	got		
heavy verb	coodinato r	noun+possessive adj	quantifie r	adj	verb		

36

a. مهدی اعترافی در دادگاه کرد و به حبسِ ابد محکوم_شد
b. مهدی اعترافی که به ضررِ خودش تمام شد در دادگاه کرد و به حبسِ ابد محکوم شد

مهدی	اعترافی	که	به ضرر	خودش	تمام شد	در	دادگاه
Mehdi	E'terafi	ke	be zarare	khodash	tamam shod	dar	dadgah
Mehdi	a confession	that	against	himself	was ended	at	(the) court
Proper name	indef. Noun	relative marker	adj	reflexive pronoun	verb	prep	noun
کرد	و	به	حبس	ابد	محکوم	شد	
kard	va	be	habs-e	abad	mahkoom	shod	
did	and	to	imprisonment (of)	for ever	condemned	got	
light verb	coodinator	prep	noun	adj	pre-verbal adj	verb	

c. مهدی شهادت نامه‌ای در دادگاه نوشت و به حبسِ ابد محکوم شد
d. مهدی شهادت نامه‌ای که به ضرر خودش تمام شد در دادگاه نوشت و به حبسِ ابد محکوم شد

مهدی	شهادت نامه ای	که	به ضرر	خودش	تمام شد	در	دادگاه
Mehdi	shahadat-namei	ke	be zarare	khodash	tamam shod	dar	dadgah
Mehdi	a testimony	that	against	himself	was ended	at	(the) court
Proper name	indef. Noun	relative marker	adj	reflexive pronoun	verb	prep	noun
نوشت	و	به	حبس	ابد	محکوم	شد	
nevesht	va	be	habse	abad	mahkoom	shod	
wrote	and	to	imprisonment (of)	for ever	condemned	got	
heavy verb	coodinator	prep	noun	adj	pre-verbal adj	verb	
