# Supplementary material for: Dependency Resolution Difficulty Increases with Distance in Persian Separable Complex Predicates: Evidence for Expectation and Memory-Based Accounts
Source: Front Psychol. 2016 Mar 30;7:403. doi: 10.3389/fpsyg.2016.00403 (PMC4812816; doi:10.3389/fpsyg.2016.00403)
Supplement: Supplementary file 1 [file DataSheet1.zip › SafaviEtAl2016DataCode/items_fillers/Expt2.glossing.pdf]

Items for Persian Experiment II

1

a. مریم آرزویی برای من کرد که خیلی زود برآورده شد

b. مریم آرزویی برای خواهرِ دوستِ من کرد که خیلی زود برآورده شد

مریم	آرزویی	برای	خواهر	دوست	من
Maryam	arezooyee	baraye	khahare	doost-e	man
Maryam	a wish	for	sister (of)	friend (of)	me
Proper name	indef. Noun	prep	noun	noun	obj. pronoun
کرد	و	خیلی	زود	برآورده شد	
kard	va	kheili	zood	bar-avardeh shod	
did	and	very	soon	came true	
light verb	coordinator	quantifier	adverb	verb	

c. مریم غذایی برای من پخت که خیلی خوشمزه بود

d. مریم غذایی برای خواهرِ دوستِ من پخت که خیلی خوشمزه بود

مریم	غذایی	برای	خواهر	دوست	من	پخت
Maryam	ghazayee	baraaye	khahare	dooste	man	pokht
Maryam	a dish	for	sister (of)	friend (of)	me	cooked
Proper name	indef. Noun	prep	noun	noun	obj. pronoun	heavy verb
و	خیلی	خوشمزه	بود			
va	kheili	khoshmazzeh	bood			
and	very	delicious	was			
coordinator	quantifier	adj	verb			

2

a. علی آهنگی برای من زد که مرا به سالهای نوجوانی برد

b. علی آهنگی برای جشنِ تولدِ من زد که مرا به سالهای نوجوانی برد

علی	آهنگی	برای	جشن	تولد	من	زد
Ali	ahangi	baraye	jashne	tavallode	man	zad
Ali	a (piece of) music	for	celebration (of)	birthday (of)	me	hit
Proper name	indef. Noun	prep	noun	noun	obj. pronoun	light verb
و	مرا	به	سالهای	نوجوانی	برد	
va	mara	be	sal-haye	nojavani	bord	
and	me-ra	to	years (of)	teenage	took	
coordinator	obj. pronoun + accus. Marker	prep	noun	noun	verb	

c. علی شکلاتی برای من خرید که مزه‌اش حرف نداشت

d. علی شکلاتی برای جشنِ تولدِ من خرید که مزه‌اش حرف نداشت

علی	شکلاتی	برای	جشن	تولد	من	خرید
Ali	shokolati	baraye	jashne	tavallode	man	kharid
Ali	a chokolate	for	celebration (of)	birthday (of)	me	bought
Proper name	indef. Noun	prep	noun	noun	obj. pronoun	heavy verb

و	مزه اش	حرف	نداشت			
va	mazze'ash	hard	nadasht			
and	its flavor	word	didn't have			
coordinator	noun + possessive adj.	pre-verbal noun	verb			

3

a. مادرم دعایی برای من کرد که همیشه موفق و سلامت باشم
b. مادرم دعایی برای دخترخاله‌ی دوستِ من کرد که همیشه موفق و سلامت باشد

مادرم	دعایی	برای	دخترخاله	دوست	من	کرد
Madaram	do'ayee	baraye	dokhtar-khaleye	doost-e	man	kard
My mother	a prayer	for	cousin (of)	friend (of)	me	did
Noun + possessive adj	indef. Noun	prep	noun	noun	obj. pronoun	light verb
تا	همیشه	موفق	باشد			
ta	hamisheh	movaffah	bashad			
so that	always	successful	be			
subordinator	adverb	adj	verb			

c. مادرم لباسی برای من دوخت که زمستان آن را بپوشم
d. مادرم لباسی برای دخترخاله‌ی دوستِ من دوخت که زمستان آن را بپوشد

مادرم	لباسی	برای	دخترخاله	دوست	من
Madaram	lebasi	baraye	dokhtar-khaleye	doost-e	man
My mother	a dress	for	cousin (of)	friend (of)	me
Noun + possessive adj	indef. Noun	prep	noun	noun	obj. pronoun
دوخت	تا	زمستان	آن	را	بپوشد
dookht	ta	zemestan	an	ra	bepooshad
sewed	so that	(in) winter	that	ra	wear
heavy verb	subordinator	adverb	demonst. Pronoun	accus. Marker	verb

4

a. حمید تقاضایی از مینا کرد که کاملا غیر منتظره بود
b. حمید تقاضایی از خواهرزاده‌ی همسایه‌ی مینا کرد که کاملا غیر منتظره بود

حمید	تقاضایی	از	خواهرزاده	همسایه	مینا
Hamid	taghazayee	az	Khaharzade-e	hamsaye-ye	Mina
Hamid	a request	from	cousin (of)	neighbor (of)	Mina
Proper name	indef. Noun	prep	noun	noun	Proper name
کرد	و	منتظر	جواب	شد	
kard	va	montazere	javad	shod	
did	and	wait (for)	reply	did	
light verb	coordinator	noun	noun	verb	

c. حمید جوابی از مینا شنید که او را شوکه کرد
d. حمید جوابی از خواهرزاده‌ی همسایه‌ی مینا شنید که او را شوکه کرد

حمید	جوابی	از	خواهرزاده	همسایه	مینا
Hamid	javabi	az	khaharzade-ye	hamsaye-ye	Mina
Hamid	a reply	from	cousin (of)	neighbor (of)	Mina
Proper name	indef. Noun	prep	noun	noun	Proper name
شنید	و	شوکه	شد		
shenid	va	shokkeh	shod		
heard	and	shocked	got		
heavy verb	coordinator	adj	verb		

5

a. حسن شکایتی از همسایه‌اش کرد اما بعد متوجه شد اشتباه می‌کرده است
b. حسن شکایتی از همسایه‌ی محله‌ی قدیمش شنید اما بعد متوجه شد اشتباه می‌کرده است

حسن	شکایتی	از	همسایه	محل	قدیمش	کرد
Hassan	shekayati	az	hamseye-ye	mahall-e	ghadimash	kard
Hassan	a complaint	from	neighbor (of)	neighborhood (of)	old time	did
Proper name	indef. Noun	prep	noun	noun	adj	light verb
اما	بعد	از	او	عذرخواهی	کرد	
amma	ba'd	az	oo	ozr-khahi	kard	
but	then	from	him/her	apology	did	
coordinator	adverb	prep	obj. pronoun	noun	verb	

c. حسن داستانی از همسایه‌اش شنید و نمی‌دانست باید آن را باور کند یا نه
d. حسن داستانی از همسایه‌ی محله‌ی قدیمش شنید و نمی‌دانست باید آن را باور کند یا نه

حسن	داستانی	از	همسایه	محل	قدیمش	شنید
Hassan	dastani	az	hamsaye-ye	mahall-e	ghadimash	shenid
Hassan	a story	from	neighbor (of)	neighborhood (of)	old time	heard
Proper name	indef. Noun	prep	noun	noun	adj	heavy verb
و	نمی دانست	باید	آن	را	باور کند	یا نه
va	nemidanest	bayad	an	ra	bavar-konad	ya na
and	didn't know	must	that	ra	believe	or not
coordinator	verb(negative)	modal	demonst. Pronoun	accus. Marker	verb	prep-negative marker

6

a. خبرنگار برداشتی از حرفهای من کرد که درست نبود
b. خبرنگار برداشتی از حرفهای آن مردِ بی‌خانمان کرد که درست نبود

خبرنگار	برداشتی	از	حرفهای	مرد	بی خانمان
Khabar-negar	bardashti	az	harf-haye	mard-e	bi-khaneman

(the) journalist	an impression	from	words (of)	man	homeless
Noun	indef. Noun	prep	noun	noun	adj
کرد	در حالی که	درست	نبود		
kard	dar hali ke	dorost	nabood		
did	while	correct	was not		
light verb	coordinator	adj	verb (negative)		

c. خبرنگار گزارشی از حرفهای من نوشت و آن را در روزنامه چاپ کرد

d. خبرنگار گزارشی از حرفهای آن مردِ بی‌خانمان نوشت و آن را در روزنامه چاپ کرد

خبرنگار	گزارشی	از	حرفهای	مرد	بی خانمان
Khabarnegar	gozareshi	az	harf-haye	mard-e	bi-khaneman
(the) journalist	a report	from	words (of)	man	homeless
noun	indef. Noun	prep	noun	noun	adj
نوشت	در حالی که	درست	نبود		
nevesht	dar hali ke	dorost	nabood		
wrote	while	correct	was not		
heavy verb	coordinator	adj	verb (negative)		

7

a. سپیده تحلیلی از مقاله کرد که بسیار چالش برانگیز بود

b. سپیده تحلیلی از مقاله اخیر چامسکی کرد که بسیار چالش برانگیز بود

سپیده	تحلیلی	از	مقاله	اخیر	چامسکی	کرد
Sepideh	tahlili	az	maghale-ye	akhir-e	Chomskey	kard
Sepideh	an analysis	from	paper (of)	latest	Chomskey	did
Proper name	indef. Noun	prep	noun	adj	Proper name	light verb
و	آن	را	در	کنفرانس	ارائه داد	
va	an	ra	dar	conferans	era'eh dad	
and	that	ra	in	conference	presented	
coordinator	demonst. Pronoun	accus. Marker	prep	noun	verb	

c. سپیده نقدی از مقاله خواند که بسیار چالش برانگیز بود

d. سپیده نقدی از مقاله‌ی اخیرِ چامسکی خواند که بسیار چالش برانگیز بود

سپیده	نقدی	از	مقاله	اخیر	چامسکی	خواند
Sepideh	naghdi	az	maghale-ye	akhir-e	Chomskey	khand
Sepideh	a critisism	from	paper (of)	latest	Chomskey	read
Proper name	indef. Noun	prep	noun	adj	Proper name	heavy verb
و	در	سخنرانی	خود	به	آن	اشاره کرد
va	dar	sokhanrani-e	khod	be	an	eshareh-kard
and	in	conference	herself	to	that	pointed

		(of)				
coordinator	prep	noun	reflexive pronoun	prep	demonst. Pronoun	verb

8

a. سارا انتظاری از من داشت که نمی‌توانستم برآورده کنم
b. سارا انتظاری از پدرِ ورشکسته‌ی ما داشت که نمی‌توانست برآورده کند

سارا	انتظاری	از	پدر	ورشکسته	ما	داشت
Sara	entezari	az	pedar-e	varshekaste-ye	ma	dasht
Sara	an expectation	from	father	bankrupt	(of)us	had
Proper name	indef. Noun	prep	noun	adj	obj. pronoun	light verb
که	نمی توانست	آن	را	برآورده کند		
ke	nemitavanest	an	ra	baravardeh-konad		
that	could not	that	ra	meet		
relative marker	verb(negative)	demonst. Pronoun	accus. Marker	verb		

c. سارا خاطره‌ای از من داشت که من پاک یادم رفته بود
d. سارا خاطره‌ای از پدر ورشکسته‌ی ما داشت که من پاک یادم رفته بود

سارا	خاطره ای	از	پدر	ورشکسته	ما	داشت
Sara	khatere-i	az	pedar-e	varshekaste-ye	ma	dasht
Sara	a memory	from	father	bankrupt	(of) us	had
Proper name	indef. Noun	prep	noun	adj	obj. pronoun	heavy verb
که	من	پاک	یادم رفته بود			
ke	man	pak	yadam rafteh bood			
that	I	totally	had forgotten			
relative marker	subj. pronoun	adverb	verb			

9

a. همکارم سهمی از شرکت برد که اصلا منصفانه نبود
b. همکارم سهمی از شرکتِ تجاری بازرگانی برد که اصلا منصفانه نبود

همکارم	سهمی	از	شرکت	تجاری	بازرگانی	برد
Hamkaram	sahmi	az	sherkat-e	tejari	bazargani	bord
My colleague	a share	from	company	trading	comercial	won
Noun + possessive adj	indef. Noun	prep	noun	adj	adj	light verb
که	اصلا	منصفانه	نبود			
ke	aslan	monsefaneh	nabood			
that	at all	fair	was not			
relative marker	quantifier	adj	verb (negative)			

c. همکارم انتقادی از شرکت داشت که در جلسه‌ی هیئت مدیره مطرح کرد
d. همکارم انتقادی از شرکتِ تجاری بازرگانی داشت که در جلسه‌ی هیئت مدیره مطرح کرد

همکارم	انتقادی	از	شرکت	تجاری	بازرگانی	داشت

Hamkaram	enteghadi	az	sherkat-e	tejari	bazargani	dasht
My colleague	a critisism	from	company	trading	commercial	had
Noun+possessive adj	indef. Noun	prep	noun	adj	adj	heavy verb
که	در	جلسه	هیئت مدیره	مطرح کرد		
ke	dar	jalaseh-ye	heiat modireh	matrah kard		
that	in	meeting (of)	board of directors	mentioned		
relative marker	prep	noun	noun	verb		

10

a. من خواهشی از مهسا کردم اما او آن را رد کرد

b. من خواهشی از همکلاسیِ برادرِ مهسا کردم اما او آن را رد کرد

من	خواهشی	از	همکلاسی	برادر	مهسا	کردم
Man	khaheshi	az	hamkelasi-e	baradar-e	Mahsa	kardam
I	a request/wish	from	classmate (of)	brother (of)	Mahsa	did
Subj. pronoun	indef. Noun	prep	noun	noun	proper name	light verb
اما	او	آن	را	رد کرد		
amma	oo	an	ra	rad kard		
but	she/he	that	ra	rejected		
coordinator	subj. pronoun	demonst. Pronoun	accus. Marker	verb		

c. من پولی از مهسا گرفتم چون کیفم را در خانه جا گذاشته بودم

d. من پولی از همکلاسیِ برادرِ مهسا گرفتم چون کیفم را در خانه جا گذاشته بودم

من	پولی	از	همکلاسی	برادر	مهسا	گرفتم
Man	pooli	az	hamkelasi-e	baradar-e	Mahsa	gereftam
I	a money	from	classmate (of)	brother (of)	Mahsa	got
Subj. pronoun	indef. Noun	prep	noun	noun	proper name	heavy verb
چون	کیفم	را	در	خانه	جا گذاشته بودم	
chon	kifam	ra	dar	khaneh	ja gozashteh boodam	
because	my bag	ra	at	home	had left	
coordinator	noun+possessive adj	accus. Marker	prep	noun	verb	

11

a. الهام عطری به لباسش زد که توجهِ همه را جلب کرده بود

b. الهام عطری به لباسِ قرمزِ جدیدش زد که توجهِ همه را جلب کرده بود

الهام	عطری	به	لباس	قرمز	جدیدش	زد
Elham	atri	be	lebas-e	ghermez-e	jadidash	zad
Elham	a perfume	to	dress	red	new	hit
Proper name	indef. Noun	prep	noun	adj	adj + possessive adj	light verb

که	توجه	همه	را	جلب کرده بود		
ke	tavajjoh-e	hameh	ra	jalb-kardeh-bood		
that	attention (of)	everybody	ra	had drawn		
relative marker	noun	noun	accus. Marker	verb		

.c الهام سنجاقی به لباسِ آویخت که پروانه‌ای نقره‌ای رنگ بود
.d الهام سنجاقی به لباسِ قرمزِ جدیدش آویخت که پروانه‌ای نقره‌ای رنگ بود

الهام	سنجاقی	به	لباس	قرمز	جدیدش	آویخت
Elham	sanjaghi	be	lebas-e	ghermez-e	jadidash	avikht
Elham	a clips	to	dress	red	new	hanged
Proper name	indef. Noun	prep	noun	adj	adj+ possessive adj	heavy verb
که	پروانه ای	نقره ای رنگ	بود			
ke	parvane-i	noghrei-rang	bood			
that	a butherfly	silver	was			
relative marker	noun	adj	verb			

12

.a محسن اتهامی به من زد اما نتوانست آن را اثبات_کند
.b محسن اتهامی به خواهرزاده‌ی هم‌اتاقیِ من زد اما نتوانست آن را اثبات کند

محسن	اتهامی	به	خواهرزاده	هم اتاقی	من	زد
Mohsen	ettehami	be	khaharzade-ye	ham-otaghi-e	man	zad
Mohsen	an accusation	to	niece/nephew	roommate (of)	me	hit
Proper name	indef. Noun	prep	noun	noun	obj. pronoun	light verb
اما	نتوانست	آن	را	ثابت کند		
amma	natavanest	an	ra	sabet-konad		
but	could not	that	ra	prove		
coordinator	modal(negative)	demonst. Pronoun	accus. Marker	verb		

.c محسن رازی به من گفت که حسابی مرا در فکر فرو برد
.d محسن رازی به خواهرزاده‌ی هم‌اتاقیِ من گفت که حسابی او را در فکر فرو برد

محسن	رازی	به	خواهرزاده	هم اتاقی	من	گفت
Mohsen	razi	be	khahar-zade-ye	ham-otaghi-e	man	goft
Mohsen	a secret	to	niece/ nephew (of)	roommate (of)	me	said
Proper name	indef. Noun	prep	noun	noun	obj. pronoun	verb
و	حسابی	او	را	در	فکر	فرو برد
va	hesabi	oo	ra	dar	fekr	foroo-bord

and	very much	him/her	ra	in	thought	drowned
coordinator	quantifier	obj. pronoun	accus. Marker	pre-verbal prep	preverbal noun	verb

13

a. رضا لبخندی به من زد که هرگز از یاد نمی‌برم

b. رضا لبخندی به پدرِ بیمار من زد که هرگز از یاد نمی‌برم

رضا	لبخندی	به	پدر	بیمار	من	زد
Reza	labkhandi	be	pedar-e	bimar-e	man	zad
Reza	a smile	to	father	ill	me	hit
Proper name	indef. Noun	prep	noun	adj	obj. pronoun	light verb
که	هرگز	آن	را	فراموش نمی کنم		
ke	hargez	an	ra	faramoosh nemikonam		
that	never	that	ra	don't forget		
relative marker	adverb	demonst. Pronoun	accus. Marker	verb(negative)		

c. رضا باغی به من فروخت که پر از درخت‌های زردآلو و گیلاس بود

d. رضا باغی به پدرِ بیمار من فروخت که پر از درخت‌های زردآلو و گیلاس بود

رضا	باغی	به	پدر	بیمار	من	فروخت
Reza	baghi	be	pedar-e	bimar-e	man	forookht
Reza	a garden	to	father	ill	me	sold
Proper name	indef. Noun	prep	noun	adj	obj. pronoun	heavy verb
و	از	این	شهر	رفت		
va	az	in	shahr	raft		
and	from	this	city	went		
coordinator	prep	demonst. Adj	noun	verb		

14

a. مژگان تهمتی به من زد که باعث شد دیگر با او قطع رابطه کنم

b. مژگان تهمتی به نزدیک‌ترین دوستِ من زد که باعث شد دیگر با او قطع رابطه کنم

مژگان	تهمتی	به	نزدیکترین	دوست	من	زد
Mojgan	tohmati	be	nazdiktarin	doost-e	man	zad
Mojgan	an insult	to	(the) closest	friend (of)	me	hit
Proper name	indef. Noun	prep	adj	noun	obj. pronoun	light verb
و	باعث شد	با	او	قطع رابطه	کنم	
va	ba'es shod	ba	oo	ghat'e rabeteh	konam	
and	caused	with	him/her	cut-relation	do	
coordinator	verb	prep	obj. pronoun	noun	verb	

c. مژگان هدیه‌ای به من داد که واقعا ذوق کردم

d. مژگان هدیه‌ای به پسرِ بامزه‌ی من داد که واقعا ذوق کرد

مژگان	هدیه ای	به	پسر	بامزه	من	داد
Mojgan	hedye-i	be	pesar-e	bamazze-ye	man	heavy dad
Mojgan	a gift	to	son	cute	me	gave
Proper name	indef. Noun	prep	noun	adj	obj. pronoun	Heavy verb
که	واقعا	ذوق کرد				
ke	vaghe'an	zogh kard				
that	really	got excited				
relative marker	adverb	verb				

15

a. همسرم ضربه‌ای به زندگی من زد که غیرِقابل جبران است

b. همسرم ضربه‌ای به زندگیِ کسالت‌بارِ من زد که غیرقابل جبران است

همسرم	ضربه ای	به	زندگی	کسالت بار	من	زد
Hamsaram	zarbe-i	be	zendegi-e	kesalat-bar-e	man	zad
My spouse	a damage	to	life	boring	me	hit
Noun+possessive adj	indef. Noun	prep	noun	adj	obj. pronoun	light verb
که	غیرقابل جبران	بود				
ke	gheir-e ghabel-e jobran	bood				
that	uncompensable	was				
relative marker	adj	verb				

c. همسرم شادی به زندگیِ من آورد که قابلِ وصف نبود

d. همسرم شادی به زندگیِ کسالت‌بارِ من آورد که قابل وصف نبود

همسرم	شادی	به	زندگی	کسالت بار	من	آورد
Hamsaram	shadi	be	zendegi-e	kesalat-bar-e	man	avard
My spouse	a hapinness	to	life	boring	me	brought
Noun+possessive adj	indef. Noun	prep	noun	adj	obj. pronoun	heavy verb
که	قابل وصف	نبود				
ke	ghabel-e vasf	nabood				
that	describable	was not				
relative marker	adj	verb(negative)				

16

a. فرزاد حرفی به من زد که خیلی جا خوردم

b. فرزاد حرفی به استادِ زبانِ من زد که خیلی جا خوردم

فرزاد	حرفی	به	استاد	زبان	من	زد
Farzad	harfi	be	ostad-e	zaban-e	man	zad
Farzad	a word	to	professor (of)	language	me	hit

Proper name	indef. Noun	prep	noun	noun	obj. pronoun	light verb
که	خیلی	جا خوردم				
ke	kheili	ja khordam				
that	so much	got shocked				
relative marker	quantifier	verb				

c. فرزاد کتابی به من داد که در موردِ ستارگان بود
d. فرزاد کتابی به استادِ زبانِ من داد که در موردِ ستارگان بود

فرزاد	کتابی	به	استاد	زبان	من	داد
Farzad	ketabi	be	ostad-e	zaban-e	man	dad
Farzad	a book	to	professor (of)	language	me	gave
Proper name	indef. Noun	prep	noun	noun	obj. pronoun	heavy verb
که	در مورد	ستارگان	بود			
ke	dar morede	setaregan	bood			
that	about	(the) stars	was			
relative marker	prep	noun	verb			

17

a. معلم توصیه‌ای به من کرد که بسیار مفید بود
b. معلم توصیه‌ای به شاگردانِ سالِ آخر کرد که بسیار مفید بود

معلم	توصیه ای	به	شاگردان	سال	آخر	کرد
Moallemam	tosie-i	be	shagerdan-e	sal-e	akhar	kard
My teacher	a recommendation	to	students (of)	year	last	did
Noun+possessive adj	indef. Noun	prep	noun	noun	adj	light verb
که	بسیار	مفید	بود			
ke	besyar	mofid	bood			
that	very	useful	was			
relative marker	quantifier	adj	verb			

c. معلم درسی به من آموخت که هرگز آن را فراموش نمی‌کنم
d. معلم درسی به شاگردانِ سالِ آخر آموخت که هرگز آن را فراموش نمی‌کنند

معلم	درسی	به	شاگردان	سال	آخر	آموخت
Moallemam	darsi	be	shagerdan-e	sal-e	akhar	amookht
My teacher	a lesson	to	students (of)	year	last	taught
Noun+possessive adj	indef. Noun	prep	noun	noun	adj	heavy verb
که	هرگز	آن	را	فراموش نمی کنند		
ke	hargez	an	ra	faramoosh nemikonam		
that	never	that	ra	forget		

relative marker	adverb	demonst. Pronoun	accus. Marker	verb(negative)		

18

a. کوروش نصیحتی به من کرد که خیلی به جا بود
b. کوروش نصیحتی به برادرِ افسرده‌ی من کرد که خیلی به جا بود

کوروش	نصیحتی	به	برادر	افسرده	من	کرد
Kourosh	nasihati	be	baradar-e	afsorde-ye	man	kard
Kourosh	an advice	to	brother	depressed	me	did
Proper name	indef. Noun	prep	noun	adj	obj. pronoun	light verb
که	خیلی	به جا	بود			
ke	kheili	be-ja	bood			
that	very	timely	was			
relative marker	quantifier	adj	verb			

c. کوروش نکته‌ای به من فهماند که خیلی به جا بود
d. کوروش نکته‌ای به برادرِ افسرده‌ی من فهماند که خیلی به جا بود

کوروش	نکته ای	به	برادر	افسرده	من	فهماند
Kourosh	noktei	be	baradar-e	afsorde-ye	man	fahmand
Kourosh	a point	to	brother	depressed	me	made across
Proper name	indef. Noun	prep	noun	adj	obj. pronoun	heavy verb
که	خیلی	به جا	بود			
ke	kheili	be-ja	bood			
that	very	timely	was			
relative marker	quantifier	adj	verb			

19

a. شبنم پیشنهادی به من کرد که غیرِ منتظره بود
b. شبنم پیشنهادی به پسرخاله‌ی دوستِ من کرد که غیرِ منتظره بود

شبنم	پیشنهادی	به	پسرخاله	دوست	من	کرد
Shabnam	pishnahadi	be	pesarkhale-ye	doost-e	man	kard
Shabnam	a suggestion	to	cousin (of)	friend (of)	me	did
Proper name	indef. Noun	prep	noun	noun	obj. pronoun	light verb
که	غیرمنتظره	بود				
ke	gheir-e montazereh	bood				
that	unexpected	was				
relative marker	adj	verb				

c. شبنم تابلویی به من بخشید که خیلی گرانقیمت بود
d. شبنم تابلویی به پسرخاله‌ی دوستِ من بخشید که خیلی گرانقیمت بود

شبنم	تابلویی	به	پسرخاله	دوست	من	بخشید
Shabnam	tablo'yee	be	pesarkhale-ye	doost-e	man	bakhshid
Shabnam	a picture	to	cousin (of)	friend (of)	me	spared
Proper name	indef. Noun	prep	noun	noun	obj. pronoun	heavy verb
که	خیلی	گران قیمت	بود			
ke	kheili	geran-gheymat	bood			
that	very	expensive	was			
relative marker	quantifier	adj	verb			

20

a. بهنام راهنمایی به من کرد که قابل قبول بود

b. بهنام راهنمایی به دانشجوی ممتازِ خود کرد که قابلِ قبول بود

بهنام	راهنمایی	به	دانشجوی	ممتاز	خود	کرد
Behnam	rahnamayee	be	daneshjooye	momtaz-e	khod	kard
Behnam	a guidance	to	student	merit	(of) his	did
Proper name	indef. Noun	prep	noun	adj	reflexive pronoun	light verb
که	قابل قبول	بود				
ke	ghabele ghabool	bood				
that	acceptable	was				
relative marker	adj	verb				

c. بهنام مقاله‌ای به من داد که درست در موردِ موضوعِ تحقیقم بود

d. بهنام مقاله‌ای به دانشجوی ممتازِ خود داد که درست در موردِ موضوعِ تحقیقش بود

بهنام	مقاله ای	به	دانشجوی	ممتاز	خود	داد
Behnam	maghale-i	be	daneshjooye	momtaz-e	khod	dad
Behnam	an article	to	student	merit	(of) his	gave
Proper name	indef. Noun	prep	noun	adj	reflexive pronoun	heavy verb
که	درست	در مورد	موضوع	تحقیقش	بود	
ke	dorost	dar morede	mozoo'e	tahghigh-ash	bood	
that	right	about	topic (of)	his/her research	was	
relative marker	adj	prep	noun	noun+possessive adj	verb	

21

a. همسایه‌ام تذکری به دوستم داد تا کمتر سیگار بکشد

b. همسایه‌ام تذکری به پدر سالخورده‌ی دوستم داد تا کمتر سیگار بکشد

همسایه ام	تذکری	به	پدر	سالخورده	دوستم	داد
Hamsaye'am	tazakkori	be	pedar-e	salkhorde-ye	doostam	dad
My neighbor	a point-out	to	father	old	my friend	gave

Noun+possessive adj	indef. Noun	prep	noun	adj	noun+possessive adj	light verb
که	دیگر	سیگار	نکشد			
ke	digar	sigar	nakeshad			
so that	from that time	smoke	doesn't			
relative marker	adverb	noun	verb(negative)			

c. همسایه‌ام دوایی به دوستم رساند که بسیار نجات بخش بود

d. همسایه ام دوایی به پدرِ سالخورده‌ی دوستم رساند که بسیار نجات بخش بود

همسایه ام	دوایی	به	پدر	سالخورده	دوستم	رساند
Hamsaye'am	davayee	be	pedar-e	salkhorde-ye	doostam	resand
My neighbor	a medication	to	father	old	my friend	fetched
Noun+possessive adj	indef. Noun	prep	noun	adj	noun+possessive adj	heavy verb
که	بسیار	نجات بخش	بود			
ke	besyar	nejat-bakhsh	bood			
that	very	life-saving	was			
relative marker	quantifier	adj	verb			

22

a. نیما سفارشی به من کرد تا مراقبِ خواهرش باشم

b. نیما سفارشی به شوهر خواهرِ بزرگش کرد تا مراقبِ خواهرش باشد

نیما	سفارشی	به	شوهر	خواهر	بزرگش	کرد
Nima	sefareshi	be	shohar-e	khahar-e	bozorgash	kard
Nima	an request	to	husband (of)	sister	older	did
Proper name	indef. Noun	prep	noun	noun	adj+ possessive adj	light verb
تا	مراقب	خواهرش	باشد			
ta	moragheb-e	khaharash	bashad			
so that	looking after	his sister	be			
coordinator	noun	noun+possessive adj	verb			

c. نیما امانتی به من سپرد که بسیار برایش با ارزش بود

d. نیما امانتی به شوهر خواهرِ بزرگش سپرد که بسیار برایش با ارزش بود

نیما	امانتی	به	شوهر	خواهر	بزرگش	سپرد
Nima	amanati	be	shohar-e	khahar-e	bozorgash	sepord
Nima	a borrowing	to	husband (of)	sister	older	gave
Proper name	indef. Noun	prep	noun	noun	adj+ possessive adj	heavy verb
که	بسیار	برایش	با ارزش	بود		
ke	besyar	barayash	ba-arzesh	bood		
that	very	for him	valuable	was		
relative	quantifier	prep+possessive	adj	verb		

marker		adj				

23

a. آدم‌رباها هشداری به من دادند که اگر پول را آماده نکنم پسرم را می‌کشند

b. آدم‌رباها هشداری به خانواده‌ی کودکِ ربوده‌شده دادند که اگر پول را آماده نکنند پسرشان را می‌کشند

آدم رباها	هشداری	به	خانواده	کودک	ربوده شده	دادند
Adam-roba-ha	hoshdari	be	khanevade-ye	koodak-e	roboodeh-shode	dadand
(the) kidnappers	a warning	to	the family (of)	baby	kidnapped	gave
Noun	indef. Noun	prep	noun	noun	adj	light verb
اگر	پول	را	آماده نکنند	پسرشان	را	می کشند
agar	pool	ra	amadeh nakonand	pesareshan	ra	mikoshand
if	money	ra	don't prepare	their son	ra	kill
subordinator	noun	accus. Marker	verb(negative)	noun+possessive adj	accus. Marker	verb

c. آدم‌رباها اخطاری به من فرستادند که اگر پول را آماده نکنم پسرم را می‌کشند

d. آدم‌رباها اخطاری به خانواده‌ی کودکِ ربوده‌شده فرستادند که اگر پول را آماده نکنند پسرشان را می‌کشند

آدم رباها	اخطاری	به	خانواده	کودک	ربوده شده	فرستادند	
Adam-roba-h	ekhtari	be	khanevadey e	koodak-e	roboodeh-shodeh	ferestadan d	
(the) kidnappers	a warning	to	family (of)	baby	kidnapped	sent	
noun	indef. Noun	prep	noun	noun	adj	heavy verb	
که	اگر	پول	را	آماده نکنند	پسرشان	را	می کشند
ke	agar	pool	ra	amadeh nakonand	pesareshan	ra	mikoshan d
that	if	money	ra	don't prepare	their son	ra	kill
relative marker	subordinato r	noun	accus. Marker	verb(negative)	noun+possessiv e adj	accus. Marker	verb

24

a. فرهاد قولی به من داد که خیالم را راحت کرد

b. فرهاد قولی به خواهرِ مهربانِ من داد که خیالش را راحت کرد

فرهاد	قولی	به	خواهر	مهربان	من	داد	که
Farhad	gholi	be	khahar-e	mehraban-e	man	dad	ke
Farhad	a promise	to	sister	kind	me	gave	that
Proper name	indef. Noun	prep	noun	adj	obj. pronoun	light verb	relative marker
خیالش	را	راحت کرد					
khialash	ra	rahat-kard					
her mind	ra	released					
Noun+possessive adj	accus. Marker	verb					

c. فرهاد انگشتری به من داد که از طلا بود

d. فرهاد انگشتری به خواهرِ مهربانِ من داد که از طلا بود

فرهاد	انگشتری	به	خواهر	مهربان	من	داد
Farhad	angoshtari	be	khahar-e	mehraban-e	man	dad
Farhad	a ring	to	sister	kind	me	gave
Proper name	indef. Noun	prep	noun	adj	obj. pronoun	heavy verb
از	طلا	بود				
az	tala	bood				
from	gold	was				
prep	noun	verb				

25

a. میترا دروغی به من گفت که هرگز او را نمی‌بخشم

b. میترا دروغی به دخترِ کوچکِ من گفت که هرگز او را نمی‌بخشم

میترا	دروغی	به	دختر	کوچک	من	گفت
Mitra	dorooghi	be	dokhtar-e	koochak-e	man	goft
Mitra	a lie	to	daughter	little	me	told
Proper name	indef. Noun	prep	noun	adj	obj. pronoun	light verb
هرگز	او	را	نمی بخشم			
hargez	oo	ra	nemibakhsham			
never	her	ra	don't forget (i)			
adverb	obj. pronoun	accus. Marker	verb(negative)			

c. میترا عروسکی به من داد که درست قدِ خودِ من بود

d. میترا عروسکی به دخترِ کوچکِ من داد که درست قدِ خودِ او بود

میترا	عروسکی	به	دختر	کوچک	من	داد	که
Mitra	aroosaki	be	dokhtar-e	koochak-e	man	dad	ke
Mitra	a doll	to	sister	little	me	gave	that
Proper name	indef. Noun	prep	noun	adj	obj. pronoun	heavy verb	relative marker
درست	قدِ	خودش	بود				
dorost	ghadd-e	khodash	bood				
exactly	the hight (of)	her self	was				
adverb	noun	reflexive pronoun	verb				

26

a. نیلوفر پیشرفتی در مدرسه کرد که همه را بهتزده کرده بود

b. نیلوفر پیشرفتی در مدرسه‌ی دولتیِ معرفت کرد که همه را بهتزده کرده بود

نیلوفر	پیشرفتی	در	مدرسه	دولتی	معرفت	کرد	که
Niloofar	pishrafti	dar	madrese-ye	dolati-e	Ma'refat	kard	ke
Niloofar	a progress	at	school	governmental	Ma'refat	did	that
Proper name	indef. Noun	prep	noun	adj	proper name	light verb	relative marker

همه	را	بهت زده	کرده بود				
hameh	ra	boht-zadeh	karde-bood				
everybody	ra	amazed	had done				
noun	accus. Marker	adj	verb				

c. نیلوفر انشایی در مدرسه خواند که همه را تحتِ تاثیر قرار داد

d. نیلوفر انشایی در مدرسه‌ی دولتیِ معرفت خواند که همه را تحتِ تاثیر قرار داد

نیلوفر	انشایی	در	مدرسه	دولتی	معرفت	خواند	که
Niloofar	enshayee	dar	madrese-ye	dolati-e	Ma'refat	khand	ke
Niloofar	a composition	at	school	governmental	Ma'refat	read out	that
Proper name	indef. Noun	prep	noun	adj	proper name	heavy verb	relative marker
همه	را	تحت تاثیر	قرار داده بود				
hameh	ra	that-e ta'sir	gharar dadeh-bood				
everybody	ra	impressed	had done				
noun	accus. Marker	adj	verb				

27

a. مجید لطفی به من کرد که تا عمر دارم فراموش نمی‌کنم

b. مجید لطفی به برادرِ جوانِ من کرد که تا عمر دارم فراموش نمی‌کنم

مجید	لطفی	به	برادر	جوان	من	کرد	که
Majid	lotfi	be	baradar-e	javan-e	man	kard	ke
Majid	a favor	to	brother	young	me	did	that
Proper name	indef. Noun	prep	noun	adj	obj. pronoun	light verb	relative marker
تا	عمر	دارم	فراموش نمی کنم				
ta	omr	daram	faramoosh nemikonam				
till	life	have (i)	don't forget				
prep	noun	verb	verb(negative)				

c. مجید ناسزایی به من گفت که تا عمر دارم او را نمی‌بخشم

d. مجید ناسزایی به برادرِ جوانِ من گفت که تا عمر دارم او را نمی‌بخشم

مجید	ناسزایی	به	برادر	جوان	من	گفت	که
Majid	na-sezayee	be	baradar-e	javan-e	man	goft	ke
Majid	a swearword	to	brother	young	me	said	that
Proper name	indef. Noun	prep	noun	adj	obj. pronoun	heavy verb	relative marker
تا	عمر	دارم	او	را	نمی بخشم		
ta	omr	daram	oo	ra	nemibakhsham		
till	life	have (i)	him/her	ra	don't forgive		
prep	noun	verb	obj. pronoun	accus. Marker	verb(negative)		

28

a. استاد صحبتی با شاگردش کرد که بسیار در او اثر گذاشت
b. استاد صحبتی با شاگردِ قدیمیِ خود کرد که بسیار در او اثر گذاشت

استاد	صحبتی	با	شاگرد	قدیمی	خود	کرد	که
Ostad	sohbati	ba	shagerd-e	ghadimi-e	khod	kard	ke
Professor	a word	with	student	old	(of) him/her self	did	that
Noun	indef. Noun	prep	noun	adj	reflexive pronoun	light verb	relative marker
بسیار	در	او	اثر گذاشت				
besyar	dar	oo	asar-gozasht				
so much	in	him/her	influenced				
quantifier	prep	obj. pronoun	verb				

c. استاد قطعه‌ای با شاگردش نواخت که بسیار برایم خاطره انگیز بود
d. استاد قطعه‌ای با شاگردِ قدیمیِ خود نواخت که بسیار برایم خاطره انگیز بود

استاد	قطعه ای	با	شاگرد	قدیمی	خود	نواخت
Ostad	ghat'ei	ba	shagerd-e	ghadimi'e	khod	navakht
Professor	a piece (of music)	with	student	old	(of) him/her self	played
noun	indef. Noun	prep	noun	adj	reflexive pronoun	heavy verb
که	برایم	بسیار	خاطره انگیز	بود		
ke	barayam	besyar	khatereh-angiz	bood		
that	for me	very	memorable	was		
relative marker	prep+obj. pronoun	quantifier	adj	verb		

29

a. سینا قراری با ندا گذاشت اما ندا سرِ قرار نیامد
b. سینا قراری با برادرِ دوستِ ندا گذاشتم اما او سرِ قرار نیامد

سینا	قراری	با	برادر	دوست	ندا	گذاشت
Sina	gharari	ba	baradar-e	doost-e	Neda	gozasht
Sina	an appointment	with	brother (of)	friend (of)	Neda	put
Proper name	indef. Noun	prep	noun	noun	proper name	light verb
اما	او	سر	قرار	نیامد		
amma	oo	sare	gharar	nayamad		
but	he	to	(the) appointment	didn't come		
coordinator	subj. pronoun	prep	noun	verb(negative)		

c. سینا سفری با ندا رفت که خیلی به آنها خوش گذشت
d. سینا سفری با برادرِ دوستِ ندا رفت که خیلی به آنها خوش گذشت

سینا	سفری	با	برادر	دوست	ندا	رفت
Sina	safari	ba	baradar-e	doost-e	Neda	raft
Sina	a travel	with	brother	friend (of)	Neda	went

			(of)			
Proper name	indef. Noun	prep	noun	noun	proper name	heavy verb
که	به	آنها	خیلی	خوش گذشت		
ke	be	anha	kheili	khosh gozasht		
that	to	them	so much	fun had		
relative marker	prep+obj. pronoun	obj. pronoun	quantifier	verb		

30

a. من حدسی در موردِ ستاره زدم که اشتباه از آب درآمد

b. من حدسی در موردِ گذشته‌ی مبهمِ ستاره زدم که اشتباه از آب درآمد

من	حدسی	در مورد	گذشته	مبهم	ستاره	زدم
Man	hadsi	dar morede	gozashte-ye	mobham-e	Setareh	zadam
I	a guess	about	(the) past	ambiguous	Setareh	hit
Subj. pronoun	indef. Noun	prep	noun	adj	proper name	light verb
که	اشتباه	از	آب	درآمد		
ke	eshtebah	az	ab	dar-amad		
that	wrong	from	water	came out		
relative marker	adj	pre-verbal prep	pre-verbal noun	verb		

c. من شایعه‌ای در موردِ ستاره شنیدم که مایه‌ی خجالت بود

d. من شایعه‌ای در موردِ گذشته‌ی مبهمِ ستاره شنیدم که مایه‌ی خجالت بود

من	شایعه ای	در مورد	گذشته	مبهم	ستاره	شنیدم
Man	shaye'ei	dar morede	gozashte'ye	mobham'e	Setareh	shenidam
I	a rumor	about	(the) past	ambiguous	Setareh	heard
subj. pronoun	indef. Noun	prep	noun	adj	proper name	heavy verb
مایه	خجالت	بود				
maye'ye	khejalat	bood				
cause (of)	shame	was				
noun	noun	verb				

31

a. پژمان قضاوتی در موردِ من کرد که حقِ من نبود

b. پژمان قضاوتی در موردِ خواهرِ کوچکترِ من کرد که حقِ او نبود

پژمان	قضاوتی	در مورد	خواهر	کوچکتر	من	کرد
Pejman	ghezavati	dar morede	khahar-e	koochektar-e	man	kard
Pejman	a judgement	about	sister	younger	me	did
Proper name	indef. Noun	prep	noun	adj	obj. pronoun	light verb
حق	او	نبود				
hagh-e	oo	nabood				
right (of)	her	was not				
noun	obj.	verb(negative)				

	pronoun					

c. پژمان خبری در موردِ من شنید که نگرانش کرده بود

d. پژمان خبری در موردِ خواهرِ کوچکترِ من شنید که نگرانش کرده بود

پژمان	خبری	در مورد	خواهر	کوچکتر	من	شنید	که
Pejman	khabari	dar morede	khahar-e	koochektar'e	man	shenid	ke
Pejman	a (piece of) news	about	sister	younger	me	heard	that
Proper name	indef. Noun	prep	noun	adj	obj. pronoun	heavy verb	relative marker
نگرانش	کرده بود						
negaranash	kardeh-bood						
worried him	had done						
adj+ obj. pronoun	verb						

32

a. کاوه حسی در موردِ پریسا می‌کرد که برای خودش عجیب بود

b. کاوه حسی در موردِ خواهرِ بزرگِ پریسا می‌کرد که برای خودش عجیب بود

کاوه	حسی	در مورد	خواهر	بزرگتر	پریسا	می کرد
Kaveh	hessi	dar morede	khahar-e	bozorgtar-e	Parisa	mikard
Kaveh	a feeling	about	sister	older	Parisa	did
Proper name	indef. Noun	prep	noun	adj	proper name	light verb
که	برای	خودش	عجیب	بود		
ke	baraye	khodash	ajib	bood		
that	for	himself	weird	was		
relative marker	prep	reflexive pronoun	adj	verb		

c. کاوه واقعیتی در موردِ پریسا فهمید که تا مدت‌ها شوکه بود

d. کاوه واقعیتی در موردِ خواهرِ بزرگِ پریسا فهمید که تا مدت‌ها شوکه بود

کاوه	واقعیتی	در مورد	خواهر	بزرگتر	پریسا	فهمید
Kaveh	vagheiati	dar morede	khahar-e	bozorgtar-e	Parisa	fahmid
Kaveh	a truth	about	sister	older	Parisa	understood
Proper name	indef. Noun	prep	noun	adj	proper name	heavy verb
که	تا	مدت ها	شوکه	بود		
ke	ta	moddat-ha	shokkeh	bood		
that	till	long time	shocked	was		
relative marker	prep	adverb	adj	verb		

33

a. حامد استدلالی در موردِ مسئله کرد که به نظر منطقی می‌رسید

b. حامد استدلالی در موردِ مسئله‌ی مالیِ شرکت کرد که به نظر منطقی می‌رسید

حامد	استدلالی	در مورد	مسئله	مالی	شرکت	کرد

Hamed	estedlali	dar morede	mas'ale-ye	mali-e	sherkat	kard
Hamed	a reasoning	about	matter	financial	(of)company	did
Proper name	indef. Noun	prep	noun	adj	noun	light verb
که	به نظر	منطقی	می رسید			
ke	be nazar	manteghi	mi-resid			
that	to eye	logical	seemed			
relative marker	pre-verbal pp	adj	verb			

c. حامد چاره‌ای در موردِ مسئله اندیشید که بسیار کارآمد بود

d. حامد چاره‌ای در موردِ مسئله‌ی مالیِ شرکت اندیشید که بسیار کارآمد بود

حامد	چاره ای	در مورد	مسئله	مالی	شرکت	اندیشید
Hamed	chare'i	dar morede	mas'ale-ye	mali-e	sherkat	andishid
Hamed	a solution	about	matter	financial	(of) company	thought
Proper name	indef. Noun	prep	noun	adj	noun	heavy verb
که	بسیار	کارآمد	بود			
ke	besyar	kar-amad	bood			
that	very	efficient	was			
relative marker	quantifier	adj	verb			

34

a. حسین اشتباهی در موردِ همسرش کرد که باعثِ جدايي آنها شد

b. حسین اشتباهی در موردِ همسرِ مهربان و فداکارش کرد که باعثِ جدايي آنها شد

حسین	اشتباهی	در مورد	همسر	مهربان	و	فداکارش	کرد
Hossein	eshtebahi	dar morede	hamsar-e	mehraban	va	fadakarash	kard
Hossein	a mistake	about	wife	kind	and	devoted	did
Proper name	indef. Noun	prep	noun	adj	connector	adj	light verb
که	باعث	جدایی	آنها	شد			
ke	ba'ese	joda'ye	an-ha	shod			
which	cause (of)	separation	them	became			
relative marker	noun	noun	obj. pronoun	verb			

c. حسین چیزی در موردِ همسرش فهمید که خواب را از چشمانش گرفته بود

d. حسین چیزی در موردِ همسرِ مهربان و فداکارش فهمید که خواب را از چشمانش گرفته بود

حسین	نکته ای	در مورد	همسر	مهربان	و	فداکارش	فهمید
Hossein	noktei	dar morede	hamsar-e	mehraban	va	fadakar-ash	fahmid
Hossein	a point	about	wife	kind	and	devoted	understood
Proper name	indef. Noun	prep	noun	adj	connector	adj	heavy verb
که	خواب	را	از	چشمانش	گرفته بود		

ke	khab	ra	az	chashmanash	gerefteh bood		
that	sleep	ra	from	his eyes	had gotten		
relative marker	noun	accus. Marker	prep	noun+possessive adj	verb		

35

a. نازنین تصمیمی در موردِ خودش گرفت و همه‌ی تلاشش را به کار بست تا آن را اجرا کند

b. نازنین تصمیمی در موردِ آینده‌ی کاريِ خودش گرفت و همه‌ی تلاشش را به کار بست تا آن را اجرا کند

نازنین	تصمیمی	در مورد	آینده	کاری	خودش	گرفت	
Nazanin	tasmimi	dar morede	ayande-ye	kari-e	khodash	gereft	
Nazanin	a decision	about	future	job	himself	got	
Proper name	indef. Noun	prep	noun	adj	reflexive pronoun	light verb	
همه	تلاشش	را	کرد	تا	آن	را	اجرا کند
hameye	talashash	ra	kard	ta	an	ra	ejra konad
all (of)	his effert	ra	did	so that	that	ra	implemen t
noun	noun+possessive adj	accus. Marker	verb	coordinato r	demonst. Pronoun	accus. Marker	verb

c. نازنین واقعیتی در موردِ خودش فهمید که فکرش را حسابی مشغول کرد

d. نازنین واقعیتی در موردِ آینده‌ی کاريِ خودش فهمید که فکرش را حسابی مشغول کرد

نازنین	واقعیتی	در مورد	آینده	کاری	خودش	فهمید
nazanin	vagheiati	dar morede	ayande-ye	kari-e	khodash	fahmid
nazanin	a truth	about	future	job	himself	understood
Proper name	indef. Noun	prep	noun	adj	reflexive pronoun	heavy verb
فکرش	را	حسابی	مشغول	کرده بود		
fekrash	ra	hesabi	mashghool	kardeh bood		
his mind	ra	so much	busy	had done		
noun+possessive adj	accus. Marker	quantifier	adj	verb		

36

a. مهدی اعترافی در دادگاه کرد که به ضررِ خودش تمام شد

b. مهدی اعترافی در دادگاهِ علنيِ دیروز کرد که به ضررِ خودش تمام شد

مهدی	اعترافی	در	دادگاهِ	علني	دیروز	کرد
Mahdi	eterafi	dar	dadgah-e	alani-e	dirooz	kard
Mehdi	a confession	at	court	public	yesterday	did
Proper name	indef. Noun	prep	noun	adj	adverb	light verb
که	به ضرر	خودش	تمام شد			
ke	be zarar-e	khodash	tamam shod			
that	against	himself	was ended			
relative	prep	reflexive	verb			

marker		pronoun				

c. مهدی شهادت‌نامه‌ای در دادگاه نوشت که به ضررِ خودش تمام شد

d. مهدی شهادت‌نامه‌ای در دادگاهِ علنيِ دیروز نوشت که به ضررِ خودش تمام شد

مهدی	شهادت نامه ای	در	دادگاه	علنی	دیروز	نوشت
Mehdi	shahadat-nameh-i	dar	dadgah'e	alani-e	dirooz	nevesht
Mehdi	a testimony	at	court	public	yesterday	wrote
Proper name	indef. Noun	prep	noun	adj	adverb	heavy verb
که	به ضرر	خودش	تمام شد			
ke	be zarar-e	khodash	tamam shod			
that	against	himself	was ended			
relative marker	prep	reflexive pronoun	verb			
