# Supplementary material for: Dependency Resolution Difficulty Increases with Distance in Persian Separable Complex Predicates: Evidence for Expectation and Memory-Based Accounts
Source: Front Psychol. 2016 Mar 30;7:403. doi: 10.3389/fpsyg.2016.00403 (PMC4812816; doi:10.3389/fpsyg.2016.00403)
Supplement: Supplementary file 1 [file DataSheet1.zip › SafaviEtAl2016DataCode/items_fillers/filler.docx]

1. من و بهاره دیروز با هم به مدرسه رفتیم و سرِ کلاس خوابمان برد
2. من که خیلی گرسنه بودم از مغازه‏ای که نزدیکِ_خانه بود یک ساندویچ سوسیس خریدم
3. دلم می‏خواست باتمامِ_وجود برایِ دخترِ همسایه گریه_کنم آخر دیروز پدرش_را از_ دست_داد
4. بالاخره داریوش تصمیم_گرفت ماشینش_را بفروشد و یک دوچرخه‏ بخرد
5. دیشب مهمانیِ دوستِ_خواهرم خیلی خوش_گذشت اما جایِ_تو خالی_بود
6. آیدا باید همه‏ی لباس‏هایش_را می‏شست چون لباسِ تمیزی برای_کارش نداشت
7. احمد به_امید فوتِ پدرش_را تسلیت گفت و به مراسمِ خاکسپاری رفت
8. من دیروز همه‏ی کلوچه‏ها_را خوردم با_اینکه قبلش ناهارِ مفصلی_هم خورده_بودم
9. دیروز با_کمکِ همسایه دیوارِ حیاط_را رنگ_کردم چون خیلی_سال بود که رنگ نخورده_بود
10. اگر از دایی ساناز بخواهم حتما کمکم_می‏کند چون خیلی مردِ مهربانی است
11. من در کودکی خیلی شیطنت می‏کردم و از دیوارِ_راست بالا می‏رفتم
12. چشم‏های فریبا قرمز شده_بود اما به_روی خودش نمی‏آورد
13. پریناز دیشب به جشنِ تولدِ دوستش رفته_بود و دیر_وقت برگشت
14. از_وقتی که پروین رفت، دیگر این_خانه رنگِ آرامش به_خود ندید
15. وقتی گفتم_که می‏خواهم برگردم همه شوکه شده_بودند
16. من از خودِ معلمم شنیدم_که می‏گفت امسال امتحانات سخت‏تر از سالِ گذشته بود
17. تو باید بدانی_که این_خانه مقرراتی دارد و باید به_آن احترام بگذاری
18. دکتر به_من گفت_که باید در_خانه استراحت کنم و مایعاتِ زیادی بنوشم
19. من از_هواپیما می‏ترسیدم اما وقتی سوار_شدم ترسم ریخت
20. خانه‏ی مادربزرگم خیلی قدیمی است اما حیاطِ بزرگی دارد که پر_از گل‏های اطلسی است
21. پدرِ کیوان با_وجود_اینکه ورشکست شد هیچ_وقت دست_از تلاش برنداشت
22. دیروز در_مدرسه به سئوالی_که معلم پرسید جواب_دادم و معلمم حسابی تشویقم کرد
23. همکلاسیِ من خیلی درس_می‏خواند اما هیچ_وقت نمره‏ی بالایی نمی‏گرفت
24. غزاله دیروز ساعت‏ها رویِ نیمکتِ پارک نشست و فکر_کرد
25. زهره پارسال در_دانشگاهِ آزاد قبول_شد اما نرفت چون نمی‏توانست شهریه‏اش_را بپردازد
26. من دیشب دوستانم_را به_خانه دعوت_کردم و یک غذایِ خوشمزه پختم
27. افسانه هیچ_وقت از_چیزی شکایت نمی‏کرد اما دیروز حسابی صدایش درآمده_بود
28. احسان تصمیم_گرفت که به_جایِ دانشگاه به_سربازی برود و رفت
29. تو خوب می‏دانی که من از_چه رفتاری بدم_می‏آید اما باز هم آن_را تکرار می‏کنی
30. دیشب از_خواب پریدم و دیگر خوابم نبرد
31. من دیروز هیچ_لباسی نخریدم اگرچه حسابی گشته_بودم
32. داوود دیروز آنقدر خسته_بود که نتوانست به سرِ_کار برود و در_خانه ماند
33. الهه که_داشت بال_درمی‏آورد به_من گفت که دانشگاه قبول شده_است
34. دیشب از خانه‏ی دخترخاله‏ام که برمی‏گشتم کیفم را دزدیدند
35. من به رستورانِ همیشگی رفتم اما تعطیل بود
36. پروانه به_خاطرِ_من تلویزیون_را خاموش_کرد تا بیدار نشوم
37. سهیل چند_وقت_پیش به_من گفت_که می‏خواهد با_پدرش در_این_مورد حرف بزند
38. وقتی پیمان برایم آهنگ_زد اشک در_چشمانم حلقه زده_بود
39. مهرداد آنقدر تلاش کرد تا بالاخره به_جایگاهی_که می‏خواست رسید
40. دوستِ_من که در_پاریس زندگی می‏کند دیروز به_من تلفن_زد
41. شبی_که فرزانه_را دیدم بارانِ شدیدی می‏بارید و چترم_را فراموش کرده_بودم
42. من در_کودکی خیلی فوتبال دوست_داشتم و خوب_هم بازی می‏کردم
43. دیروز_که از_مدرسه برگشتم خیلی گرسنه بودم اما خبری از_غذا نبود
44. میلاد تمامِ_روز_را رانندگی کرد و خیلی خسته شد
45. من دیروز زود به_خانه برگشتم اما کلیدم_را جا_گذاشته_بودم
46. بیژن رادیو_را روشن_کرد ولی برنامه‏ی موردِ علاقه‏اش تمام شده_بود
47. شهاب_و_آوا دیروز از_شمال برگشتند و به_خانه‏ی خواهرِ آوا رفتند
48. من از_میوه‏ای_که خیلی رسیده¬باشد بدم_میامد و لب نمی‏زدم
49. فرید از_من به_خاطرِ اتفاقِ دیروز معذرت_خواست و من_هم او_را بخشیدم
50. من که همیشه از_ارتفاع می‏ترسیدم دیروز با_دوستانم به_کوه رفتم
51. محمود به قصابی رفت و برایِ مهمانیِ امروز دو_کیلو جوجه خرید
52. شیما که_حسابی گیج شده_بود از_من پرسید که ساعت چند_است
53. من بعد_از سالها مونا_را در_تاکسی دیدم و او_را شناختم
54. سعید نصیحتی به_من کرد که هرگز فراموش_نمی‏کنم
55. آزاده دیشب آنقدر غذا خورده_بود که دل_درد گرفت
56. محمد به_زهرا انگشتری هدیه_داد اگرچه اصل نبود
57. من از_شوخیِ دیروزِ سحر خوشم_نیامد و بیرون رفتم
58. من دیروز با_شریکم قرار_گذاشتم که در_موردِ پروژه صحبت_کنم
59. هانیه بالاخره متوجه_شد که در_موردِ نوید اشتباه می‏کرده_است
60. ابراهیم بعد_از اینکه به_اصفهان رفت کارِ جدیدی_را شروع_کرد
61. برادرم که از_من بزرگتر بود همیشه مرا حمایت می‏کرد
62. سیامک هفته‏ی_پیش رضایت_داد که روزبه از_زندان آزاد شود و دیه نپردازد
63. ژاله برای_من یک کلاهِ_گرم بافت که خیلی خوشرنگ بود
64. فهیمه دو هفته_پیش مریض شد و مرخصیِ استعلاجی گرفت
65. سهراب با_من تماس گرفت و همه_چیز_را توضیح داد
66. من دیروز یک_جفت کفشِ نو برای_خودم خریدم که خیلی گران بود
67. شایان پارسال در_مسابقه‏ی شطرنج برنده شد، با اینکه زیاد تمرین نکرده_بود
68. بابک دیروز به_من کمک کرد با_وجود_اینکه سرش خیلی شلوغ بود
69. شیوا دیروز سوپ خوشمزه‏ای درست کرده_بود که مثلِ همیشه خیلی چسبید
70. پدرام وقتی خسته شد به_یک آهنگِ جدید گوش_داد
71. من از_عطری_که لاله برایم هدیه گرفته_بود خوشم نیامد اما به_روی خودم نیاوردم
72. مدیرِ شرکت دیروز از_شدتِ ناراحتی استعفا_داد و از_اتاق بیرون رفت
73. من برای تولدِ خواهرزاده‏ام یک_ماشینِ کنترلی خریدم که خیلی پیشرفته بود
74. رامین نمی‏توانست جلوی خودش_را بگیرد و زد زیرِ خنده
75. مرجان که از_رفتارِ بهروز حسابی شاکی بود با_او چند_روزی قهر_کرد
76. پدربزرگم آن_درخت_را خیلی دوست داشت و نمی‏گذاشت کسی از_آن بالا_برود
77. من دیروز از_صبح تا شب درس خواندم که برای امتحان آماده باشم
78. آرزو هفته‏ی پیش یک بچه_گربه پیدا_کرد و آن_را به_خانه آورد
79. مژده از_سفر که برگشت بلافاصله کارش_را شروع کرد
80. رئیس به_من مرخصی داد که بتوانم به_کارهای عقب‏مانده‏ام برسم
81. دخترم که پنج_سالش است دیروز برای_تولدم یک_نقاشی کشید
82. هومن نمی‏خواست پیشنهادِ همکارش_را قبول کند اما بالاخره مجبور شد
83. سوگل از_اینکه نتوانستم به جشنِ عروسیِ_او بروم دلخور شد
84. اشکان پارسال شروع_کرد که یک_سازِ جدید یاد بگیرد
85. پرستو که خیلی عصبانی بود در_را پشتِ_سرش به_هم کوبید
86. فرشته نتوانست جلوی احساساتش_را بگیرد و بغضش ترکید
87. سروش پارسال ماشینش_را عوض کرد و یک ماشینِ مدل_جدید خرید
88. پارسا شبِ قبل_از عروسی تصادف کرد و پایش شکست
89. سیاوش نمی‏خواست که من_از موضوع سر_دربیاورم و ناراحت شوم
90. دیشب_که خوابم نمی‏برد به_یاسمن تلفن_زدم و با_هم کلی حرف زدیم
91. من از_روزی_که فارغ‏التحصیل شدم دیگر همکلاسی‏هایم_را ندیدم
92. نسرین دیروز دیر از_سرِ_کار برگشت و خیلی خسته بود
93. مهشید که_خیلی شوکه شده_بود نمی‏توانست حرف بزند
94. خاله_نرگس وقتی دیروز سرزده به_خانه‏ی_ما آمد خیلی ذوق کردیم
95. من به_هنگامه پولی_که احتیاج داشت را قرض دادم اما او هنوز به_من پس_نداده‏است
96. خواهرم دیشب یک_کیکِ شکلاتی برایم درست_کرد که خیلی مزه_داد
97. مازیار هفته‏ی_پیش به_من گفت_که می‏خواهد به_شیراز برود
98. روشنک که_دخترِ زیبایی است اصلا اعتماد_به_نفسِ خوبی ندارد
99. سامان دیروز به_بیمارستان رفت تا چشمش_را عمل_کند
100. آرش دیشب از_من ماشینم_را قرض گرفت تا به_مهمانی برود
