# Supplementary material for: Dependency Resolution Difficulty Increases with Distance in Persian Separable Complex Predicates: Evidence for Expectation and Memory-Based Accounts
Source: Front Psychol. 2016 Mar 30;7:403. doi: 10.3389/fpsyg.2016.00403 (PMC4812816; doi:10.3389/fpsyg.2016.00403)
Supplement: Supplementary file 1 [file DataSheet1.zip › SafaviEtAl2016DataCode/items_fillers/instructions.pdf]

Instruction of the norming tests, SPR and ET

Sentence completion :

Eight type of forms (4 latin-square lists for two experiments) were distributed via email. Here is the instruction explained in the beginning of the form (see examples here https://www.dropbox.com/sh/uhxmsh4ebb6ybvp/AADy0qpVHmSsP3IanEey19kKa?dl=0) :

از همکاری شما در بخشی از پروژه روان شناسی زبان بسیار متشکریم .لطفا با نوشتن اولین و" ساده ترین "واژه/دو واژه ای که به ذهنتان می رسد) در جای تعیین شده جملات زیر را کامل کنید .حداکثر دو واژه .نکته مهم در انجام آزمون این است که آن را به تنهایی، بدون مشورت با دیگری و با تمرکز کافی انجام دهید .

را فعال کنید .لطفا اسم فایل را* “Enable Editing” در صورتی که نمی توانید در این صفحه تایپ کنید، گزینه تغییر ندهید .

Thanks for your cooperation in this Psycholinguistic project. Please complete the sentences (in the related boxes) with the first word/words that come(s) to your mind. It is important to do this test alone without anybody else's help and with enough concentration.

Acceptabbility

The participants filled in this online google form and the results were then converted to a dataframe. https://docs.google.com/forms/d/1gYmv5vDoEwyb-BRR2PeajYVcJcXoRkQbIAguQsLewGY/viewform

Here is the instruction explained on top of the form (followed by English translation):

لطفا جملات زیر را با دقت و تمرکز کافی بخوانید و بر اساس اینکه تا چه اندازه آن را در فارسی محاوره ای امروز می شنوید و قابل قبول است از 1 تا 7 رتبه بندی کنید .لازم به ذکر است که واژه ها در برخی از این جملات لزوما بر اساس تعاریف کتاب های دستور کلاسیک زبان فارسی چیده نشده اند .بنابراین برخی جملات ممکن است در ایده آل ترین حالت نوشته نشده باشند اما باز هم کاملا قابل قبول باشند زیرا به طور گسترده در زبان روزمره به کار می روند) حتی اگر شما شخصا هرگز ساختار مورد نظر را به این شکل به کار نبرید .(

این آزمون حدود 10 دقیقه به طول می انجامد و در پایان با کلیک بر روی گزینه ی ثبت (submit) پاسخ های شما در فضای گوگل ثبت می شود .برای محفوظ ماندن اطلاعات شخصی شما) نام و نام خانوادگی ، سن ، مقطع و رشته تحصیلی، زبان مادری و زبان های دیگر (در فضای مجازی ، این اطلاعات همراه با فرم ثبت نمی شود اما از آنجایی که این اطلاعات برای تحلیل جامعه آماری ضروری است ، خواهشمندیم بلافاصله پس از ثبت پاسخ، این مشخصات را به طور خصوصی به ایمیل farnoosh2000ir@gmail.com ارسال نمایید .این اطلاعات نزد آزمونگر محفوظ می ماند و در صورت نیاز، به صورت گمنام) تنها با اختصار نام و نام خانوادگی(در تحلیل نتایج مقاله ذکر می شود .

Please read the following sentences carefully and with enough concentration and rate them from 1 to 7 based the level of acceptability in today's Persian. It is worth mentioning that these sentences may not all be in their most ideal and common form (according to classic textbooks) but they can still be acceptable as they are widely used by native speakers in everyday life (even if you do not personally use this form).

The current test takes approximately 10 minutes and at the end your responses will be recorded after clicking on "submit" button. In order to keep your personal data confidential, we ask you to send us your information (name,age, education, mother tongue and other languages) to this email () immediately after filling in the form. This information will be saved and used anonymously (only with initials) in our research.

SPR

Here is the instruction of the self-paced reading experiments (followed by English translation):

باید شما و میشود ظاهر صفحه روی کلمه یک * کلیدِ زدن با که اینصورت به میخوانید کلمه به کلمه را جمـــلاتی شما آزمون این در
یک کل تا میکند پیدا ادامه اینکار .شود قبلی کلمهء جایگزن جدید کلمهء تا بزنید را * کلید دوباره کلمه آن خواندن از بعد بلافاصله
با لطفا .میشود داده نـمایش صفحه روی خبری عبارت یک غالب در مفهومی سوال یک جمله هر پایان از بعد .بـخوانید را جـمله
کنید رد یا و تایید را خبری عبارت آن دُرسـتی دکمه یه زدن با اید خوانده که ای جـمله مفهوم به توجه

In this test, you will read some sentences word by word such that each word will appear after clicking a button on keyboard and a new word will be replaced when you enter again. Continue this untill you read the whole sentence. After the end of each sentence, a comprehension question in form of a sentence will be displayed. Please according to the content of the sentence you had read, click on the right or wrong button.

****** The right/wrong buttons were marked on the keyboard by small papers*******

در صورتی که هر سوالی دارید میتوانید اکنون از آزمایشـگر بپرسـید و اگر سوالی ندارید ابتدا شـیوهء کار را با چند جـمله تـمرین میکنیم

If you have any questions, you can ask now from the examiner. If not, let's practice with a few sentences.

صـورت این غیر در بپرسید آزمایشـگر از لطفا دارید پُرسـشی اگر شــوید آشنا آزمون نـحوهء با شما تا بودند تـمرینی جـمــلات اینها
کنید شــروع را اصلی آزمون میتوانید

These were the practice sentences so that you get familiar with the procedure of this study. If you have any questions, please ask the examiner. If not, you can proceed to the main test.

Eye-tracking

Here is the instruction of the eye-tracking experiments (followed by English translation):

لطفا جملات را با سرعت طبیعی بخوانید و بعد به گوشه سمت چپ مانیتور نگاه کنید تا جمله ای در رابطه به جمله قبلی پخش شود .
در صورتی جمله جدید در رابطه با جمله قبلی درست بود کلید سمت راست و در صورتی که اشتباه بود کلید سمت چپ را فشار دهید.

لطفا سعی کنید زمانی که جمله سئوالی پخش می شود پلک بزنید و حرکات بدن و صورت خود را به حداقل برسانید در صورتی که هر سوالی دارید میتوانید اکنون از آزمایشگر بپرسید و اگر سوالی ندارید ابتدا شیوهء کار را با چند جمله تمرین می کنیم!

Please read the sentences with a natural pace and then look at the left buttom corner of the monitor so that a sentence related to the previous sentence appears. If this new sentence is right, click right and if it is wrong, click left.

Please try not to blink when the main sentence is being presented and to minimize your body and face movements. If you have any questions, you can now ask the examiner. If not, let's practice with a few sentences.

صورت این غیر در بپرسید آزمایشگر از لطفا دارید پُرسشی اگر شوید آشنا آزمون نحوهء با شما تا بودند تمرینی جملات اینها کنید شروع را اصلی آزمون میتوانید

These were the practice sentences so that you get familiar with the procedure of this study. If you have any questions, please ask the examiner. If not, you can proceed to the main test.
