# Supplementary material for: Dependency Resolution Difficulty Increases with Distance in Persian Separable Complex Predicates: Evidence for Expectation and Memory-Based Accounts
Source: Front Psychol. 2016 Mar 30;7:403. doi: 10.3389/fpsyg.2016.00403 (PMC4812816; doi:10.3389/fpsyg.2016.00403)
Supplement: Supplementary file 1 [file DataSheet1.zip › SafaviEtAl2016DataCode/Pretests/Pretest-2-acceptability-rating(sep-vs-insep)/Acceptability-rating.docx]

Acceptability rating

<https://docs.google.com/forms/d/1gYmv5vDoEwyb-BRR2PeajYVcJcXoRkQbIAguQsLewGY/viewform>
