# Supplementary material for: Dependency Resolution Difficulty Increases with Distance in Persian Separable Complex Predicates: Evidence for Expectation and Memory-Based Accounts
Source: Front Psychol. 2016 Mar 30;7:403. doi: 10.3389/fpsyg.2016.00403 (PMC4812816; doi:10.3389/fpsyg.2016.00403)
Supplement: Supplementary file 1 [file DataSheet1.zip › SafaviEtAl2016DataCode/READMELongPP/Expt2-intervener.docx]

id type

1 PP1

2 PP1

3 PP1

4 PP1

5 PP2

6 PP2

7 PP2

8 PP2

9 PP2

10 PP1

11 PP2

12 PP1

13 PP2

14 PP2

15 PP2

16 PP1

17 PP2

18 PP2

19 PP1

20 PP2

21 PP2

22 PP2

23 PP2

24 PP2

25 PP2

26 PP2

27 PP2

28 PP2

29 PP1

30 PP2

31 PP2

32 PP2

33 PP2

34 PP2

35 PP2

36 PP2
