# Supplementary material for: Dependency Resolution Difficulty Increases with Distance in Persian Separable Complex Predicates: Evidence for Expectation and Memory-Based Accounts
Source: Front Psychol. 2016 Mar 30;7:403. doi: 10.3389/fpsyg.2016.00403 (PMC4812816; doi:10.3389/fpsyg.2016.00403)
Supplement: Supplementary file 1 [file DataSheet1.zip › SafaviEtAl2016DataCode/READMELongPP/Expt2-PPtype.pdf]

The type of long intervener (PP) in experiment 2:

PP1

Red: (N-ez N-ez N/pronoun/proper name) OR [N-ez [N-ez N]]

PP2

Blue: (N-ez adj-ez N/pronoun/proper name) → adj. is attributed to the **first** N/pron/proper name OR [[N-ez adj-ez] N]

Green: (N-ez N-ez adj) → adj. is attributed to the **second** N/pron/proper name OR [N-ez [N-ez adj]]

Orange: (N adj-ez adj) → both adjectives are attributed to the noun OR [N-ez [adj-ez adj]]

Purple: (superlative adj N N/pronoun/proper name) → the superlative adj. is attributed to the first N OR [adj [N-ez pronoun]]

1. khahare dooste man (N-ez N-ez pronoun)
2. Jashne tavallode man (N-ez N-ez pronoun)
3. Dokhtarkhaleye dooste man (N-ez N-ez pronoun)
4. Khaharzadeye hamsayeye Mina (N-ez N-ez proper name)
5. Hamsayeye mahalle ghadim (N-ez N-ez adj)
6. Harfe marde bikhaneman (N-ez N-ez adj)
7. Maghaleye akhire Chomskey (N-ez adj-ez Proper name)
8. Pedare varshekasteye ma (N-ez adj-ez pronoun)
9. Sherkate tejari bazargani (N-ez adj adj)
10. Hamkelasie baradare mahsa (N-ez N-ez proper noun)
11. Lebase ghermeze jadid (N-ez adj-ez adj)
12. Khaharzadeye hamotaghie man (N-ez N-ez pronoun)
13. Pedare bimare man (N-ez adj-ez pronoun)
14. Nazdiktarin dooste man (superlative adj N-ez pronoun)
15. Zendegie kesalat-bare man (N-ez adj-ez pronoun)
16. Ostade zabane man (N-ez N-ez pronoun)
17. Shagerdane sale akhar (N-ez N-ez adj)
18. Baradare afsordeye man (N-ez adj-ez pronoun)
19. Pesarkhaleye dooste man (N-ez N-ez pronoun)
20. Daneshjooye momtaze khod (N-ez adj-ez pronoun)
21. Pedare salkhordeye doost (N-ez adj-ez N)
22. Shohare khahare bozorg (N-ez N-ez adj)
23. Khanevadeye koodake roboode-shode (N-ez N-ez adj)
24. Khahare mehrabane man (N-ez adj-ez pronoun)
25. Dokhtare koochake man (N-ez adj-ez pronoun)

26. Madreseye dolatie Marefat (N-ez adj-ez proper name)
27. Baradare javane man (N-ez adj-ez pronoun)
28. Shagerde ghadimie khod (N-ez adj-ez pronoun)
29. Baradare doost-e Neda (N-ez N-ez proper name)
30. Gozashteye mobhame Setare (N-ez adj-ez proper name)
31. Khahare koochektare man (N-ez adj-ez pronoun)
32. Khahare bozorge Parisa (N-ez adj-ez proper name)
33. Masaleye malie sherkat (N-ez adj-ez N)
34. Hamsare mehrabane fadakar (N-ez adj-ez adj)
35. Ayandeye karie khod (N-ez adj-ez pronoun)
36. Dadgahe alanie diruz (N-ez adj-ez N)
