# Supplementary material for: Dependency Resolution Difficulty Increases with Distance in Persian Separable Complex Predicates: Evidence for Expectation and Memory-Based Accounts
Source: Front Psychol. 2016 Mar 30;7:403. doi: 10.3389/fpsyg.2016.00403 (PMC4812816; doi:10.3389/fpsyg.2016.00403)
Supplement: Supplementary file 1 [file DataSheet1.zip › SafaviEtAl2016DataCode/READMELongPP/SamvelianEzafe2007.pdf]

CAMBRIDGE
UNIVERSITY PRESS

A (Phrasal) Affix Analysis of the Persian Ezafe
Author(s): Pollet Samvelian
Source: *Journal of Linguistics*, Vol. 43, No. 3 (Nov., 2007), pp. 605-645
Published by: Cambridge University Press
Stable URL: http://www.jstor.org/stable/40058014
Accessed: 19-11-2015 22:53 UTC

Your use of the JSTOR archive indicates your acceptance of the Terms & Conditions of Use, available at http://www.jstor.org/page/info/about/policies/terms.jsp

JSTOR is a not-for-profit service that helps scholars, researchers, and students discover, use, and build upon a wide range of content in a trusted digital archive. We use information technology and tools to increase productivity and facilitate new forms of scholarship. For more information about JSTOR, please contact support@jstor.org.

Cambridge University Press is collaborating with JSTOR to digitize, preserve and extend access to *Journal of Linguistics*.

http://www.jstor.org

This content downloaded from 143.167.2.135 on Thu, 19 Nov 2015 22:53:22 UTC
All use subject to JSTOR Terms and Conditions

J. Linguistics **43** (2007), 605–645. © 2007 Cambridge University Press
doi:10.1017/S0022226707004781 Printed in the United Kingdom

A (phrasal) affix analysis of the Persian Ezafe[1]

POLLET SAMVELIAN

Université de la Sorbonne Nouvelle – Paris III

(Received 21 April 2006; revised 10 May 2007)

This paper discusses the status of the Ezafe particle -(*y*)*e* in Persian and provides an affixal analysis of the Ezafe, formalized within Head-driven Phrase Structure Grammar (HPSG). The Ezafe, a feature of certain Western Iranian languages, is realized as an enclitic and links the head noun to its modifiers and to the possessor NP. The latter follow the head and are linked to one another by the Ezafe. On the basis of crucial empirical facts that have never been discussed in previous studies, I argue that the Ezafe is best regarded as an affix attaching to nominal heads (nouns, adjectives and some prepositions), as well as to nominal intermediate projections, and marking them as expecting a modifier or a direct nominal complement. Viewed as such, the Ezafe construction is an instance of the head-marked pattern of morphological marking of grammatical relations. This analysis differs from all previous accounts of the Ezafe (i.e. as case-marker, syntactic or phonological linker) and entails that the Ezafe, which originated in the Old Iranian relative particle -*hya*, has undergone a process of reanalysis-grammaticalization, to end up as a part of nominal morphology.

1. Introduction

The Ezafe, from Arabic *iḍāfa* 'addition, adjunction', is a feature of a number of West Iranian languages (Windfuhr 1989), e.g. Persian, Kurdish dialects, Hawramani, Zazaki, etc., which share several aspects of their noun phrase structure: a) The surface word order pattern within the NP is strongly head-initial; b) Adjectival modifiers, the possessor NP, prepositional phrases and the relative clause follow the head noun; c) All elements occurring between the head noun and the possessor NP are linked to the head noun and to one

[1] I am especially indebted to Anne Abeillé, Karen Ferret and Jesse Tseng for their very helpful comments and suggestions on an earlier version of this paper. I also want to thank Amr Ahmed, Farzaneh Deravi, Justine Landau, Jean-Marie Marandin, Cédric Patin and Martine Vertalier, as well as audiences at the First International Conference on Aspects of Iranian Linguistics (2005) and TYPO4 Conference (2004). Last but not least, special thanks to the three anonymous *Journal of Linguistics* referees for their detailed and stimulating comments and suggestions.

This content downloaded from 143.167.2.135 on Thu, 19 Nov 2015 22:53:22 UTC
All use subject to JSTOR Terms and Conditions

another by the Ezafe, which is an enclitic. The schematic structure for the Persian NP, for instance, is as follows:[2]

(1) (Det) N-EZ AP-EZ PP-EZ NP(Poss)
in ketâb-e kohne-ye bi arzeš-e maryam
this book-EZ ancient-EZ without value-EZ Maryam
'this ancient worthless book of Maryam's'

Since Samiian (1983), who pointed out some puzzling restrictions on the Ezafe construction in Persian, the latter has been a particular focus of interest in several studies (see Samiian 1983, Samiian 1994, Ghomeshi 1997b, Schroeder 1999, Kahnemuyipour 2000, Schroeder 2002, Holmberg & Odden 2005, Larson & Yamakido 2005, among others) and different analyses have been suggested to account for its peculiarities in the Iranian languages.

In this paper, I especially discuss the status of the Persian Ezafe. Relying on crucial empirical facts that have never been taken into account in previous studies, I will advocate a treatment of the Ezafe as an affix attaching to nominal heads and intermediate nominal projections before the head combines with its single direct complement, and marking them as expecting a modifier or an NP complement. Viewed as such, the Ezafe construction is a special instance of the head-marked pattern of morphological marking of grammatical relations (Nichols 1986) in that a nominal head and its intermediate projections are morphologically marked to receive a dependent.

The analysis outlined in this paper differs from that of Ghomeshi (1997b), who considers that the Ezafe has no morphological status and is a sort of 'phonological linker' inserted in PF to indicate phrasing, because of the non-projecting properties of Persian nouns. It also differs from the case-marker analysis of the Ezafe proposed by Samiian (1994) and Larson & Yamakido (2005).

The origins of the Ezafe in Modern Persian can be traced back to the Old Persian relative pronoun/demonstrative *hya* (*hyâ*, *tya*) (see Haider & Zwanziger 1981, Darmesteter 1883, Kent 1944, among others), which was used to introduce relative clauses and, to a lesser extent, attributive adjectives and the possessor NP. At the intermediate stage of Middle Persian, the particle evolved into *y* or *i*, and progressively ceased to function as a relative pronoun, becoming specialized as a device for nominal attribution. The analysis outlined in this paper further entails that the Ezafe particle has

[2] Abbreviations: COP = copula, CPR = comparative, DEF = definite, DEM = demonstrative, EZ = ezafe, FEM = feminine, INDEF = indefinite, INF = infinitive, MA = mood-aspect, MASC = masculine, OBL = oblique, PAF = personal affix, PAS = past, PL = plural, PPL = past participle, PRES = present, RESTR = restrictive, RRC = reduced relative clause, SG = singular.

This content downloaded from 143.167.2.135 on Thu, 19 Nov 2015 22:53:22 UTC
All use subject to JSTOR Terms and Conditions

undergone a process of reanalysis-grammaticalization, being thus reinterpreted as a part of nominal (phrasal) inflection.

The paper is organized as follows. The next section includes a brief presentation of the Ezafe construction. The third section is devoted to a critical survey of two major studies on the Ezafe in Persian, Samiian (1983) and Ghomeshi (1997b). Samiian (1983) is the most detailed contribution on the topic: almost all the empirical generalizations stated in this work, particularly the restrictions on the expansion of the constituents occurring within the Ezafe domain, have been taken up in subsequent studies. Ghomeshi's analysis, building on Samiian's data, has some important theoretical consequences for the structure of noun phrases in Persian, since the peculiarities of the Ezafe construction are assumed to follow from the inability of Persian nouns to give rise to a maximal projection. It will first be shown, on the basis of numerous attested examples, that not all of the restrictions mentioned by Samiian stand up to close inspection. Consequently, it will be argued that Ghomeshi's analysis, which is based essentially on Samiian's data, faces serious empirical and conceptual problems. I will also present some conclusive evidence against viewing the Ezafe as a case-marker, as suggested by Samiian (1994) and Larson & Yamakido (2005).

In the fourth section, on the basis of the diagnostic criteria suggested by Zwicky & Pullum (1983), Zwicky (1987) and Miller (1992), it will be argued that the Ezafe is best regarded as an affix whose function is to indicate dependency relations between the head noun, its modifiers and the possessor NP. The affixal analysis will also be applied to the definite and indefinite articles and to personal enclitics occurring within the NP. It will be shown that different restrictions on the Ezafe construction follow from the Ezafe's morphological status.

In the last section, after discussing different technical treatments proposed for phrasal affixes in the literature, a lexicalist approach will be adopted, in line with Zwicky (1987) and Miller (1992), and according to which phrasal affixes are realized by the same device as word-level inflectional affixes. Finally, a formal treatment, couched within Head-driven Phrase Structure Grammar (HPSG) (Pollard & Sag 1994, Ginzburg & Sag 2000), and appealing to Edge Features as worked out by Miller (1992) and Tseng (2003), will conclude the paper.

2. The Ezafe construction: an overview

Western Iranian languages display two different patterns with regard to word order within the NP, head-final or head-initial. The second pattern generally involves the Ezafe construction. Modifiers and the possessor NP are linked to one another and to the head noun by the Ezafe, which is

This content downloaded from 143.167.2.135 on Thu, 19 Nov 2015 22:53:22 UTC
All use subject to JSTOR Terms and Conditions

realized as an enclitic vowel in Persian (/e/ after consonants and after /i/, and /ye/ after other vowels):

(2) (a) lebâs-e arusi /zibâ /bi âstin
dress-EZ wedding beautiful without sleeve
'wedding dress' / 'beautiful dress' / 'dress without sleeves'
(b) lebâs-e maryam
dress-EZ Maryam
'Maryam's dress'
(c) lebâs-e arusi-e zibâ-ye bi âstin-e maryam
dress-EZ wedding-EZ beautiful-EZ without sleeve-EZ Maryam
'Maryam's beautiful wedding dress without sleeves'

As shown by (2a), the Ezafe introduces different kind of modifiers: attributive nouns, adjectival and prepositional phrases. It also occurs on the head noun in case the latter is followed by the Possessor NP, as illustrated by (2b). Finally, it may appear more than once in the same NP. However, not all of the dependents of a noun may be introduced by the Ezafe. Prepositional arguments of the head noun, for instance, are merely juxtaposed to it. When the noun is followed either by modifiers or by a possessor NP, prepositional arguments are placed outside and following the Ezafe domain:

(3) (a) bahs(*-e) bâ omid
discussion(-EZ) with Omid
'discussion with Omid'
(b) bahs-e diruz-e maryam(*-e) bâ omid
discussion-EZ yesterday-EZ Maryam(-EZ) with Omid
'Maryam's yesterday's discussion with Omid'

Likewise, relative clauses are never attached to the head noun by the Ezafe:

(4) mard-i(*-e) ke vâred šod
man-RESTR(-EZ) that entered become.PAS
'the man who entered'

Demonstratives, quantifiers, numerals and superlative adjectives precede the head noun and do not take the Ezafe:

(5) in(*-e) /behtarin(-*e) ketâb
this(-EZ) best(-EZ) book
'this book' / 'the best book'

A selected class of attributive adjectives may also precede the head noun, but this order is generally attested in poetry or in lexicalized cases. Here too the Ezafe does not occur:

(6) zibâ(*-ye) doxtar
beautiful(-EZ) girl
'beautiful girl'

This content downloaded from 143.167.2.135 on Thu, 19 Nov 2015 22:53:22 UTC
All use subject to JSTOR Terms and Conditions

The Ezafe construction is not restricted to NPs. Within adjectival phrases, the Ezafe may link the adjectival head to its single complement:

(7) negarân-e maryam
worry-EZ Maryam
'worried about Maryam'

The Ezafe can also occur on some prepositional heads. Samiian (1994) establishes three classes of prepositions in Persian: a) Prepositions that cannot take the Ezafe (P1 prepositions), e.g. *be maryam* 'to Maryam'; b) Prepositions that necessarily take the Ezafe (P2 prepositions), e.g. *zir-e miz* 'under the table'; c) Prepositions that optionally take the Ezafe (P3 prepositions), e.g. *ru*(*-ye*) *miz* 'on (the) table'.

It should be noted that from a historical point of view the class of prepositions properly speaking in Persian coincides with P1 prepositions. The members of the two other classes arose from nouns (e.g. *ru* 'face'), adverbs (e.g. *piš* 'near, by') or Arabic prepositions (e.g. *aleyh* 'against').

To sum up, the Ezafe occurs within NPs, APs and PPs, between the head and its NP complement, or, in the case of NPs, between the head noun and its modifiers.

3. A CRITICAL SURVEY OF SOME PREVIOUS STUDIES

Samiian (1983) is the first detailed study on the Ezafe in Persian within a modern syntactic framework, namely X-bar theory. The empirical facts mentioned by Samiian have been taken up in subsequent works, e.g. Ghomeshi (1997b) and Kahnemuyipour (2000), although they have been accounted for in a radically different way. In this section, I will first consider different restrictions on the Ezafe construction pointed out by Samiian. I will then give a detailed account of Ghomeshi (1997b) and will show that her analysis falls short in several respects. Finally, I will briefly consider the case-marker analysis of the Ezafe proposed by Samiian (1994) and Larson & Yamakido (2005).

3.1 *Restrictions on the Ezafe construction: Samiian (1983)*

Samiian (1983) points out two major types of restrictions on the Ezafe construction: the constituents occurring within the Ezafe domain are strictly ordered, and they are constrained with regard to their distribution. The elements linked by the Ezafe to the head noun occur in a fixed order:

(8)	ketâb-e	târix-e	sabz-e	bi	arzeš-e maryam
	book-EZ	history-EZ	green-EZ	without	value-EZ Maryam

'the green worthless history book of Maryam's'

This content downloaded from 143.167.2.135 on Thu, 19 Nov 2015 22:53:22 UTC
All use subject to JSTOR Terms and Conditions

The head noun is followed by an attributive noun, an adjective modifier, a prepositional modifier and a possessor NP in that order:

(9) Head N – Attributive N – Adj Mod – Prep Mod – Poss NP

Although Samiian considers this order to be a strict one, it must be noted that the only absolute constraint concerns the placement of the possessor NP, which must occur in the final position within the Ezafe domain. Within this domain, other elements linked by the Ezafe are by preference ordered as in (9) above, but they may also be ordered differently.

Samiian also notes some restrictions on the expansion of phrasal complements and modifiers within the Ezafe domain: 'The attributive noun phrase and the adjective phrase have to be head-final and the prepositional phrase of time and location cannot carry a sentential complement' (p. 40). According to Samiian, the only element that can be freely and recursively expanded is the possessor NP.

On the basis of the following example, Samiian concludes that attributive noun phrases surface only as bare nouns:

(10) kif-e čarm / *in čarm
bag-EZ leather this leather
'leather bag' / (putatively) 'a bag of this leather' (Samiian 1983: 46)

Adjectival modifiers cannot take either nominal, prepositional or sentential complements when occurring within the Ezafe construction.[3] These restrictions are exemplified by (11b), (12b) and (13b) respectively:

(11) (a) mard-e negarân-i vâred šod
man-EZ worried-INDEF entered become.PAS
'A worried man entered.'
(b) *mard-e negarân-e bačče-hâ-yaš-i vâred šod
man-EZ worried-EZ child-PL-PAF.3.SG-INDEF entered become.PAS
(putatively) 'A man worried about his children entered.'

(12) (a) mardom-e xašmgin-e tehrân be-pâ xâst-and
people-EZ angry-EZ Tehran to-foot rise.PAS-3.PL
'The angry people of Tehran rose up.'
(b) *mardom-e xašmgin az erteja-ye tehrân be-pâ
people-EZ angry at reactionaries-EZ Tehran to-foot
xâst-and
rise.PAS-3.PL
(putatively) 'The people of Tehran angry at the reactionary forces rose up.'

[3] I do not agree with Samiian on this point. As we shall see further on, adjectives followed by a complement may be linked to the head noun by the Ezafe. Unlike Samiian, I consider example (12b) to be well-formed.

This content downloaded from 143.167.2.135 on Thu, 19 Nov 2015 22:53:22 UTC
All use subject to JSTOR Terms and Conditions

(13) (a) mardom-e xošhâl-e irân jašn gereft-and
people-EZ happy-EZ Iran feast take.PAS-3.PL
'The happy people of Iran celebrated.'
(b) *mardom-e xošhâl ke šâh kešvar râ tark kard-e
people-EZ happy that Shah country RÂ desertion do.PAS-EZ
irân jašn gereft-and
Iran feast take.PAS-3.PL
(putatively) 'The people of Iran happy that the Shah left the country celebrated.'

The same adjectives, however, may take a nominal, prepositional or sentential complement when they occur outside the Ezafe domain, in apposition for instance, as illustrated by the following examples:

(14) (a) mard-i, negarân-e bačče-hâ-yaš, vâred šod
homme-INDEF worried-EZ child-PL-PAF.3.SG entered become.PAS
'A man, worried about his children, entered.'
(b) mardom-e Tehran, xašmgin az erteja, be-pâ xâst-and
people-EZ Tehran angry at reactionaries to-foot stand.PAS-3.PL
'The people of Tehran, angry at the reactionary forces, rose up.'
(c) mardom-e iran, xošhâl ke šâh kešvar râ tark kard,
people-EZ Iran happy that Shah country RÂ desertion do.PAS
jašn gereft-and
feast take.PAS-3.PL
'The people of Iran, happy that the Shah left the country, celebrated.'

As for prepositions, Samiian claims that they may appear with a nominal complement within the Ezafe domain, but that sentential complements are excluded:

(15) (a) âftâb-e ba'd az bârun qašang-e
sun-EZ after from rain beautiful-COP.3.SG
'The sun after the rain is beautiful.'
(b) *âftâb-e ba'd az in-ke bârun bi-âd qašang-e
sun-EZ after from this-that rain MA-come.PRES beautiful-COP.3.SG
(putatively) 'The sun after it has rained is beautiful.'
(c) âftâb, ba'd az in-ke bârun bi-âd, qašang-e
sun, after from this-that rain MA-come.PRES beautiful-COP.3.SG
'The sun, after it has rained, is beautiful.'

Despite these facts, Samiian nevertheless considers that the constituents occurring within the Ezafe domain are all maximal projections (NPs, PPs and APs).

This content downloaded from 143.167.2.135 on Thu, 19 Nov 2015 22:53:22 UTC
All use subject to JSTOR Terms and Conditions

3.2 *The Ezafe domain as a domain of X°s: Ghomeshi (1997b)*

Relying to a large extent on Samiian's data, Ghomeshi (1997b) takes an important theoretical departure in her account of the Ezafe construction. The following assumptions on syntactic configurations underlie her analysis: a) Heads may adjoin to one another without projecting; b) Some X° categories are inherently non-projecting in syntax; c) Whether a constituent is phrasal or not depends not only on whether the head projects, but on whether that head itself is selected by a projecting element. While certain X° categories may be inherently non-projecting in syntax, they may still appear as XPs, provided they are selected by a projecting head.

On the basis of these assumptions, Ghomeshi puts forward three hypotheses:

(i) Persian nouns are inherently non-projecting. Consequently they never appear with filled specifier and complement positions and the NP node cannot dominate any phrasal material.
(ii) In spite of the fact that they are non-projecting, Persian nouns may still appear as NPs, provided they are selected by a projecting head (e.g. D°).
(iii) The Ezafe never attaches to a phrase, which implies that the Ezafe domain is the domain of X°s or bare heads.

The structure in (16) follows from (i)–(iii) above:

(16) The Persian NP (Ghomeshi 1997b)

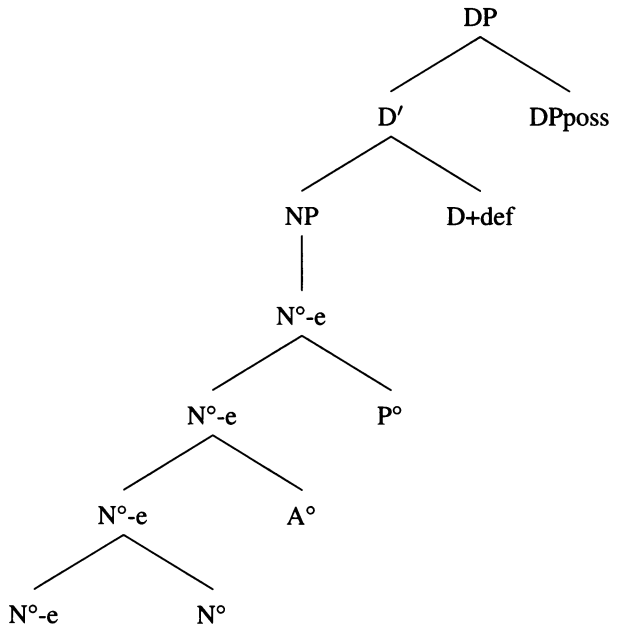

This content downloaded from 143.167.2.135 on Thu, 19 Nov 2015 22:53:22 UTC
All use subject to JSTOR Terms and Conditions

The Ezafe Insertion rule, operating in PF, inserts the Ezafe vowel *-e* on a lexical X° head that bears the feature [+N], when it is followed by phonetically realized, non-affixal material within the same extended projection.

An important point in (16) is that the possessor NP (or DP) is not dominated by the NP but is instead base-generated as sister to D′. This follows from Ghomeshi's assumption about the non-projecting nature of Persian nouns. Since the latter cannot dominate phrasal material, the NP-internal position is excluded for the possessor, which can be fully phrasal. It must consequently occur either as a sister to NP or as a sister to D′. The first option is not adopted by Ghomeshi, who advocates that the possessive DP be base-generated in [Spec, DP] position. Under this assumption, an empty D-head bearing the feature [+ def] is required, whose validity is further supported by the following facts: a) NPs including a possessor are obligatorily construed as definite or presupposed; b) Possessors are in complementary distribution with the indefinite determiner *-i*.

The definiteness of an NP containing a possessor receives a straightforward explanation if a definite article is present, the whole NP thus being a DP. The complementary distribution of possessors and the indefinite *-i* is attributable to the properties of the two D-heads in Persian: the empty definite determiner assigns Case, while *-i* does not. This prevents a possessor DP from occurring with the indefinite *-i* and thus accounts for the ungrammaticality of the following example:

(17) *ketâb-e sorx-i maryam
book-EZ red-INDEF Maryam
(putatively) 'a red book of Maryam'

In the light of this analysis, the restrictions pointed out by Samiian (1983) are straightforwardly accounted for. The only cases that would seem to resist Ghomeshi's analysis are represented by PPs. The latter can occur within the Ezafe domain and are therefore expected to appear there as bare heads. However, they seem to take complements, as illustrated by the following examples:

(18) (a) molâqât-e qabl az zohr-e mâ jâleb bud
meeting-EZ before from noon-EZ us interesting be.PAS
'Our meeting before noon was interesting.'
(b) bahs-e bâ ajale-ye hasan jâleb bud
discussion-EZ with haste-EZ Hasan interesting be.PAS
'Hasan's hasty discussion was interesting.' (Samiian 1983: 55)

This content downloaded from 143.167.2.135 on Thu, 19 Nov 2015 22:53:22 UTC
All use subject to JSTOR Terms and Conditions

However, Ghomeshi claims that the counter-evidence represented by these examples is only apparent. The noun within the modifying PP cannot appear with a possessor when the PP occurs within the Ezafe construction, as illustrated in (19b), while it can if the PP is appositive, as illustrated in (19c):

(19) (a) otâq-e kučik-e zir-e širvuni-e ali
room-EZ small-EZ under-EZ roof-EZ Ali
'Ali's small room under the roof' (Samiian 1983: 39)
(b) *otâq-e kučik-e zir-e širvuni-e jiân-e ali
room-EZ small-EZ under-EZ roof-EZ Jian-EZ Ali
(putatively) 'Ali's small room, under Jian's roof'
(c) otâq-e kučik-e ali, zir-e širvuni-e jiân
room-EZ small-EZ Ali under-EZ roof-EZ Jian
'Ali's small room, under Jian's roof'

These examples show that P2 and P3 prepositions behave like adjectives do, in that they cannot take a phrasal complement within the Ezafe domain. As for P1s, which are real prepositions, Ghomeshi suggests that they exhibit a dual nature: they can either take phrasal complements in syntax, or form compounds in the lexicon. In order to account for (18a) and (18b), which seem to be counterexamples, Ghomeshi considers that *bâ ajale* 'with haste' and *qabl az zohr* 'before noon' are not in fact PPs, but lexicalized compounds. Since compounding takes place in the lexicon, such sequences are inserted under a single terminal node and their internal structure is opaque for the purposes of syntax.

3.3 *Empirical problems with Ghomeshi's account*

In what follows, it will be shown that the expansion restrictions on the Ezafe construction mentioned by Samiian (1983) and taken up by Ghomeshi (1997b) do not hold and that Persian NPs can indeed include phrasal modifiers.

Recall that the ungrammaticality of the following NP ((11b), repeated here as (20)) supports the claim that Persian nouns may never take phrasal modifiers:

(20) *mard-e negarân-e bačče-hâ-yaš-i vâred šod
man-EZ worried-EZ children-PL-PAF.3.SG-INDEF entered become.PAS
(putatively) 'A man worried about his children entered.'

Although (20) is indeed ungrammatical, I will argue that this is in no way related to the presence of a phrasal modifier, namely the AP, within the

This content downloaded from 143.167.2.135 on Thu, 19 Nov 2015 22:53:22 UTC
All use subject to JSTOR Terms and Conditions

NP. The following attested example from a Persian novel, where *negarân* 'worried' heads an AP which is linked to the noun *cašm* 'eye' by the Ezafe, supports this claim:

(21) abdoljalil ham bâ rang-e paride va **čašm-ân-e**
Abdoljalil also with colour-EZ fly.PPL and eye-PL-EZ
[AP **negarân-e** forurixtan-e divâr-e xâne-aš] ântaraftar
worried-EZ crumble-EZ wall-EZ house-PAF.3.SG farther
istâde (...) bud.
stood be.PAS
'Abdoljalil also with his pale figure and his **eyes worried about the crumbling of the wall of his house** was standing farther.'
(*Ruzegâr-e separi šode-ye mardom-e sâlxorde*, M. Dowlatâbâdi, p. 113)

Here, the NP headed by *čašm* 'eye' contains a phrasal modifier, namely the AP *negarân-e forurixtan-e divâr-e xâne-aš* 'worried about the crumbling of the wall of his house'. Consequently the ungrammaticality of (20) cannot be attributed to the phrase-hood of the adjectival modifier. What makes example (20) ungrammatical is the presence of the indefinite enclitic *-i*. The following example, where *-i* has been removed, is perfectly grammatical:

(22) mard-e negarân-e bačče-hâ-yaš vâred šod
man-EZ worried-EZ children-PL-PAF.3.SG entered become.PAS
'The man worried about his children entered.'

The reason why *-i* cannot occur in this position will be discussed later. For the moment, the relevant fact to be considered is that Persian NPs clearly can contain phrasal modifiers. Examples such as (21) are by no means exceptional and are frequently encountered in contemporary Iranian literature and newspapers:

(23) šahr-e tehrân tavassot-e niru-hâ-ye qazzâg-e
city-EZ Tehran by means-EZ force-PL-EZ Kozak-EZ
[AP mostaqqar dar qazvin] fath šode bud
based in Qazvin conquered became be.PAS
'The city of Tehran was conquered by Kozak forces based in Qazvin.'
(*In se zan* (novel), F. Behnud, p. 40)

(24) Agar sabr kon-id zan-e [AP ziba-tar va
if waiting do.PRES-2.SG woman-EZ beautiful-CPR and
tannâz-tar az ân] râ peydâ xâhid kard
charming-CPR of this RÂ found AUX do.PAS
'If you wait you'll find a more beautiful and charming woman than her'
(Dašti, quoted by Lazard 2006: 173)

This content downloaded from 143.167.2.135 on Thu, 19 Nov 2015 22:53:22 UTC
All use subject to JSTOR Terms and Conditions

The same situation holds for prepositions: P1s, as well as P2s and P3s, can occur within the Ezafe domain even when they head phrasal projections:

(25) ruz-e [$_{PP}$ qabl az dastgiri-e farmânfarmâ va pesar-ân-aš]
day-EZ before of arrest-EZ Farmânfarma and son-PL-PAF.3.SG
'the day before Farmânfarmâ's and his sons' arrest'
(*In se zan* (novel), F. Behnud, p. 65)

According to Ghomeshi's analysis, *dastgiri-e farmânfarmâ va pesar-ân-aš* 'Farmânfarmâ's and his sons' arrest' must be considered as a DP with a filled [Spec, DP] position. This DP appears within a constituent headed by the P1 *qabl az* 'before', the whole sequence thus being a PP. It follows that, contrary to Ghomeshi's claim, P1s may appear within the Ezafe domain not only as compounds but also when they head a real PP. Here are some other examples:

(26) čerâ xâne-ye [$_{PP}$ be ân qašangi] râ foruxte
why house-EZ to that beauty RÂ sell.PPL
'Why has she sold such a beautiful house?'

(27) ne-mi-tavânest-am tasmim be-gir-am sobh-hâ-ye
NEG-MA-can.PAS-1.SG decision MA-take-1.SG morning-PL-EZ
[$_{PP}$ bâ mâdar] râ bištar dust dâr-am yâ sobh-hâ-ye
with mother RÂ more like.PRES-1.SG or morning-PL-EZ
[$_{PP}$ bâ kabutar-hâ] râ.
with pigeon-PL RÂ
'I could not decide whether I loved the mornings with mother or the ones with the pigeons more.'
(*Yek ruz mânde be eyd-e pâk* (novel), Z. Pirzâd, p. 80)

As for P2s and P3s, like P1s and adjectives, they too can head a phrasal constituent within the Ezafe domain, as shown by the following example:

(28) sekke-hâ-ye [$_{PP}$ tu-ye jib-e šalvâr-aš] oftâd-and
coin-PL-EZ in-EZ pocket-EZ trousers-PAF.3.SG fall.PAS-3.PL
'The coins in his trousers' pocket fell down.'

To sum up, the only modifiers which cannot be linked to the head noun by the Ezafe are relative clauses. At this point, the major restrictions on the Ezafe construction mentioned by Samiian (1983) and Ghomeshi (1997b) have turned out to be ill-founded. The fact that Persian NPs can dominate phrasal material seriously challenges Ghomeshi's hypothesis about Persian nouns being non-projecting.

Since the phrase-hood of the constituents can no longer be responsible for the ungrammaticality of examples such as (20) (=(11b)), the question remains as to what the real restrictions on the Ezafe construction are and how they may be accounted for. These questions will be discussed in section (4), where it will be argued that the affixal status of the Ezafe and its interplay with other affixes constitute the right option to explore. Before moving to

This content downloaded from 143.167.2.135 on Thu, 19 Nov 2015 22:53:22 UTC
All use subject to JSTOR Terms and Conditions

this issue, I will briefly examine an alternative analysis, initially proposed by Samiian (1994) and more recently by Larson & Yamakido (2005), which consists in viewing the Ezafe as a case-marker.

3.4 *Is the Ezafe particle a case-marker?*

Samiian (1994) argues in favor of considering the Ezafe as a dummy case-assigner occurring with non-case-assigning heads (nouns, adjectives, P2s and P3s) and enabling them to case-mark their complements. Since case-marking is typically associated with argument status and not with modifiers, the question arises of why modifiers would require Case in Persian. For Samiian, this is not a problem, given the existence of languages such as Sanskrit and Latin that case-mark attributive APs.

The analysis of the Ezafe as a case-marker, however, faces serious problems, the main one being that the data it is based on is not well grounded.[4] Indeed, the major argument invoked by Samiian (1994) to support this analysis is the fact that constituents such as PPs and relative clauses, which do not need to be case-marked, are excluded from the Ezafe domain. As was shown above, however, PPs headed by P1s as well as adverbial phrases do occur within the Ezafe domain. Furthermore, although relative clauses cannot be linked to the head noun by the Ezafe, reduced relatives, by contrast, are introduced by the Ezafe:

(29) nazm-e ostân tavassot-e in javân-e
order-EZ province by means-EZ this young-EZ
$[_{RRC}$ az suis bar gašte] bištar hâsel xâhad âmad
from Switzerland back turn.PPL more gained AUX come.PAS
'Order in the province will be better established by this young man (who has) come back from Switzerland.'
(*In se zan* (novel), F. Behnud, p. 55)

(30) aks-e $[_{RRC}$ čâp šode dar ruznâme]
photo-EZ publication become.PP in newspaper
aks-e râvi-e dâstân ast
photo-EZ narrator-EZ story be.PRES
'The photo published in the newspaper is the photo of the narrator of the story.'
(*Dâstanha-ye mahbub-e man*, A. Darvišian R. X. Mahâbâdi, p. 274.)

It is unclear why constituents such as PPs and reduced relatives should need to be case-marked in Persian.[5]

[4] Larson & Yamakido (2005) also suggest viewing the Ezafe as a case-marker which checks the case assigned by the determiner head of a DP to its arguments, allowing the latter to remain in situ. Their analysis faces the same problems as that of Samiian (1994).

[5] Viewing the Ezafe as a case-marker becomes even more problematic when other West Iranian languages are taken into account. Indeed, in almost all the groups of Kurdish

This content downloaded from 143.167.2.135 on Thu, 19 Nov 2015 22:53:22 UTC
All use subject to JSTOR Terms and Conditions

4. The Ezafe as a (phrasal) affix

In this section I shall sketch an affixal analysis of the Ezafe. On the basis of distributional, prosodic and morphological criteria, I shall establish two major sets of inflectional affixes within the Persian NP.

The members of the first set, i.e. the definite suffix -(*h*)*e*[6] and the plural suffix -*hâ*, may be considered as word-level inflectional affixes: they attach to the head (i.e. the noun) within the NP and cannot be separated from it by any other inflectional affix. Furthermore, they bear lexical stress and cannot have wide scope over the coordination of two nouns.

The members of the second set, i.e. the Ezafe, the determiner -*i* and personal enclitics, will be argued to be phrasal (inflectional) affixes: they occur at the right edge of nominal non-maximal projections, and are positioned after Set (1) inflectional affixes. It will be further argued that their status, or more precisely their membership in the same set, can account for the ungrammaticality of certain examples provided by Samiian (1983), such as (20) (=(11b)) discussed in Section (3.3) and repeated below:

(31) *mard-e negarân-e bačče-hâ-yaš-i vâred šod
man-EZ worried-EZ child-PL-PAF.3.SG-INDEF entered become.PAS
(putatively) 'A man worried about his children entered.'

The ungrammaticality of this example will be accounted for in terms of constraints on the cooccurrence, or rather the impossibility of cooccurrence, of two affixes, namely the determiner -*i* and the personal affix -*yaš*, on the same host.[7]

4.1 *Set (1) inflectional affixes (word-level affixes)*

The plural suffix *hâ*[8] and the colloquial definite suffix -(*h*)*e* belong to this class. These two affixes: a) are in complementary distribution; b) occur on

dialects and in Zazaki, restrictive relative clauses can be introduced by the Ezafe (see Mackenzie 1961, Bedir-Khan & Lescot 1970, Bassols-Codina 1992, Paul 1998 and Fattah 2000, among others), even though it is generally assumed that CPs are never case-marked.

[6] As mentioned earlier, there is no overt definite article in standard Persian; but colloquial Persian does have a definite suffix, realized as -*he* after a vowel and -*e* in other contexts.

[7] Though I will not use the technical device of Paradigm Function morphology (Stump 2001) to state these constraints, they can be formulated within this framework in terms of position class morphology, where collections of items compete for realization in a single position.

[8] *Hâ* is the most frequently used plural suffix in Persian, since it can regularly adjoin to nouns, regardless of their semantic properties or their etymology. Persian has two other plural suffixes, -(*y*)*ân* and -*ât*. The first is used with animates. The second, which is an Arabic suffix, occurs with substantives inherited from Arabic. The analysis of -*hâ* which is defended in this section can be applied to these two suffixes, since they display the same placement and prosodic properties.

This content downloaded from 143.167.2.135 on Thu, 19 Nov 2015 22:53:22 UTC
All use subject to JSTOR Terms and Conditions

the head noun within the NP; c) cannot be separated from their host by another inflectional affix; d) bear lexical stress; e) cannot have wide scope over coordination.

The following example illustrates point (a):

(32) *ketâb-e-hâ/-hâ-e
book-DEF-PL/PL-DEF
(putatively) 'the books'

With respect to their placement properties, *-hâ* and *-(h)e* behave rather like head inflections, since in principle they always attach to the head noun within the NP. This means that they normally cannot occur on modifiers which follow the noun:

(33) (a) in pesar-e-ye ahmaq
this boy-DEF-EZ silly
'this silly boy'
(b) *in pesar-e ahmaq-e
this boy-EZ silly-DEF
(putatively) 'this silly boy'

(34) (a) in pesar-hâ-ye ahmaq
this boy-PL-EZ silly
'these silly boys'
(b) *in pesar-e ahmaq-hâ
this boy-EZ silly-PL
(putatively) 'these silly boys'

Note that the following examples, where the definite and the plural suffixes are not attached to the head noun, are nevertheless grammatical:

(35) *lebâs qermez bi âstin-e/-hâ tu komod-e/an
dress red without sleeve-DEF/-PL in wardrobe-COP.3.SG/COP.3.PL
'The red dresse(s) without sleeves is/are in the wardrobe.'

In this case, however, it should also be noted that the Ezafe is excluded between the noun and its modifiers, which are simply juxtaposed. Furthermore *-(h)e* and *-hâ* cannot be attached to the head noun in this case:

(36) *lebâs-e/-hâ qermez bi âstin tu komod-e/-an
dress-DEF/-PL red without sleeve in wardrobe-COP.3.SG/PL
(putatively) 'The red dress(es) without sleeves is/are in the wardrobe.'

The ungrammaticality of (36) seems to indicate that sequences formed by a noun and juxtaposed modifiers exhibit some degree of opacity and that their internal structure is inaccessible to syntax. This assumption is further

This content downloaded from 143.167.2.135 on Thu, 19 Nov 2015 22:53:22 UTC
All use subject to JSTOR Terms and Conditions

supported by the impossibility of coordination of two modifiers that are juxtaposed to the head without the Ezafe:

(37) (a) *yek lebâs zibâ va boland
one dress beautiful and long
(putatively) 'a long and beautiful dress'
(b) yek lebâs-e zibâ va boland
one dress-EZ beautiful and long
'a long and beautiful dress'

The sequences in question are thus comparable to compound nouns, which are also formed by the juxtaposition of lexical elements in Persian. Although I cannot propose an appropriate analysis for these sequences, I will assume that the facts just mentioned provide evidence against viewing them as ordinary nominal projections. Consequently, the fact that the definite or the plural suffix occurs on the last modifier and not on the head noun is not an argument against a word-level affix analysis of the suffixes, but rather a consequence of the non-ordinary syntactic properties of the construction.

Both the definite and the plural suffix bear lexical stress. At this point, it should be mentioned that lexical stress falls on the last syllable in Persian nouns and adjectives. When the plural or the definite suffix is adjoined to a noun, the lexical stress moves one syllable to the right, and falls on the suffix. In this respect, these suffixes behave like derivational suffixes.

Finally, neither the plural *-hâ* nor the definite *-(h)e* may have wide scope over the coordination; they must be repeated on each conjunct, as illustrated by the contrast between (38a) and (38b):

(38) (a) ketâb-e-o daftar-e ru-ye miz-an
book-DEF-and notebook-DEF on-EZ table-COP.3.PL
'The book and the notebook are on the table.'
(b) *ketâb-o daftar-e ru-ye miz-an
book-DEF-and notebook-DEF on-EZ table-COP.3.PL
(putatively) 'The book and the notebook are on the table.'

Let us now examine the behavior of these suffixes with respect to the Ezafe. Crucial to our discussion here is the fact that, contrary to the enclitic determiner *-i*, the definite *-(h)e* may combine with the Ezafe, as shown in (33a). Here is another attested example:

(39) in bâr âhang hamân-i bud ke **pesar-e-ye** film-e
this time melody same-RESTR be.PAS that boy-DEF-EZ film-EZ
hendi barâ-ye doxtar-e mi-zad
Indian for-EZ girl-DEF MA-beat.PAS
'This time the melody was the same as the one the boy in the Indian film was playing for the girl.'
(*Sâz dahani* (short story), Z. Pirzâd)

This content downloaded from 143.167.2.135 on Thu, 19 Nov 2015 22:53:22 UTC
All use subject to JSTOR Terms and Conditions

I will assume that the contrast between -(*h*)*e* and -*i* with regard to the Ezafe is due to their different affixal status: while -(*h*)*e* is a word-level affix, -*i* is a phrasal affix. In other words, these two elements belong to two different classes of affixes. This point will be argued thoroughly below. For the moment we shall limit ourselves to the following observation: -(*h*)*e* can occur with the Ezafe, and in this case the Ezafe follows the definite suffix.

4.2 *Set (2) inflectional affixes (phrasal affixes)*

In the remainder of this section, I will argue that the affixal analysis is also appropriate for the Ezafe, the determiner -*i* and the personal enclitics, which constitute a second set of inflectional affixes within the Persian NP. These items: a) are in complementary distribution; b) do not necessarily occur on the head noun within the NP and can attach to the right edge of some intermediate projections; c) are compatible with the Set (1) inflectional affixes -*hâ* and -(*h*)*e* and are placed after them; d) do not bear lexical stress; e) can have wide scope over coordination.

Before moving on to the discussion of the affixal properties of these items, a brief presentation of the determiner -*i* and of the set of personal enclitics will be useful at this point.

4.2.1 *Personal enclitics and the determiner -i: a brief survey*

Within the NP, the personal enclitics (-*am* 1.SG, -*at* 2.SG, -*aš* 3.SG, -*emân* 1.PL, -*etân* 2.PL, -*ešân* 3.PL) alternate with full pronouns and the possessor NP, and receive the same range of interpretations:

(40) ketâb-am
book-PAF.1.SG
'my book'

Note that in contrast to full pronouns, personal enclitics are not introduced by the Ezafe and are attached directly to their host. In case the head noun is followed by modifiers introduced by the Ezafe, as in (41), the personal enclitic is attached to the last modifier:

(41)	lebâs-e	sefid-e	bi	âstin-am
	dress-EZ	white-EZ	without	sleeve-PAF.1.SG
	'my white dress without sleeves'			

It should be mentioned that the same enclitics can also occur within adjectival and some prepositional phrases. In this case, they are attached to the head and realize the single complement of the latter. They can also be used in relation to a verbal head in order to realize its direct object.

As for the enclitic determiner -*i*, it is one of the most problematic elements within the Persian NP. My purpose here is by no means to provide an

This content downloaded from 143.167.2.135 on Thu, 19 Nov 2015 22:53:22 UTC
All use subject to JSTOR Terms and Conditions

exhaustive account of its morphological, syntactic and semantic properties, but only to highlight some of its distributional aspects which are relevant to the analysis of the Ezafe particle outlined in this section. Persian grammars generally establish two distinct determiners *-i* in Persian. The first is considered to be an indefinite article. The second, which occurs exclusively with restrictive relatives, is analyzed as a 'demonstrative' or 'definite' article. The term 'restrictive particle' has also been used in some studies. The following examples illustrate each case:

(42) (a) maryam dar asemân setâre-i did
Maryam in sky star-INDEF see.PAS
'Maryam saw a star in the sky.'
(b) setâre-i ke maryam dar asemân did
star-RESTR that Maryam in sky see.PAS
'the star that Maryam saw in the sky'

There has been a long-standing debate in the literature on the enclitic *-i* as to whether the two determiners are in fact one and the same morpheme, that is, neither an indefinite nor a definite article, but a 'selective' article. This issue, thoroughly dealt with in several studies, is beyond the scope of our discussion here and I shall not take a stand on it (cf. Hincha 1961, Lazard 1966 and Samvelian 2006, among others). Anyway, as the two enclitics attach to the same range of hosts, whatever alternative is adopted, it is of no consequence for the analysis suggested in this paper, which can successfully accommodate both options.

Like the personal enclitics, in the presence of modifiers linked to the head noun by the Ezafe, the determiner *-i* does not attach to the head noun, but to the last modifier within the Ezafe domain:

(43) (a) lebâs-e qermez-e bi âstin-i
dress-EZ red-EZ without sleeve-INDEF
'a red dress without sleeves'
(b) irâni-e javân-e moqim-e farânse-i ke did-im
Iranian-EZ young-EZ resident-EZ France-RESTR that see.PAS-1.PL
'The young Iranian resident of France whom we met'

Both the enclitic *-i* and the personal affixes are compatible with word-level inflectional affixes (i.e. *-hâ* and *-(h)e*) and are placed after them, a property they share with the Ezafe. Furthermore, like the Ezafe, and contrary to the definite *-(h)e* and the plural *-hâ*, they do not bear lexical stress.

Given these facts, if the three types of enclitics under investigation are suffixes, they clearly form a distinct class from *-(h)e* and *-hâ*. Since they can either be attached to a head or to a constituent (i.e. N′), they would be best regarded as phrasal affixes. However, the range of properties they exhibit makes them *a priori* appropriate candidates for being simple postlexical

This content downloaded from 143.167.2.135 on Thu, 19 Nov 2015 22:53:22 UTC
All use subject to JSTOR Terms and Conditions

clitics (or bound words) as well. In what follows, I will first present a brief review of different criteria proposed in the literature to separate (phrasal) affixes from postlexical clitics. Relying on these criteria, I will then show that the behavior of these enclitics provides rather conclusive evidence in favor of the affixal view.

4.2.2 *Phrasal affixes vs. postlexical clitics*

Since Zwicky (1977), several studies on clitics have converged on the conclusion that the pre-theoretical notion of 'clitic' must be replaced by (at least) two quite distinct notions: postlexical clitics or bound words (i.e. words lacking prosodic structure and attached postlexically to their phonological host) on the one hand, and affixes on the other hand.[9] Whereas normal inflectional affixes are word-level affixes realized on the head, thus attaching to words of particular categories, 'clitic affixes' generally occur on the edge of a constituent and thus attach to whatever word happens to occur in phrase-initial or phrase-final position. The term 'phrasal affix' is used to designate these affixal elements occurring on the edge.

Given the similarities between postlexical clitics and phrasal affixes, it is not always easy to determine whether an item in a given language belongs to the former or the latter category. Zwicky & Pullum (1983) suggest a set of six diagnostic criteria, relying on distributional, morphophonological, semantic and syntactic properties, to separate affixes from clitics. The overall thrust of these criteria is that affixes are characterized by a high degree of selectivity with respect to their host (Criterion A) and idiosyncrasy in their realization and behavior (Criteria B, C and D),[10] while clitics display a high degree of promiscuity in their attachment and regularity in their realization and behavior.

As has been noted by Miller (1992), however, these criteria do not always prove to be of great help when it comes to distinguishing *phrasal* affixes (or edge inflections) from postlexical clitics. Indeed, in many respects, phrasal affixes resemble postlexical clitics rather than inflectional affixes. They exhibit a low degree of selection with respect to their host and thus are promiscuous in their attachment. They do not necessarily exhibit arbitrary gaps and morphological or semantic idiosyncrasies.[11]

[9] In reality, the picture is much more complex than the one presented here. For Klavans (1985), for instance, clitics constitute a unitary phenomenon and could be considered affixes with phrasal determination, i.e. phrasal affixes. In this paper, I will adopt a binary approach to clisis, in line with Zwicky & Pullum (1983), Zwicky (1985, 1987), Nevis (1988), Miller (1992): 'clitics' will be considered either postlexical clitics (i.e. bound words) or affixes.

[10] Zwicky & Pullum's Criteria E and F are not relevant to the discussion here.

[11] As noted by Miller (1992), Criterion D (i.e. semantic idiosyncrasies) is implausible in any case. For the details of the argument see Miller (1992: 141).

This content downloaded from 143.167.2.135 on Thu, 19 Nov 2015 22:53:22 UTC
All use subject to JSTOR Terms and Conditions

Pointing out these problems with Zwicky & Pullum (1983), Miller (1992) concludes that morphological criteria introduce a certain bias in favor of affixal status, but 'have the disadvantage of never making it possible to decide in favor of clitic status' (p. 141). Completely regular processes, with possible subregularity and irregularity, can be integrated by morphological systems. Therefore a lack of attested idiosyncrasy is not incompatible with affixal status.[12] Miller (1992) therefore argues in favor of a refinement of Zwicky & Pullum's (1983) Criterion C in order to take into account whether a parallelism appears with respect to morphophonological rules applying in the context of other well-established affixes within a given language.

Another refinement contributed by Miller (1992), relying on Zwicky (1987), is the Haplology Criterion. The label 'haplology', familiar to phonology, is used by Miller (1992) to refer to the impossibility of two identical, partially identical or even dissimilar affixes occurring in contiguous positions. The well-known example pointed out by Zwicky (1987) is the impossibility of English POSS /z/ being realized on an item which ends in the plural affix *-s*, another /z/ affix. In other words, whatever the number of /z/ affixes required on a host may be, only a single /z/ can be realized. Since syntactic items, and consequently postlexical clitics, are necessarily concatenative, items involving haplology phenomena cannot be postlexical clitics and must be affixes.

Miller (1992) further suggests a Coordination criterion to separate clitics from affixes. The impossibility of having wide scope over a coordination of hosts, together with the necessity of being repeated on each conjunct, clearly entails affixal status. However, the converse is not true: it cannot be argued that an item is a postlexical clitic from the fact that it may have wide scope over coordination. Finally, a postlexical item can be repeated on each conjunct if and only if it forms a constituent with its host in syntax in the corresponding non-coordinated sentence.

In what follows, I will investigate the behavior of the three types of enclitics under discussion with respect to the aforementioned diagnostic tests. Though in some cases the results will turn out to be compatible with either an affixal or a postlexical reading, the Haplology Criterion will be shown to provide fairly conclusive evidence in favor of the affixal analysis of these enclitics.

[12] Miller (1992) further notes that Criterion C (i.e. morphophonological idiosyncrasies) is difficult to use as such if one adopts the hypothesis, often defended within the framework of lexical morphology, that lexical rules can apply one last time after postlexical cliticization. In the light of this hypothesis, Criterion C must be reinterpreted: only items that undergo non-last-level phonological rules must be affixes and cannot be postlexical clitics (p. 141).

This content downloaded from 143.167.2.135 on Thu, 19 Nov 2015 22:53:22 UTC
All use subject to JSTOR Terms and Conditions

4.2.3 *Promiscuous attachment, morphophonological idiosyncrasies and arbitrary gaps* (*Zwicky & Pullum 1983*)

As noted earlier, all of these enclitics are compatible with word-level inflections and are placed after them. Unlike the latter, they do not bear lexical stress. But since other elements whose affixhood is undisputed do not bear lexical stress either (e.g. verbal personal endings, which are always attached to the verbal stem and realize subject-verb agreement), this property is irrelevant for distinguishing affixes from postlexical clitics.

- Selectivity with respect to the host (Criterion A)

Although they combine with a wide range of hosts, the three types of enclitics under discussion are not totally promiscuous and display some degree of selection with respect to their hosts.

Personal enclitics enjoy great freedom in their attachment, since they can be adjoined to all major lexical categories (i.e. nouns, verbs, prepositions and adjectives). However, some restrictions are observed in the case of attachment to a preposition. Despite the fact that all prepositions can take an NP complement, the adjunction of personal enclitics to P1 prepositions (i.e. those that cannot take the Ezafe) is excluded in the formal register (Lazard 1992):

(44) (a) tu-ye otâq / tu-yaš
inside-EZ room inside-PAF.3.SG
'in the room' / 'inside it'
(b) dar otâq / *dar-aš
in room in-PAF.3.SG
'in the room / (putatively) 'inside it'

Recall that the same holds for the Ezafe with respect to prepositions: some necessarily occur with the Ezafe (P2s), some optionally take the Ezafe (P3s), while still others simply exclude it (P1s). Furthermore, the Ezafe cannot attach to finite verbs, but only to infinitives and participles.

As for the determiner *-i*, nowhere was attachment to a preposition observed. However, it is not clear whether this is a true restriction or the consequence of the fact that prepositions never appear in the rightmost position of intermediate constituents within the noun phrase. Like the Ezafe, *-i* cannot attach to finite verbs.

To conclude, despite the fact that they enjoy great freedom, none of the three types of enclitics under study is totally promiscuous in its attachment. The Ezafe and the determiner *-i* are located at mid-scale, while personal enclitics, which display a higher degree of promiscuity, are nevertheless excluded with P1s.

- Arbitrary gaps (Criterion B)

This content downloaded from 143.167.2.135 on Thu, 19 Nov 2015 22:53:22 UTC
All use subject to JSTOR Terms and Conditions

The adjunction of personal enclitics to some categories is subject to arbitrary gaps. Among P2 and P3 prepositions, which are expected to be appropriate hosts for personal enclitics, some cannot occur with a personal enclitic:

(45) bedun-e maryam / *bedun-aš
without-EZ Maryam without-PAF.3.SG
'without Maryam' / (putatively) 'without her'

Another example is provided by P1 prepositions. Though in the formal register these cannot combine with personal enclitics, in colloquial Persian nevertheless three of them (*az* 'from', *be* 'to' and *bâ* 'with') do occur with personal enclitics:

(46) az maryam / az-aš
from Maryam from-PAF.3.SG
'from Maryam' / 'from her/him'

By contrast, the adjunction of the Ezafe and the determiner *-i* does not seem to involve arbitrary gaps.

- Morphophonological idiosyncrasies (Criterion C)

The adjunction of personal enclitics gives rise to various morphophonological idiosyncrasies. Compare the following examples:

(47) (a) pâ + -aš →pâ-yaš
foot PAF.3.SG
'her/his foot'
(b) bâ + -aš →bâ-hâš/*bâ-yaš
with PAF.3.SG
'with her/him'

(48) (a) gorbe + -aš →gorbe-aš
cat PAF.3.SG
'her/his cat'
(b) be + -aš →be-heš/*be-aš
to PAF.3.SG
'to her/him'

Both (47a) and (47b) illustrate the adjunction of the personal enclitic *-aš* to a word ending with the vowel /â/. In (47a), which illustrates the regular type of adjunction, a glide is inserted between the host and *-aš*. In (47b), by contrast, the adjunction of *-aš* in a similar phonological context involves the insertion of the consonant /h/ instead of the glide. Furthermore, the initial vowel of the enclitic mutates from /a/ to /â/, thus harmonizing with the vowel of the host.

Example (48) illustrates a similar phenomenon: the same enclitic *-aš* is adjoined to a word ending in /e/. In (48a), the regular case of adjunction, *-aš*

This content downloaded from 143.167.2.135 on Thu, 19 Nov 2015 22:53:22 UTC
All use subject to JSTOR Terms and Conditions

is simply appended to the host. In (48b), by contrast, the consonant /h/ is inserted instead and the initial vowel of the enclitic mutates from /a/ to /e/, once again in harmony with the vowel of the host. At this point, it should be mentioned that Persian does not in general display vowel harmony rules. Though there are some extremely limited cases of phonological alternations within words which are reminiscent of vowel harmony (Lazard 1992). However, since they never occur across words, (47b) and (48b) provide further evidence in favor of the affixal nature of the personal enclitics.

Unlike the personal enclitics, however, the Ezafe and the determiner *-i* display a rather regular morphology. The Ezafe has two forms, whose distribution is phonologically conditioned: *-e* after all consonants and /i/; *-ye* in all other contexts. The enclitic determiner is regularly realized as *-i*, except after /â/, /u/ and /i/, where it is realized as *-yi*.

To conclude, with respect to their morphological and attachment properties, personal enclitics behave rather clearly as affixes. Their affixal status straightforwardly accounts for the fact that the Ezafe cannot be inserted between them and their host. The situation is less clear when it comes to the Ezafe and the determiner *-i*, whose morphophonological properties could be accommodated by either an affixal or a postlexical clitic account. Consequently, further arguments are required to support the affixal analysis of these two types of enclitics.

4.2.4 *Haplology criterion: Zwicky (1987), Miller (1992)*

The major argument in favor of the affixal view of the enclitics under discussion is provided by restrictions on their cooccurrence. It will be shown that:

(a) they are in complementary distribution (i.e. in competition) when adjoined to the right edge of the same constituent;
(b) any sequence containing two or more of these enclitics is excluded, even when their scope is not the same constituent.

In order to illustrate (a), let us take the following examples, involving an indefinite NP composed of a noun and an adjective:

(49) (a) xâne-ye digar-i
house-EZ another-IND
'another house'
(b) xâne-i digar
house-INDEF another
'another house'
(c) *xâne-i-e/-e-i digar
house-INDEF-EZ/EZ-INDEF another
(putatively) 'another house'

This content downloaded from 143.167.2.135 on Thu, 19 Nov 2015 22:53:22 UTC
All use subject to JSTOR Terms and Conditions

(d) *xâne digar-i
house another-INDEF
(putatively) 'another house'

There is nothing new to be said about (49a): the adjectival modifier follows the head noun to which it is linked by the Ezafe, and the determiner *-i* is attached to the adjective. Example (49b), by contrast, illustrates a construction that has not been encountered in this paper so far. Instead of being adjoined to the modifier, *-i* is adjoined to the head noun. Relevant to our discussion here is the fact that the Ezafe is excluded in this case, as shown by (49c). Example (49d) shows that in the absence of *-i*, the Ezafe is required in order for the noun and the modifier to form a constituent.[13]

These facts show that the enclitic *-i* and the Ezafe cannot co-occur on the same host. Informally speaking, if they are in competition for being adjoined to the same host, the determiner *-i* 'wins' over the Ezafe and further assumes the latter's role in some way. This intuition has sometimes been stated in previous studies. Perry (2005), for instance, uses the term 'Split Ezafe' (p. 74) for the enclitic *-i* in examples such as (49b). Lazard (1966) expresses a similar judgement not only about the indefinite *-i*, but also for those cases where *-i* introduces a restrictive relative clause. He notes that in addition to its role as a determiner, *-i* acts in such contexts as a linker, being thus comparable to the Ezafe (p. 257).

The facts under discussion are reminiscent of certain haplology phenomena pointed out by Miller (1992) involving weak functional words in French. Apart from examples of haplology between identical elements (e.g. * *le*+*le*), Miller provides examples where haplology phenomena occur between phonologically dissimilar sequences, as in (50) below:

(50) (a) mon livre le plus grand
my book the most big
'my biggest book'
(b) mon plus grand livre
my most big book
'my biggest book' (Miller 1992: 145, ex. (43))

[13] Contrary to Ghomeshi's (1997b) claim, the adjective in (49b) is not in apposition with respect to the noun (i.e. outside the NP), but lies within the NP. Indeed, the stress pattern of (49b) is identical to that of (49a) (i.e. the main stress is on the adjective). Furthermore, the semantic relation between theadjective and the noun is the same in both cases. Finally, even those adjectives that cannot stand in apposition with respect to the noun because of their semantic properties, such as *digar* 'another' in (49b), nevertheless can occur in this construction.

This content downloaded from 143.167.2.135 on Thu, 19 Nov 2015 22:53:22 UTC
All use subject to JSTOR Terms and Conditions

Here haplology involves the determiner *le* and the possessive *mon*, which cannot be cumulated:

(51) *mon le plus grand livre
my the most big book
(putatively) 'my biggest book'

Miller claims that these facts receive a natural explanation if the possessive and the definite determiner are the inflectional realizations of a set of morphosyntactic features. The fact that the possessive 'wins' over the definite article can thus be accounted for by assuming that the features realized by the possessive are subsumed by those realized by the definite article.

Let us return to (49) above, which illustrates a haplology phenomenon similar to that seen in Miller's example. Whether or not one adopts the idea suggested by Lazard (1966) and Perry (2005) to consider the enclitic *-i* as cumulating the function of both the indefinite determiner and the Ezafe, the impossibility of *-i* and the Ezafe cooccurring provides evidence in favor of an affixal analyses of the Ezafe and *-i*.

Another set of data illustrates point (b) above, that any combination of the three enclitics under discussion is excluded even when they have different scopes. Let us first consider the following examples:

(52) (a) qahremân-e [$_{RRC}$ az mihan-aš rânde šode]
hero-EZ from homeland-PAF.3.SG drive.PPL become.PPL
'the hero driven away from his homeland'
(b) qahremân-e [$_{RRC}$ rânde šode az mihan-aš]
hero-EZ drive.PPL become.PPL from homeland-PAF.3.SG
'the hero driven away from his homeland'

In both (52b) and (52a) a reduced relative clause, introduced by the Ezafe, is embedded within the NP headed by *qahremân* 'hero'. The two NPs differ solely with respect to the constituent order within the reduced relative clause. In (52b) the PP *az mihan-aš* 'from his homeland' precedes the participial head of the modifier, while in (52a) it follows the head. Though both (52b) and (52a) are grammatical, the addition of a possessor NP after the reduced relative is possible in the first case but not in the second:

(53) (a) qahremân-e [$_{RRC}$ az mihan-aš rânde
hero-EZ from homeland-PAF.3.SG drive.PPL
šode]-ye in roman
become.PPL]-EZ this novel
'the hero of this novel, (who is) driven away from his homeland'

This content downloaded from 143.167.2.135 on Thu, 19 Nov 2015 22:53:22 UTC
All use subject to JSTOR Terms and Conditions

(b) *qahremân-e [RRC rânde šode az
hero-EZ drive.PPL become.PPL from
mihan-aš]-e in dâstân
homeland-PAF.3.SG this novel
(putatively) 'the hero of this novel, (who is) driven away from his homeland'

This contrast can arguably be attributed to the fact that in (53b) the Ezafe is attached to the personal enclitic *-aš*, but not in (53a). Contrary to (49c), the Ezafe and the personal enclitic have two different scopes in (53b): the personal enclitic is attached to the NP *mihan* 'homeland', while the scope of the Ezafe is the whole N′ *qahremân-e rânde šode az mihan-aš*. These facts suggest that any sequence formed by a personal enclitic and the Ezafe is excluded in Persian.

The same kind of constraint is responsible for the ungrammaticality of (11b) (=(20)), given by Samiian (1983), and repeated in (54). Recall that removing the indefinite *-i* makes (54) perfectly acceptable (cf. example (22)).

(54) *mard-e negarân-e bačče-hâ-yaš-i vâred šod
man-EZ worried-EZ child-PL-PAF.3.SG-INDEF entered become.PAS
(putatively) 'A man worried about his children entered.'

It can thus be concluded that any combination of the three types of enclitics under discussion is excluded, regardless of their scope. This behavior is hard to reconcile with a post-clitic treatment, since syntactic items are generally assumed to be concatenative. The facts discussed so far, especially those concerning haplology, provide rather conclusive evidence in favor of an affixal analysis of the Ezafe, together with the enclitic *-i* and the set of personal enclitics.

4.2.5 *Coordination criterion*

The three types of enclitics under investigation can have wide scope over coordination of conjuncts and therefore they need not be repeated on each conjunct:

(55) (a) [kolâh-e sefid(-aš) va lebâs-e zard]-aš
[hat-EZ white(-PAF.3.SG) and dress-EZ yellow]-PAF.3.SG
'her/his white hat and yellow dress'
(b) [kolâh-e sefid(-i) va lebâs-e zard]-i
[hat-EZ white(-INDEF) and dress-EZ yellow]-INDEF
'a white hat and a yellow dress'
(c) [kolâh-e sefid(*-e) va lebâs-e zard]-e maryam
[hat-EZ white(-EZ) and dress-EZ yellow]-EZ Maryam
'Maryam's white hat and yellow dress'

This content downloaded from 143.167.2.135 on Thu, 19 Nov 2015 22:53:22 UTC
All use subject to JSTOR Terms and Conditions

As shown in (55a) and (55b), personal enclitics and the determiner *-i* can be repeated on each conjunct. Miller's Coordination Criterion is thus irrelevant to deciding between affixal and postlexical status, these facts being compatible with both options. By contrast, the Ezafe cannot be repeated on each conjunct, as shown in (55c), and must appear only once, on the second conjunct. It is not clear to me what the appropriate interpretation of this fact could be. None of Miller's criteria stipulates that the possibility of being repeated on each conjunct is a necessary condition for affixhood.

On the other hand, according to Miller's criterion (2), 'a postlexical clitic can be repeated on its host if and only if it forms a constituent with its host in the syntax in the corresponding non-coordinate sentence' (p. 156). Assuming that a possible entailment of this condition is that a postlexical clitic that cannot be repeated on each conjunct does not form a constituent with its host, one possible interpretation of (55c) could be that the Ezafe is in fact a (simple) postlexical clitic forming a constituent with the element on its right but attaching phonologically to the left. Though this is clearly not the option defended in this paper for the Ezafe in Modern Persian, the historical origin of the Ezafe as a clitic could provide insight into its paradoxical behavior with respect to coordination. Recall that it originated as a relative/demonstrative pronoun, i.e. originally it was not an affix. The affixal interpretation of the Ezafe in Modern Persian thus presupposes a long process of grammaticalization, with a number of intermediate stages. It could be assumed that the impossibility of the Ezafe being repeated on each conjunct is a remnant of its former status as a clitic.

From the body of evidence discussed in this section, especially the data involving haplology phenomena, it can be concluded that the Ezafe, the determiner *-i* and personal enclitics are best regarded as phrasal affixes in Persian. The Ezafe affix adjoins to any nominal non-maximal projection and registers the presence of a syntactic dependent, a modifier or a single NP complement, within phrases headed by a nominal category (nouns, adjectives and nominal prepositions).

5. A MORPHOLOGICAL ACCOUNT OF THE EZAFE IN HPSG

This section provides a formal account of the Ezafe construction within Head-driven Phrase Structure Grammar (HPSG), a constraint-based model developed in Pollard & Sag (1994) and Ginzburg & Sag (2000). I will first address the issue of whether these phrasal affixes are to be dealt with by word-level morphology as suggested by Zwicky (1987) and Miller (1992), or rather by postlexical phrasal morphology as proposed by Anderson (1992) and Anderson (2005). After arguing in favor of the first option, I will then present a treatment inspired by Miller (1992) and Tseng (2003), particularly in the use of EDGE feature percolation.

This content downloaded from 143.167.2.135 on Thu, 19 Nov 2015 22:53:22 UTC
All use subject to JSTOR Terms and Conditions

5.1 *Technical treatments of phrasal affixes*

Different technical treatments have been proposed in the literature to account for the specificities of phrasal affixes with respect to both head inflectional affixes and postlexical clitics (or bound words). I will consider three of these treatments. The first, proposed by Nevis (1988), can be considered a syntactic treatment, since not only do syntactic operations determine the placement of phrasal affixes, but the affixes correspond to a syntactic node. The other two treatments, provided by Zwicky (1987) and Miller (1992), on the one hand, and Anderson (2005) on the other hand, can be characterized as morphological treatments, though they vary with respect to the type of morphology involved in each case. After presenting a brief survey of each approach, I will argue in favor of the morphological treatment proposed by Zwicky (1987) and Miller (1992).

Nevis (1988) suggests treating phrasal affixes as clusters of features that must be positioned by a syntactic operation. This consists in a rule 'which has the effect of creating the equivalent of a grammatical function word, yet at the same time Chomsky-adjoining it to its lexical host' (p. 87). In other words, the lexical category X° which carries the relevant feature corresponding to a phrasal affix is expanded as a node X° and another node carrying the feature of the phrasal affix. One effect of this treatment is that phrasal affixes occur closer to their host than bound words do, but farther from their host than inflectional affixes. This accounts for their greater potential to interact morphophonologically with their host than is the case for bound words.

Zwicky (1987) claims that nothing in Nevis's framework requires that phrasal affixes, merely because of their location on the edge of a phrase, must necessarily receive a syntactic rather than a morphological account, and he argues instead in favor of assimilating phrasal affixes to the class of inflections. His claim basically relies on the haplology phenomena involving the suppression of the English POSS in some contexts. Zwicky notes that this suppression is not phonologically conditioned, since POSS is not suppressed in the context of nouns ending in one of the sibilants /z/ or /s/, as illustrated by 'the fuzz's old cars' and 'the bus's doors', while it is suppressed in the presence of other /z/ affixes. Consequently the rule realizing POSS must have access not only to the phonological shape and/or the morphosyntactic features of the host but also to its specific morphological composition. This is problematic, since although lexical phonological shape can be conditioned by properties of adjacent words, such conditioning is blind to their internal morphological composition. The problem is avoided if POSS is realized by the same sort of morphological rule as is used for the realization of standard inflectional affixes.

One consequence of the purely inflectional analysis is that every word in the English lexicon (not just nouns) would have at least one inflected form,

This content downloaded from 143.167.2.135 on Thu, 19 Nov 2015 22:53:22 UTC
All use subject to JSTOR Terms and Conditions

namely for the POSS affix. Nevertheless, Zwicky claims this move would represent no complication of the lexicon, since the shapes of the inflected forms are completely predictable from general principles.

Building on Zwicky (1987), Miller (1992) provides a morphological treatment of certain French function words (e.g. prepositions and determiners) within GPSG. Following in the same vein, Tseng (2003) proposes an analysis of the French definite article and the prepositions *de* and *à* as phrasal affixes handled morphologically within HPSG.

For Anderson (2005), special clitics constitute the morphology of phrases and can be regarded as 'the phrasal analog of (word-level) morphology' (p. 3). Unlike word-level morphology, however, phrasal morphology is introduced into the structure postlexically, as phonological modifications to the shape of phrases (p. 34). As a consequence, phrasal morphology has access only to syntactic structure and not to individual word-level properties, and thus cannot make item-specific modifications of form or content. Contra Zwicky (1987), who argues that the correct realization of English POSS requires access to the morphological structure of the host, Anderson claims that the syllabic structure of the host, which is accessible postlexically, is sufficient to handle the cases of POSS suppression. It is assumed that /z/ in this case 'is introduced not as a daughter of the syllable to which it is attached, but rather as an adjoined syllabic affix' (p. 93). Suffixal /z/ thus being structurally distinct from stem-final /z/, a haplology rule can be formulated that reduces two identical syllabic affixes to one.

To sum up, of the three treatments under discussion, only Zwicky's and Miller's approach provides access to individual word-level properties of the host for the realization of phrasal affixes. This is one of the reasons why it will be preferred here to Anderson's phrasal morphology approach. Indeed, the data discussed in the previous section provide rather clear evidence against a postlexical treatment of phrasal affixes in Persian, be it syntactic or morphological. Recall that the attachment of personal affixes to certain prepositions is exclusively lexically conditioned. Therefore, the decision whether a personal affix can be attached to a given preposition cannot be taken without knowledge of the individual word-level properties of the latter, nor can the morphophonological consequences of this attachment be predicted. Likewise, as far as I can see, Anderson's approach cannot accommodate the haplology phenomena involving the three types of phrasal affixes under discussion, since the impossibility of the latter cooccurring cannot be formulated in terms of phonological syllabic constraints.

Finally, Anderson's approach draws a rigid boundary between word-level inflectional affixes on the one hand and phrasal affixes on the other. Such a clear-cut separation leaves no room for affixes with a mixed status, whereas it seems to me that the three types of enclitics under discussion do display such a mixed status. For instance, the Ezafe, unlike 'standard'

This content downloaded from 143.167.2.135 on Thu, 19 Nov 2015 22:53:22 UTC
All use subject to JSTOR Terms and Conditions

phrasal affixes, does not attach to maximal projections, but to intermediate projections, including lexical heads. In this case, it acts very much like a lexical affix, indicating that the head, which is always a word, is followed by a modifier or a complement. We have seen that the determiner *-i* also has a dual behavior with respect to its placement properties: it either attaches to an intermediate nominal projection (i.e. the last modifier in the Ezafe domain), as in *xâne-ye digar-i* 'another house', or to the head noun, as in *xâne-i digar* 'another house'. Like the Ezafe, in the latter case, it is comparable to a head inflection.[14] On Zwicky's approach, these facts are not surprising, since phrasal affixes and word-level affixes are the same kind of objects.

5.2 *Phrasal affixes and EDGE features*

Relying on the idea of EDGE features introduced by Klavans (1985) to determine clitic placement, and its GPSG formalization by Miller (1992), Tseng (2003) develops an analysis of the French definite article and the prepositions *de* and *à* as phrasal affixes within HPSG. The basic idea is to handle the mismatch between the morphological scope of the phrasal affixes, i.e. a single word, and their syntactic and semantic scope, i.e. a phrase, by a treatment which is split between two levels. Affixation is handled by morphological rules in the lexicon, while interpretation is handled by unary syntactic rules. The link between the two levels is ensured by information encoded in EDGE features, which propagates from peripheral daughters to the mother in all branching syntactic combinations. Since the Ezafe and other phrasal affixes such as the article *-i* and possessive affixes always occur on the right periphery of the constituent they attach to, the use of EDGE features seems appropriate for handling them. At the first step, phrasal suffixation applies freely to existing words, creating different kinds of suffixed words: Ezafe-suffixed words, personal-affix-suffixed words and *i*-suffixed words. Leaving aside personal affixes and the indefinite *-i*, I will focus on the Ezafe in the remainder of this section.

As discussed earlier, the syntactic and semantic contribution of phrasal affixes cannot always be integrated at the point where suffixation is realized. The immediate effect of a phrasal affix on the lexical item it is adjoined to is to add a positive EDGE specification on the latter. Concretely, the effect of Ezafe suffixation may be encoded by a boolean feature [+EZ]. The morphological rule of Ezafe suffixation applies to a [−EZ] word and switches its specification to [+EZ]. Recall that the Ezafe has a dual behavior: when added to the head (noun, adjective or nominal preposition), it functions as a

[14] The necessity of allowing such hybrid affixes has also been noted in other studies. See, for instance, Crysmann (2003) and Luis & Spencer (2004) for pronominal clitics in European Portuguese.

This content downloaded from 143.167.2.135 on Thu, 19 Nov 2015 22:53:22 UTC
All use subject to JSTOR Terms and Conditions

word affix, which means that its syntactic contribution can only be integrated immediately, by the suffixation rule itself. When added to a modifier, it functions as a phrasal affix and thus its syntactic contribution must be integrated once the phrase is made up. In this case, (right) EDGE feature specifications are percolated from the rightmost branch up to the mother, until a phrase that the Ezafe could have scope over (i.e. an adjectival or prepositional phrase) becomes available. Then, a unary syntactic rule applies and incorporates the syntactic effect of the Ezafe into the phrase. Once the Ezafe interpretation rule is applied, the positive EDGE value for the Ezafe is removed in order to prevent its percolation to a still higher node.

The two-step analysis of the Ezafe for (56) is given in (57):[15]

(56) mojgân-e az rimel sangin-e maryam
eyelid.PL-EZ of mascara heavy-EZ Maryam
'Maryam's mascara-laden eyelids'

(57)

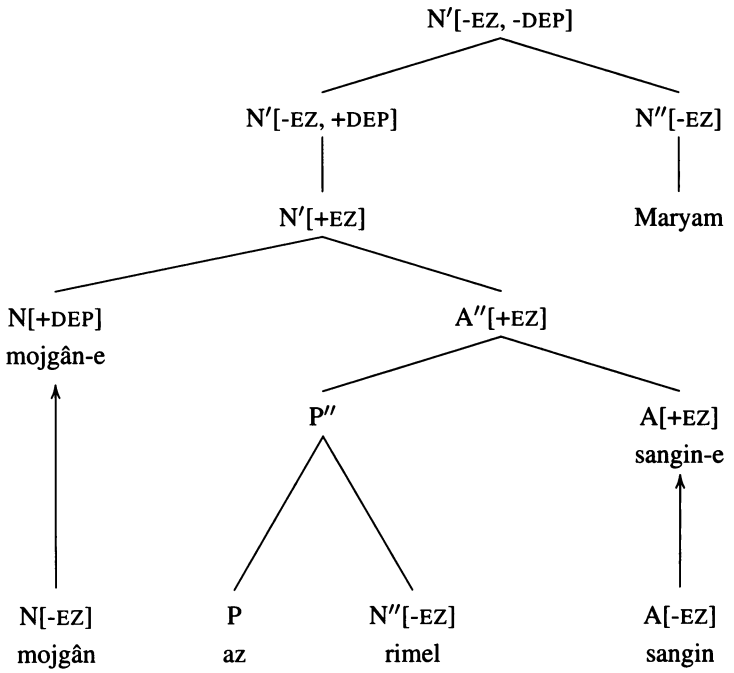

Lexical rule application (i.e. Ezafe suffixation) is indicated by the arrows. Here the rule applies twice, once on *mojgân* 'eyelid', which is the head of the

[15] This tree implies that modification takes place prior to complementation. The adjectival phrase adjoins first to an N° forming an N′ and then the possessor complement is adjoined to this N′ to give another N′. The motivation as well as the consequences of this analysis will be discussed later.

This content downloaded from 143.167.2.135 on Thu, 19 Nov 2015 22:53:22 UTC
All use subject to JSTOR Terms and Conditions

NP, and once on *sangin* 'heavy', which is the rightmost element within the adjectival phrase that precedes the Possessor NP. In the first case, the syntactic effect of the Ezafe may be integrated immediately on the head noun. This is done by the feature [DEP], which will be explained in the following paragraph. In the second case, by contrast, the Ezafe's scope is the whole AP, and thus it cannot be interpreted immediately. A positive EDGE value for EZ is consequently introduced on *sangin* 'heavy', and is percolated along the right periphery until an N′ is available. A unary syntactic rule then applies and the effect of the Ezafe is integrated into N′.

The Ezafe does not alter the syntactic category of the constituent over which it has scope. A noun remains a noun and an AP remains an AP. The only syntactic contribution of the Ezafe, as mentioned previously, is to indicate that the constituent must be followed by a noun, an AP, a PP or an NP. In order to encode this information, a new Boolean feature, [DEP(ENDENT) +], is added to all nominal lexical categories (i.e. nouns, adjectives and prepositions) and to their projections. Since this feature is not passed on from the head daughter to the mother, it cannot be a HEAD feature. Consequently, it will be added to CAT features, at the same level as HEAD and VAL(ENCE) features. For *lexemes*, the value of DEP is always negative, since *lexemes* have not undergone lexical rules. For *words* and *phrases*, it is negative by default, unless the Ezafe suffixation rule or the Ezafe interpretation rule has switched the negative value to a positive one.

5.3 *Lexical suffixation rules*

Ezafe suffixation is handled by lexical rules. These rules are of the *word-to-word* type: their input as well as their output is a *word* and they apply optionally. I will postulate two Ezafe suffixation rules.

The first rule, stated in (58), applies when the Ezafe is adjoined to the lexical head within an NP, an AP or a PP. As noted previously, the scope of the Ezafe in this case is always a word and its effect may be integrated immediately. Thus, there is no need to appeal to EDGE features to percolate the information about the Ezafe up the syntactic tree.

(58) Ezafe suffixation rule (1)

$$\begin{bmatrix} \textit{pl-nom-wd} \\ \text{PHON}\ \boxed{1} \\ \text{CAT}\begin{bmatrix}\text{DEP} -\end{bmatrix} \end{bmatrix} \rightarrow \begin{bmatrix} \textit{ph-af-nom-wd} \\ \text{PHON}\ \text{F}_{\text{EZ}}\left(\boxed{1}\right) \\ \text{CAT}\begin{bmatrix}\text{DEP} +\end{bmatrix} \end{bmatrix}$$

The left part of the rule describes its input and the right part its output. The rule applies to a *pl-nom-word* (i.e. a plain nominal word, containing no phrasal affix) and turns it into a *ph-af-nom-word* (i.e. a nominal word containing a phrasal affix). The requirement that the input be a *pl-nom-word*

This content downloaded from 143.167.2.135 on Thu, 19 Nov 2015 22:53:22 UTC
All use subject to JSTOR Terms and Conditions

keeps the rule from applying to words already containing a phrasal affix, and thus prevents phrasal affixes from stacking with the Ezafe. The phonological form of the output is givenby FEZ, a morphological function that applies to a nominal base. This function is sketched in (59):

(59)

X	FEZ(*X*)
X ending with vowel ≠ [i]	*X-ye*
Otherwise	*X-e*

Finally, the rule switches the negative value of DEP to a positive value and the syntactic effect of the Ezafe is thus incorporated on the head.

The second Ezafe suffixation rule, stated in (60), applies only within noun phrases. It is used when the Ezafe links a N′ to a modifier or to the possessor NP:

(60) Ezafe suffixation rule (2)

$$\begin{bmatrix} \textit{pl-wd} & \\ \text{PHON } \boxed{1} & \\ \text{EDGE} & \begin{bmatrix} \text{EZ} & \textit{none} \\ \text{PAF} & \textit{none} \\ \text{INDEF} & \textit{none} \end{bmatrix} \end{bmatrix} \rightarrow \begin{bmatrix} \textit{ph-af-wd} & \\ \text{PHON FEZ}(\boxed{1}) & \\ \text{EDGE} & \begin{bmatrix} \text{EZ} & \begin{bmatrix} \textit{ph-af} & \\ \text{IN} & N'[\text{DEP} -] \\ \text{OUT} & N'[\text{DEP} +] \end{bmatrix} \end{bmatrix} \end{bmatrix}$$

The rule in (60) selects a *plain-word* as its input and produces a *phrasal-affix-word*. The same morphological function FEZ is used to adjoin the Ezafe. We shall now consider EZ, which was suggested previously to have a boolean value. The negative value of EZ is encoded by *none*, but the implementation of the positive value is more complex, as shown in (60). In this case, the value of EZ is of type *phrasal-affix*. Two *sign*-valued attributes IN and OUT are associated to the type *ph-af*; these encode the potential grammatical effects of the suffix. The IN value provides a description of the sort of constituent the Ezafe combines with, that is, an N′ [DEP–]. The OUT value provides a description of the sort of constituent that results from this combination, which is the same constituent with a positive value for DEP. Following Tseng (2003), it is assumed here that any feature not mentioned in the OUT specification is shared with the IN value.

5.4 *EDGE feature propagation and suffix interpretation*

EDGE features propagate from peripheral daughters to the mother in all branching syntactic combinations. A positive EDGE feature specification indicates the presence of a phrasal affix whose syntactic and semantic effects

This content downloaded from 143.167.2.135 on Thu, 19 Nov 2015 22:53:22 UTC
All use subject to JSTOR Terms and Conditions

have not yet been integrated into the phrase that the phrasal affix scopes over. This integration occurs only if an appropriate phrase (matching the phrasal affix's IN value) becomes available in the syntactic construction. As long as such a phrase is not available, positive EDGE specifications must remain visible in syntax, passing up from one level to another. This propagation cannot be handled by the HEAD Feature Principle, since the phrasal affix does not necessarily occur on the head daughter of a phrase. The EDGE Feature Principle is therefore introduced to take care of EDGE feature propagation. The following statement of this principle is borrowed from Tseng (2003), with slight modifications in order to adapt it to Persian data:

(61) (Right) Edge Feature Principle

$$\begin{bmatrix} \textit{phrase} \\ \text{ARGS}\left\langle [\,], [\,], \ldots \right\rangle \end{bmatrix} \Rightarrow \begin{bmatrix} \text{EDGE}\ \boxed{1} \\ \text{ARGS}\left\langle \ldots, \begin{bmatrix}\text{EDGE}\ \boxed{1}\end{bmatrix} \right\rangle \end{bmatrix}$$

ARGS is a *list*-valued feature. Its value lists all daughters of a *sign* according to their surface order. The last member of the ARGS list corresponds to the rightmost branch. The EDGE feature principle requires that in any branching syntactic structure, the EDGE features of the right-peripheral daughter be shared with the dominating phrase.

The interpretation of phrasal affixes is handled by unary syntactic rules. These rules can be formulated as rewrite rules. The following rule is used for the Ezafe:

(62) Ezafe interpretation rule

$$\boxed{1}\text{N}'\begin{bmatrix} \text{CAT|DEP}\ + \\ \text{EDGE|EZ}\ \textit{none} \end{bmatrix} \rightarrow \boxed{2}\text{N}'\begin{bmatrix} \text{CAT|DEP}\ - \\ \text{EDGE|EZ}\begin{bmatrix}\text{IN}\ \boxed{2} \\ \text{OUT}\ \boxed{1}\end{bmatrix} \end{bmatrix}$$

The left-hand part of the rule describes the dominating node. The right-hand part contains a cyclic structure, since the unification of a phrase, namely an N′, with its own IN value is the condition for activating the syntactic contribution of EZ. Once EZ is interpreted, its value becomes *none* on the resulting phrase. All other IN and OUT features are identified by default. This is done by the Generalized Head Feature Principle (Ginzburg & Sag 2000). Thus, the mother N′ in the rule stated above shares all the features of the daughter N′, unless there is an explicit specification of a different value for a specific feature.

5.5 *Phrasal descriptions for NP and constraints on [DEP+]*

As was said earlier, the analysis of the Ezafe construction sketched in this paper implies that the possessor NP is a complement rather than a specifier,

This content downloaded from 143.167.2.135 on Thu, 19 Nov 2015 22:53:22 UTC
All use subject to JSTOR Terms and Conditions

as suggested by Ghomeshi (1997b). Assuming that the noun is the head of the nominal projection, two options are available for the possessor NP: either it is a complement or a specifier. Various versions of X-bar analysis rely on the assumption that a projecting head combines with its complements before combining with adjuncts (or modifiers). Within HPSG, this assumption is realized by constraints on phrases. In the following rule, which concerns phrases composed of a Head (Daughter) and its complements, the former is required to be a *word*:

(63) *head-complement-phrase* (Ginzburg & Sag 2000: 364)

$$[\,] \rightarrow \mathbf{H}\begin{bmatrix} \textit{word} \\ \text{COMPS } \textit{nelist}\left(\boxed{\text{A}} \oplus \textit{list}\right) \end{bmatrix} \boxed{\text{A}}$$

The fact that in Persian the possessor NP is required to occur after all adjectival modifiers could be an argument against its analysis as a complement, but rather as a specifier. The specifier analysis would imply that Persian nouns systematically lack complements, an option adopted by Sadler (2000) for the Welsh NP, which displays the same word order as Persian.[16] Moreover, it would provide a straightforward account for the word order pattern within the NP. Finally, it would account for the alleged definiteness of NPs containing a possessor NP (cf. Ghomeshi 1997b).

In spite of these facts, I will adopt the complement analysis of the Possessor NP, for the three following reasons:

(a) It provides a unified account of nouns, adjectives and P2 and P3 prepositions. Recall that the Ezafe construction is also encountered within adjectival phrases and prepositional phrases headed by a P2 or a P3. In this case, the Ezafe introduces the single complement of the lexical head (cf. Section 2). The complement analysis of the possessor NP allows for a unified account of an NP embedded within an NP, an AP or a PP, which will be considered as a complement in all cases.

(b) It provides a unified account of the role of personal affixes. Like the possessor NP, the NP complement of adjectives and prepositions may alternate with a personal affix: *negarân-aš* 'worried about her/him',

[16] In fact Sadler (2000) adopts the DP analysis: the possessor NP (DP) is in [Spec, NP] position, and D° takes the whole NP as its complement. The noun phrase in Welsh displays the same word order pattern as in Persian. Furthermore, overt determiners and possessor phrases are in complementary distribution in Welsh and, in the possessor construction, the definiteness specification for the entire noun phrase comes from the possessor phrase. Sadler argues that the apparent word order paradox, with specifiers intervening between head and complements, and adjectives between head and specifier, follows not from head movement, as has been assumed in various studies, but from the fact that nouns in Welsh systematically lack structural complements. Thus the 'semantic complements' are not syntactic complements at all, but adjuncts. See also Dobrovie-Sorin (2005) for a similar discussion.

This content downloaded from 143.167.2.135 on Thu, 19 Nov 2015 22:53:22 UTC
All use subject to JSTOR Terms and Conditions

ru-yaš 'on it'. Personal affixes are also used with verbs, in which case they always correspond to the first complement and never to the subject. The specifier analysis of the possessor NP would thus require two different analyses of the role of personal affixes with respect to a head. The complement analysis avoids this undesirable side effect.[17]

(c) It does not entail any commitment to a definite/presupposed reading for the whole NP. Contrary to Ghomeshi's (1997b) claim, Persian NPs containing a possessor NP are not obligatorily construed as definite, as illustrated by the following example:

(64) az pošt-e dar, sedâ-ye bačče âmad
from behind-EZ door, voice-EZ child come.PAS
'A/the child's voice was heard behind the door.'

In (64), *bačče* 'child', the 'possessor' NP, may receive either a definite/presupposed or a 'bare noun' interpretation. These two readings are clearly distinguished when the NP *sedâ-ye bačče* is the direct object of the verb:

(65) (a) sedâ-ye bačče šenid-am
voice-EZ child hear.PAS-1.SG
'I heard a child's voice.'
(b) sedâ-ye bačče râ šenid-am
voice-EZ child RÂ hear.PAS-1.SG
'I heard the child's voice.'

Since definite direct objects necessarily trigger RÂ (Lazard 1982, Karimi 1996, Ghomeshi 1997a and Samvelian 1997, among many others), the grammaticality of (65a) entails that an indefinite reading is available for *sedâ-ye bačče* 'child's voice'.

It may be objected that in (65a), *bačče* 'child' is not a possessor NP but an attributive noun, meaning 'childish', and thus resembling 'modificational genitives' (cf. Woisetschlaeger 1983 and Munn 1995) in English. Against this analysis it can be argued that *bačče* 'child' displays some important syntactic properties of a possessor NP. First, it is unique, which means that it cannot be followed by another possessor NP. Secondly, *bačče* 'child' occurs in the final position within the noun phrase and, unlike attributive nouns, cannot be followed by an adjective modifying the head noun *sedâ* 'voice', as is shown by the contrast between the following examples:

(66) (a) sedâ-ye boland-e bačče
voice-EZ loud-EZ child
'a child's loud voice'

[17] A similar argument has been used by Borsley (1995) to support the complement analysis of the possessor within the Welsh NP. The same view is adopted by Wintner (2000) for the Construct State nominal's associate in Modern Hebrew.

This content downloaded from 143.167.2.135 on Thu, 19 Nov 2015 22:53:22 UTC
All use subject to JSTOR Terms and Conditions

(b) *sedâ-ye bačče-ye boland
voice-EZ child-EZ loud
(putatively) 'a loud child's voice'

Finally, in such examples, the bare noun may in turn be followed by a 'possessor' NP linked by the Ezafe, without necessarily triggering a definite reading. This is particularly clear with infinitives, which can regularly head nominal projections in Persian:

(67) sedâ-ye šekastan-e šiše šenid-am
sound-EZ break.INF-EZ glass hear.PAS-I.SG
'I heard a sound of breaking glass./ I heard the glass breaking.'

Once again, considering *šekastan-e šiše* 'breaking of (a) glass' as a modifier leads to serious problems, given the fact that such an analysis in turn entails that *šiše* 'glass' is a modifier of *šekastan* '(to) break'.

For these reasons, the complement analysis of the possessor NP will be preferred to the specifier analysis. This means that the description for the *head-complement phrase* given in (63) must be replaced by a specific description for the NP, allowing the modifiers to be introduced either at the same time as or before the NP complement. The first option has in fact been adopted by certain studies within HPSG. Abeillé & Godard (2000), for instance, formulate a *hd-comp-adj-ph*, which allows complements and adjuncts (e.g. adjectives) to occur at the same level. However, this is not the option adopted here, as shown by (57). Instead, modifiers and the possessor NP are introduced at different stages. One argument in favor of this analysis is provided by coordination. Consider the following examples:

(68) (a) [lebâs-e sefid va kolâh-e zard]-e maryam
[dress-EZ white and hat-EZ yellow]-EZ Maryam
'Maryam's white dress and yellow hat'
(b) [livân-hâ-ye kristâl va bošqâb-hâ-ye čini]-e ru-ye miz
[glass-PL-EZ crystal and plate-pl-EZ porcelain]-EZ on-EZ table
'The crystal glasses and the porcelain plates on the table'

In (68a), the possessor NP *Maryam* can have wide scope over the coordination of two sequences formed by a (head) noun and an adjective. Given the fact that such a coordination is possible without the specific Right Node Raising intonation pattern, the sequence formed by the noun and the modifier (the adjective) can be assumed to form its own constituent. The same situation holds for (68b), where the PP modifier *ru-ye miz* 'on the table' can have wide scope over the coordination of two sequences formed by a (head)noun and an attribute noun.

This content downloaded from 143.167.2.135 on Thu, 19 Nov 2015 22:53:22 UTC
All use subject to JSTOR Terms and Conditions

On the basis of these observations, the description for *head-complement-nominal* phrases is stated as follows:

(69) *hd-comp-nom-ph*

$$[\,] \rightarrow \text{H} \begin{bmatrix} \textit{sign} \\ \text{HEAD } \textit{nominal} \\ \text{COMPS } \textit{nelist}\left(\boxed{\text{A}} \oplus \textit{list}\right) \end{bmatrix}, \boxed{\text{A}}$$

Note that the HEAD-DAUGHTER is not necessarily a *word* and consequently the noun can combine with its adjectival modifiers before combining with its complements.

In addition, the following constraint applies to *nominal* projections combining with an NP complement, and ensures that the *nominal* is Ezafe-marked in this case.

(70) $[\textit{nominal}, \text{COMP-DTRS} < \text{NP}, \ldots >] \Rightarrow [\text{DEP}+]$

This constraint, which concerns *nominal* heads (i.e. nouns, adjectives, P2 and P3 prepositions) and intermediate projections followed by an NP complement, rules out the following ill-formed examples:

(71) (a) *barâdar maryam
brother Maryam
(putatively) 'Maryam's brother'
(b) *šeydâ musiqi
fond music
(putatively) 'fond of music'

The *hd-adj-nom-ph*, stated in (72), allows a *nominal* to combine with an adjunct before combining with its NP complement. Since the rule may apply more than once, several modifiers may combine with a nominal head, whether lexical or phrasal.

(72) *hd-adj-nom-ph*

$$\begin{bmatrix} \text{HEAD-DTR } \boxed{1}\left[\text{HEAD } \textit{nominal}, \text{ DEP} +, \text{ COMPS}\left\langle [\,] \right\rangle\right] \\ \text{ADJ-DTR } \left[\textit{non-clausal}, \left[\text{MOD } \boxed{1}\right]\right] \end{bmatrix}$$

The fact that the *nominal* is required to bear a positive value for DEP rules out the following ill-formed examples:

(73) (a) *ketâb-hâ sabz
book-PL green
(putatively) 'green books'

This content downloaded from 143.167.2.135 on Thu, 19 Nov 2015 22:53:22 UTC
All use subject to JSTOR Terms and Conditions

(b) *lebâs-hâ bi astin
dress-PL without sleeve
'dresses without sleeve'

The restriction on the type of the ADJ-DTR (i.e. *non-clausal*) further rules out the possibility of a relative clause being linked to the head noun by the Ezafe.

Finally, the following constraint on *phrases* prevents [DEP +]-marked heads and constituents from occurring in final position, thus ruling out examples such as (75):

(74) Constraint on [DEP+]

$$\left[\textit{phrase}, \boxed{1}\text{HEAD-DTR}\left[\text{DEP}+\right]\right] \Rightarrow \left[\text{ARGS}\left\langle \ldots \boxed{1}, [\;] \ldots \right\rangle\right]$$

(75) (a) *[irâni-ân-e moqim-e farânse-ye] vâred šod-and
[Iranian-PL-EZ resident-EZ France-EZ] entered become.PAS-3.PL
(putatively) 'Iranians resident of France entered.'
(b) *maryam [zibâ-ye] ast
Maryam beautiful-EZ be.PRES
(putatively) 'Maryam is beautiful.'

Constraint (74) stipulates that, if within a *phrase* the HEAD-DTR is [DEP+], then it cannot be the last element on an ARGS list.

6. Conclusion

Previous works on the Ezafe construction in Persian have pointed out different kinds of restrictions on this construction and provided a syntactic account for them. In this paper, on the basis of numerous attested examples, I have first shown that a part of these restrictions, which have played a key role in the elaboration of the syntactic accounts, does not stand up to closer inspection. I have then argued that the morphological properties of the Ezafe, which may be viewed as an affix, and above all its interplay with other affixes belonging to the same class, such as personal affixes or the enclitic *-i*, can successfully account for the constraints on the Ezafe construction in Persian.

The analysis outlined in this paper further entails that the Ezafe particle, originating in the Old Iranian relative particle *-hya*, has undergone a long process of reanalysis-grammaticalization with some intermediate stages, to end up as a part of nominal (phrasal) inflection in Modern Persian. Under this account, the differences observed between related Iranian languages with respect to the Ezafe construction can be considered as a consequence of the fact that the grammaticalization process has presumably not proceeded to the same degree in all the daughter languages.

This content downloaded from 143.167.2.135 on Thu, 19 Nov 2015 22:53:22 UTC
All use subject to JSTOR Terms and Conditions

REFERENCES

Abeillé, Anne & Danièle Godard. 2000. French word order and lexical weight. In Robert D. Borsley (ed.), *The nature and function of syntactic categories* (Syntax and Semantics 32), 325–358. New York: Academic Press.

Anderson, Stephen R. 1992. *A-morphous morphology*. Cambridge: Cambridge University Press.

Anderson, Stephen R. 2005. *Aspects of the theory of clitics*. Oxford: Oxford University Press.

Bassols-Codina, Sergi. 1992. *La phrase relative en kurde central*. Doctoral dissertation, Université de Paris 3.

Bedir-Khan, Emir J. & Roger Lescot. 1970. *Grammaire kurde (dialecte kurmandji)*. Paris: Librairie d'Amérique et d'Orient.

Borsley, Robert D. 1995. On some similarities and differences between Welsh and Syrian Arabic. *Linguistics* 3, 99–122.

Crysmann, Berthold. 2003. Clitics and coordination in linear structure. In Birgit Gerlach & Janet Grijzenhout (eds.), *Clitics in phonology, morphology and syntax*, 121–159. Amsterdam: John Benjamins.

Darmesteter, James. 1883. *Etudes iraniennes* I. Paris: F. Vieweg.

Dobrovie-Sorin, Carmen. 2005. From DPs to NPs: A Bare Phrase Structure account of genitives. In Martine Coene & Yves d'Hhulst (eds.), *From NP to DP*, vol. 2: *The expression of possession in noun phrases*, 75–120. Amsterdam: John Benjamins.

Fattah, Ismaïl K. 2000. *Les dialectes kurdes méridionaux*. Leuven: Peeters.

Ghomeshi, Jila. 1997a. Topics in Persian VPs. *Lingua* 102, 133–167.

Ghomeshi, Jila. 1997b. Non-projecting nouns and the Ezafe construction in Persian. *Natural Language & Linguistic Theory* 15(4), 729–788.

Ginzburg, Jonathan & Ivan A. Sag. 2000. *Interrogative investigations*. Stanford, CA: CSLI Publications.

Haider, Hubert & Ronald Zwanziger. 1981. Relatively attributive: The 'ezâfe'-construction from Old Iranian to Modern Persian. In Jacek Fisiak (ed.), *Historical syntax*, 137–172. Berlin: Mouton.

Hincha, Georg. 1961. Beiträge zu einer Morphemlehre des Neupersischen. *Der Islam* 37, 136–201.

Holmberg, Anders & David Odden. 2005. The noun phrase in Hawramani. Presented at the First International Conference on Aspects of Iranian Linguistics, Leipzig, June 2005.

Kahnemuyipour, Arsalan. 2000. Persian Ezafe construction revisited: Evidence for modifier phrase. *The 2000 Annual Conference of the Canadian Linguistic Association Conference*, 173–185.

Karimi, Simin. 1996. Case and specificity: Persian *râ* revisited. *Linguistic Analysis* 26, 74–194.

Kent, Roland G. 1944. The Old Persian relative and aticle. *Language* 20, 1–10.

Klavans, Judith. 1985. The independence of syntax and phonology in cliticization. *Language* 61, 94–120.

Larson, Richard & Hiroko Yamakido. 2005. Ezafe and the Deep position of nominal modifiers. Presented at the Barcelona Workshop on Adjectives and Adverbs, March 2005.

Lazard, Gilbert. 1957. *Grammaire du persan contemporain*. Paris: Klincksieck.

Lazard, Gilbert. 1966. L'enclitique nominal *-i* en persan: un ou deux morphèmes? *Bulletin de la Société de Linguistique de Paris* 61, 249–264.

Lazard, Gilbert. 1982. Le morphème *râ* en persan et les relations actancielles. *Bulletin de la Société de Linguistique de Paris* 77, 177–207.

Lazard, Gilbert. 1992. *A grammar of contemporary Persian*. Costa Mesa: Mazda Publishers.

Lazard, Gilbert. 2006. *Grammaire du persan contemporain*. Tehran: Institut Français de Recherche en Iran.

Luís, Ana & Andrew Spencer. 2004. A paradigm function account of mesoclisis in European Portuguese. In Geert Booij & Jaap van Marle (eds.), *Yearbook of morphology*, 177–228. Dordrecht: Springer.

Mackenzie, David N. 1961. *Kurdish dialect studies*. London: Oxford University Press.

Miller, Philip H. 1992. *Clitics and constituents in phrase structure grammar*. New York: Garland.

Munn, Alan. 1995. The possessor that stayed close to home. In Vida Samiian & Jeannette Schaeffer (eds.), *The Western Conference on Linguistics (WECOL)* 24, 181–195.

Nevis, Joel A. 1988. *Finnish particle clitics and general clitic theory*. New York: Garland.

Nichols, Johanna. 1986. Head-marking and dependent-marking grammar. *Language* 62, 56–119.

This content downloaded from 143.167.2.135 on Thu, 19 Nov 2015 22:53:22 UTC
All use subject to JSTOR Terms and Conditions

Paul, Ludwig. 1998. *Zazaki*. Wiesbaden: Ludwig Reichert Verlag.
Perry, John R. 2005. *A Tajik Persian reference grammar*. Leiden: Brill.
Pollard, Carl & Ivan A. Sag. 1994. *Head-driven Phrase Structure Grammar*. Chicago, IL: The University of Chicago Press.
Sadler, Louisa. 2000. Noun phrase structure in Welsh. In Miriam Butt & Tracey Holloway King (eds.), *Argument realization*, 73–111. Stanford, CA: CSLI Publications.
Samiian, Vida. 1983. *Origins of phrasal categories in Persian: An X-bar analysis*. Ph.D. dissertation, University of California at Los Angeles.
Samiian, Vida. 1994. The Ezafe construction: Some implications for the theory of X-bar syntax. In Mehdi Marashi (ed.), *Persian studies in North America*, 17–41. Bethesda, MD: Iranbooks.
Samvelian, Pollet. 1997. La postposition *râ* en persan: ses liens avec la détermination et sa fonction discursive. *Cahiers de grammaire* 22, 187–231.
Samvelian, Pollet. 2006. L'enclitique *-i* introducteur de relative en persan: déterminant, allomorphe de l'ezâfe, ou autre chose encore? *Studia Iranica* 35, 7–34.
Schroeder, Christoph. 1999. Attribution in Kurmancî (Nordkurdisch). In Karl H. Wagner & Wolfgang Wildgen (eds.), *Studien zur Phonologie, Grammatik, Sprachphilosophie und Semiotik*, 43–63. Bremen: Institüt für Allgemeine und Angewandte Sprachwissenschaft.
Schroeder, Christoph. 2002. Zur Nominalphrasenstruktur des Kurmancî. In Manfred von Roncador, Wolfram Bublitz & Heinz Vater (eds.), *Philologie, Typologie und Sprachstruktur: Festschrift für Winfried Boeder zum 65. Geburtstag*, 191–210. Frankfurt: Peter Lang.
Stump, Gregory. 2001. *Inflectional morphology*. Cambridge: Cambridge University Press.
Tseng, Jesse. 2003. Phrasal affixes and French mophosyntax. In Gerald Penn, Gerhard Jäger, Paola Monachesi & Shuly Wintner (eds.), *Proceedings of Formal Grammar 2003*, 177–188. Stanford, CA: CSLI Publications.
Windfuhr, Gernot L. 1989. New West Iranian. In Rüdiger Schmitt (ed.), *Compendium Linguarum Iranicarum*, 251–262. Wiesbaden: Dr. Ludwig Reichert Verlag.
Wintner, Shuly. 2000. Definiteness in the Hebrew noun phrase. *Journal of Linguistics* 36(2), 319–363.
Woisetschlaeger, Eric. 1983. On the question of definiteness in 'an old man's book'. *Linguistic Inquiry* 14, 137–154.
Zwicky, Arnold. 1977. *On clitics*. Bloomington, IN: Indiana University Linguistics Club.
Zwicky, Arnold. 1985. Clitics and particles. *Language* 71, 283–305.
Zwicky, Arnold. 1987. Suppressing the Z's. *Journal of Linguistics* 23(1), 133–148.
Zwicky, Arnold & Geoffrey K. Pullum. 1983. Cliticization vs. inflection: English *n't*. *Language* 59, 502–513.

Author's address: Institut de Linguistique et de Phonétique Générales et Appliquées
Université de la Sorbonne Nouvelle – Paris III, 19, rue des Bernardins,
75005 – Paris, France.
E-mail: pollet.samvelian@univ-paris3.fr

This content downloaded from 143.167.2.135 on Thu, 19 Nov 2015 22:53:22 UTC
All use subject to JSTOR Terms and Conditions
