# Supplementary material for: Dependency Resolution Difficulty Increases with Distance in Persian Separable Complex Predicates: Evidence for Expectation and Memory-Based Accounts
Source: Front Psychol. 2016 Mar 30;7:403. doi: 10.3389/fpsyg.2016.00403 (PMC4812816; doi:10.3389/fpsyg.2016.00403)
Supplement: Supplementary file 1 [file DataSheet1.zip › SafaviEtAl2016DataCode/READMELongPP/UrduEzafe.pdf]

URDU EZAFE AND THE MORPHOLOGY-SYNTAX INTERFACE

Tina Bögel and Miriam Butt and Sebastian Sulger
Universität Konstanz

Proceedings of the LFG08 Conference

Miriam Butt and Tracy Holloway King (Editors)

2008

CSLI Publications

http://csli-publications.stanford.edu/

Abstract

This paper introduces data from Urdu into the discussion surrounding the well-known Persian *ezafe*-construction (Samvelian 2007, Gomeshi 1997, Samiian 1994) in order to further explore the interplay of phonology, morphology and syntax. In contrast to earlier studies of Samvelian (2007) for HPSG and Luís and Otoguro (2005) for LFG, who each introduce new formal mechanisms that map between the morphology and the syntax in order to resolve the tension between lexical/affixal properties of clitics (phrasal affixes) and their ability to take wide phrasal scope, our approach demonstrates that the classic LFG projection architecture already allows for a straightforward account of the properties of Urdu *ezafe* and postlexical clitics in general. In particular, we invoke postlexical prosodic phonology in order to ensure the correct placement of clitics, while accounting for their phrasal scope and lexical selectional properties in terms of c-structure representations and f-structure constraints, respectively.

1 Introduction

The aim of this paper is to introduce the hitherto undiscussed phenomenon of Urdu *ezafe* and to illustrate issues of the morphology-syntax-prosody interface with respect to clitics and phrasal affixes in general.[1] The *ezafe*-construction describes a dependency between a head noun and its modifiers in that it connects these modifiers to the head noun via the insertion of the *ezafe -e*. Samvelian (2007), who we take as a point of departure, follows Zwicky and Pullum (1983), Zwicky (1987) and Miller (1992) and analyzes the Persian *ezafe* as a phrasal affix that is part of the nominal morphology, which is introduced at the morphological level, but not postlexically as clitics are. We, on the other hand, propose that there is no real distinction between phrasal affixes and clitics. Phrasal affixes are those clitics which are on their way towards reanalysis as pieces of inflectional and derivational morphology. Within our understanding of grammar, clitics are not introduced postlexically but are independent lexical items, occupying their own leaf in the syntactic tree. The *prosodic* treatment of the clitics, however, occurs postlexically. Acknowledging the role of postlexical prosody in our view is imperative with respect to the *ezafe*-construction (and other clitics), because the proper integration of prosodic phonology into the architecture of grammar is what allows for a complete and straightforward analysis of the complex accumulation of behavioral properties of *ezafe* (and other clitics).

In section 2, we provide a brief overview on the Persian *ezafe* data in order to bring the reader up to date on the current discussion with respect to *ezafe*. An introduction to the Urdu data is given in section 3, followed by a Lexical-Functional Grammar (LFG) analysis in section 4. Although the Urdu *ezafe*-construction is not as complex as its Persian counterpart, our analysis allows us to revisit Samvelian's

[1]We would like to thank Tafseer Ahmed for help with and discussions of the relevant Urdu data.

analysis of *ezafe* as a phrasal affix in section 5, where we suggest that Persian *ezafe* could also be analyzed as a postlexial clitic. We conclude in section 6.

2 Persian *Ezafe*

Persian *ezafe* (from Arabic *iḍāfa* 'adjunction/addition') has been discussed extensively (Samiian 1983, 1994, Gomeshi 1997, Kahnemuyipour 2000, Holmberg and Odden 2005, Larson and Yamakido 2005, Samvelian 2007). An example is provided in (1), which illustrates that Persian *ezafe*, in contrast to the generally head-final nature of the language and NPs in particular, allows the head noun to be initial, with modifiers licensed to its right. The Urdu construction (section 3) functions similarly, however, the Persian *ezafe*-construction is much more complex in that modifiers can include adjectives, nouns and some PPs and that each of these in turn can function as the host for a further *ezafe*-construction. This stacking of *ezafe* is also illustrated by (1).

(1) [in ketâb]-e [kohne]-ye [bi arzeš]-e maryam
this book-Ez ancient-Ez without value-Ez Maryam
'this ancient worthless book of Maryam's' Persian
Samvelian (2007:606)

The *ezafe* originates from an Old Iranian relative pronoun -*hya*. In Middle Iranian, the pronoun evolved into *y/i* and became specialized as a device for nominal attribution. Samvelian (2007) argues that the *ezafe* was reanalysed further and has now become part of the nominal inflection. In order to provide the reader with the necessary overview, the following sections briefly summarize two major analyses of *ezafe* (Gomeshi 1997, Samvelian 2007).

2.1 Gomeshi's Analysis of the Persian *Ezafe*

The standard existing analysis for Persian *ezafe* is that of Gomeshi (1997), who builds on Samiian (1983). Gomeshi provides an X-bar account which involves non-projecting heads that may adjoin to each other. The *ezafe* never attaches to phrases, but selects as its domain the domains of X^0s or bare (lexical) heads. Gomeshi analyzes the *ezafe* as having no morphological status, but as being a sort of phonological linker inserted into the Phonological Form via an ***ezafe* Insertion Rule**. This rule attaches the *ezafe* -*e* to a lexical X^0 head bearing [+N].

2.2 Samvelian's HPSG-analysis of the Persian *Ezafe*

Samvelian (2007) reexamines the standard analysis of Gomeshi by providing new empirical facts and proposes an alternative analysis couched within Head-Driven Phrase Structure Grammar (HPSG). For the purpose of our analysis, we take Samvelian (2007) as a point of departure.

Samvelian demonstrates convincingly that the *ezafe* is not restricted to X^0s, but can attach to phrases as well. This is illustrated by (2), where the *ezafe*-licensed modifier of the head noun *Maryam* is phrasal.

(2) mojgân-e [az rimel sangin]-e maryam
eyelid.Pl-Ez of mascara heavy-Ez Maryam
'Maryam's mascara-laden eyelids' Samvelian (2007:635) Persian

Samvelian proposes that the *ezafe* is a *phrasal affix*, attaching to nominal heads or projections and marking them morphologically as expecting a modifier. In contrast to word-level inflectional affixes, which attach directly to their host and cannot be separated from them, *phrasal* affixes appear on the right edge of nominal constituents (usually non-maximal projections) and do not bear lexical stress. They are attached after the word-level affixes and cannot separate these from their hosts, as shown in (3).

(3) in pesar-hâ-ye/*pesar-ye-hâ ahmaq
this boy-Pl-Ez/boy-Ez-Pl silly
'these silly boys' Samvelian (2007:619) Persian

Samvelian also shows that phrasal affixes have wide scope over coordination. Miller (1992) establishes the coordination criterion in order to separate clitics from affixes: an element, which is unable to show wide scope over coordination and has to be repeated on each conjunct must have affixal status. However, Samvelian does not take this as a definitive argument in favor of a clitic status for *ezafe* since the argument works only towards ruling in affixes, but not necessarily ruling in clitics.

Samvelian's main argument why the *ezafe* does not have (postlexical) clitical status is because it is in complementary distribution with other phrasal affixes like the personal clitics and the determiner *-i* and can therefore be argued to involve haplology. The haplology criterion is a non-last-level phonological rule which also applies to *ezafe* and for that reason, Samvelian argues that *ezafe* cannot be a clitic (and have postlexical status), but must be a phrasal affix and as such part of word-level morphology, indicating grammatical (in this case dependency) relations.

We do not agree with Samvelian's analysis of *ezafe* as a phrasal affix and would like to suggest that she is forced into this analysis given the architectural assumptions of HPSG (we revisit the haplology criterion/argument in some detail in section 5). For example, Samvelian's analysis of (2) is illustrated in (4). The *ezafe* itself is introduced via two different lexical rules: 1) a word-to-word type; 2) a plain-word to phrasal-affix word. The word-to-word lexical rule allows the addition of *ezafe* to a word and produces a simple word (see *mojgân* in (4)). The second lexical rule allows *ezafe* to attach to a word that heads a phrase, as for *sangin* in (4). The consistent placement of *ezafe* at the right edge of a constituent is handled via an EDGE constraint first formulated by Miller (1992).

Once the *ezafe* has been added on to a word via one of the lexical rules, it then has the effect of introducing/licensing a dependent (DEP) to the right of the

head/phrase via a [+DEP] feature. When a dependent is not found in the immediate vicinity, the feature [+DEP] is carried along until a dependent is found to satisfy the [+DEP] requirement, at which point this feature is also reset to a negative value. The [+Ez] feature is set for *ezafe* on phrases and interacts with the EDGE constraint. The combined effect is to allow the *ezafe* to appear at the right edge of a phrase, while licensing the dependent via the head that may (as in (4)) or may not be embedded in a phrase.

(4) ↑= Lexical Rule Application

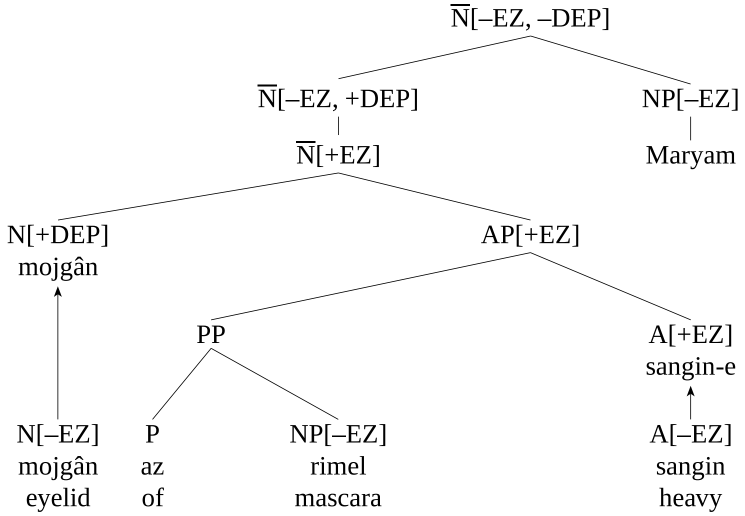

As already stated above, while we greatly appreciate Samvelian's insightful discussion and clarification of the facts, we would like to propose that constructions like the Persian *ezafe* do not necessarily need to be classified as a phrasal affix, but could be treated as a clitic. This can be done in an architecture which recognizes different levels of representations, or *projections*, as in LFG. We thus see the key to *ezafe* in recognizing a prosodic phonological component that interacts with syntactic structure at a level that goes beyond the definition and application of edge constraints. We would therefore not treat *ezafe* as part of the morphological component and indeed see no reason to do so. We present a concrete example of an analysis with respect to Urdu, for which we have an implemented grammar (Butt and King 2007) and for which we present the relevant facts in the next section.

3 Urdu *Ezafe* — A Brief Overview

This section provides a brief overview of the *ezafe*-construction in Urdu, which borrowed the construction from Persian (Platts 1909). Persian was the language of the Mughal court for several centuries and heavily influenced the language of the courtiers and poets at the court. Hence, the *ezafe* construction is still mainly part of the high/literary language. It remains productive today, but a tendency for modern speakers to leave it out can be observed (Schmidt 1999:247).

3.1 Headedness

As illustrated in (5) and as in Persian, the *ezafe -e* expresses a dependency between the head noun and a modifier to the right within the NP. This modifier can either be a noun as in (5a) or an adjective as in (5b); in the script, the expression can be either spelled out as one word or two words, where the *ezafe* is always attached to its host on the left.

(5) a. aarbaab=e k^hirad
owner=Ez wisdom
'wise person' Platts (1909:99) Urdu

b. sadaa=e buland
voice=Ez high
'a high voice' Delacy (2003:100) Urdu

As in Persian, the word order within the *ezafe*-construction is unusual for Urdu since its NP syntax otherwise conforms to the head-final pattern that is (almost) pervasive in this SOV language. For comparison, (6) illustrates a run-of-the-mill NP in Urdu.

(6) eek laal gaarii
one red car.F.Sg
'one/a red car' Urdu

The head-final pattern is also found in genitives, which are functionally/semantically related to the *ezafe*-construction. Example (7) demonstrates the common use of the genitive clitic *kii/kaa/kee*. As is usual for Urdu NPs, the head noun is in the final position of the NP while the modifier precedes the head. The genitive clitic inflects for gender and number and agrees with the head noun, see Butt and King (2005) and Payne (1995).

(7) paakistaan=kii hukuumat
Pakistan=Gen.F.Sg government.F.Sg
'Pakistan's government Schmidt (1999:246) Urdu

Compare the genitive in (7) with the functionally and semantically identical construction in (8), but which uses an *ezafe*. While the semantics are identical, the syntax clearly differs: as already established, the *ezafe* construction does not follow the head-final pattern observed in (7), but a head-initial pattern, the syntactic head being on the very left.

(8) huukumat=e paakistaan
government=Ez Pakistan
'the government of Pakistan' Schmidt (1999:246) Urdu

That the *ezafe* construction is indeed head-initial can be demonstrated via agreement facts. As shown in (9a) with respect to adjective agreement, the predicative adjective *baṛii* ‘big’ agrees with the feminine *vaadii* ‘valley’, just as it does in the simple predication in (9b).

(9) a. [**vaadii**=e sind^{h}] bahut **baṛ-ii** hai
valley.F.Sg=Ez Indus.M.Sg very big-F.Sg be.Pres.3.Sg
‘The Indus valley is very big.’ Urdu

b. **vaadii** bahut **baṛ-ii** hai
valley.F.Sg very big-F.Sg be.Pres.3.Sg
‘The valley is very big.’ Urdu

In a genitive NP, on the other hand, agreement clearly identifies the final noun as the head. This is illustrated in (10a) and (10b), the latter of which can be compared directly with (9a).

(10) a. naadyaa=kaa **kuttaa** bahut **baṛ-aa** hai
Nadya.F.Sg=Gen.M.Sg dog.M.Sg very big-Perf.M.Sg be.Pres.3.Sg
‘Nadya’s dog is very big.’ Urdu

b. [sind^{h}=kii **vaadii**] bahut **baṛ-ii** hai
Indus.M.Sg=Gen.F.Sg valley.F.Sg very big-F.Sg be.Pres.3.Sg
‘The valley of the Indus/Sindh is very big.’ Urdu

Like Persian, the Urdu *ezafe* construction is thus head-initial, rather than following the usual head-final pattern. And like Persian, it licenses a modifier/dependent to the right of the head. Unlike Persian, however, Urdu does not allow stacking of *ezafe* and only licenses either an adjective or noun as a modifier. The Persian construction is thus more complex, but as we are focusing on the question of affix vs. phrasal affix vs. clitic, the additional complexities of Persian are not relevant for the purposes of this paper.

3.2 Clitic vs. Affix

As already discussed, the morphological status of the *ezafe* itself is of particular interest in the context of this paper. At first glance, it might be a simple affix. However, unlike affixes and very much like clitics, the *ezafe* shows wide scope ((13)) and the head noun to which *ezafe* attaches can head a phrase ((11)) as well as be embedded within a phrase ((12)).

(11) [ye baṛ-aa diivaan]=e aam
this big-M.Sg hall of audience=Ez public
‘this big public hall of audience’ Urdu

(12) [[har baṛ-e diivaan]=e aam]=mẽ
every big-Obl hall of audience=Ez public=in
'in every big public hall of audience' Urdu

In (11) and (12), the *ezafe*-NP contains a determiner/quantifier and an adjective and is therefore clearly able to form a phrase over which the *ezafe* takes scope. The ability of *ezafe* to take wide scope is further supported by data from coordination, as shown in (13). Compare this to the example with a case clitic in (14), which has been established to have wide scope (Butt and King 2005, Mohanan 1994).

(13) [ye maal o daulat]=e dunyaa
this material and wealth=Ez world.Nom
'this material and wealth of the world' Urdu
(from *zarb-e-kaleem* by Muhammad Iqbal)

(14) [maal or daulat]=ko kumaa-o
material and wealth=Acc earn-Imp.Rude
'Earn/gather material and wealth!' Urdu

Morphological inflections, that is, common affixes like gender and number in Urdu, are not able to do this. The discrepancy between common inflections and *ezafe* and the resemblance of the *ezafe*-construction to the behavior of the well-known Urdu case clitics (Butt and King 2005, cf. Miller 1992) supports the analysis that the *ezafe* is also a clitic. In the next section, we present an analysis of Urdu *ezafe* as a clitic within LFG's standard projection architecture.

4 LFG Analysis of Urdu *Ezafe*

LFG is an inherently modular theory of grammar. The principle of *Lexical Integrity* defines word formation as being solely the domain of morphology, which interacts with *Lexical Phonology* (Kiparsky 1982). Anything that goes beyond the word level is the provenance of syntax, which builds phrases out of words and interacts with *Prosodic* and *Postlexical Phonology*. *Prosodic Phrasing* is part of *Prosodic Phonology* (Selkirk 1984, 1986, Nespor and Vogel 1986, Selkirk 1995), which we view as an additional module of the grammar. In our implementation we model this (as best as we can) via a *prosodic projection p* (Butt and King 1998).

Prosody is of great interest in the analysis of *ezafe*, because although clitics are considered to be "little words", they depend on a host. This is because clitics do not form a *prosodic* word of their own; they are prosodically deficient. Instead, they are phrased together with another prosodic word as part of the prosodic phrasing (prosodic phonology). Attaching them to their hosts is therefore not the business of morphology. The handling of so-called "special clitics" (Zwicky 1977) like the *ezafe*, which have a given, specialized syntactic distribution, in our opinion is the business of syntax. Since architectural questions appear to be of prime

importance in an analysis of clitics, we not only present a theoretical proposal for the treatment of *ezafe*, but model the phenomenon very concretely via a computational implementation, so that each module of the grammar and the interactions across modules are described concretely and precisely. The following subsections describe the modules involved, beginning with the morphological module.

4.1 Morphological Analyzer

For the morphological module of the current Urdu grammar, we use an autonomous finite-state analyzer (Beesley and Karttunen 2003), which is currently under development at Konstanz (Bögel et al. 2007). This analyzer takes a surface form and provides a morphological analysis in terms of abstract tags in combination with the word stem. A simple example with the word for *boy* is shown in (15), where the surface form is to the left and the morphological analysis is to the right.[2]

(15) `laRkA` ‘boy’ `laRk +Noun +Masc +Sg +Nom`

This abstract morphological analysis of a surface word is fed into the LFG grammar and is associated with appropriate f-structural information within the morphology-syntax interface (Kaplan et al. 2004). Essentially, each tag functions as a sublexical item with which f-structural information is associated. For example, the abstract morphological tag `+Sg` is “translated” for the purposes of the LFG grammar as the f-structural information (↑NUM) = sg.

In an *ezafe*-construction like *sher=e panjaab* ‘lion of Punjab’, *sher* and *panjaab* are nouns that are analyzed via the morphological analyzer, as shown in (16), which represents the actual (but for purposes of presentation somewhat simplified) output of our current morphological analyzer.

(16) `sher e panjAb` ‘lion of Punjab’

```
sher +Noun +Masc +Sg +Nom
e +Token
panjaab +Noun +Masc +Sg +Nom
```

Note that the *ezafe -e* is not dealt with by the morphological analyzer — it simply returns the information that this is a token, which simply means that the analyzer recognizes the *ezafe -e* as an independent element, not as a morphological affix. This is because we do not analyze the *ezafe* as an affixal morpheme and as such it is not dealt with within the morphological component. Rather, we treat it as an independent lexical element which we encode in the hand-written lexicon, along with other elements with special lexical and syntactic properties such as case clitics and auxiliaries (see (20)).

[2]Here we ignore the process of transliteration from the Urdu Arabic-based script to Roman characters, but see Bögel et al. (2007) for some discussion and references.

4.2 C- and F-structure Analyses

With respect to the phrase structure, we assume that the *ezafe* forms a constituent with the complement it introduces, motivated by its historical source as a relative clause (cf. section 2). This leads us to the phrase-structure analysis in (17), in which we posit an Ezafe Phrase (EzP), which is embedded in an NP and which functions as introducing an ezafe-licensed modifier to the head noun, which in turn is contained within an NP.[3]

(17) a. NPez $\longrightarrow$ NPez$'$ EzP

b. EzP $\longrightarrow$ Ez $\{$N | A$\}$

Our c-structural analysis for the sample *ezafe* construction *sher e panjaab* 'lion of Punjab' in (18a) is the c-structure tree in (18b).

(18) a. sher=e panjaab
lion=Ez Punjab
'a/the lion of Punjab' Urdu

b. C-structure Analysis

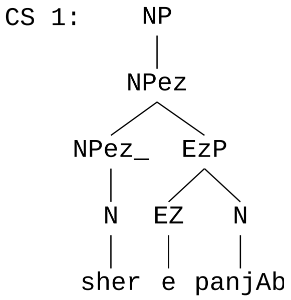

The EzP (*ezafe* Phrase) is headed by the *ezafe*, which takes the modifying noun as a complement and thereby licenses it. If there is no *ezafe*, there can be no modifier of the head noun.

This functional modification relation is represented at f-structure, as illustrated in (19). The head noun is *sher* 'lion' and therefore *sher* is encoded as the main predicate (PRED) of the construction. This main PRED is shown to be modified by the noun *panjaab*, which at c-structure is introcuded as the complement of *ezafe*. The presence of the *ezafe* is registered at the f-structure by means of a CHECK feature, which is generally used within the ParGram (Parallel Grammar) project (Butt et al. 2002) as a means for wellformedness, but has no further functional or semantic significance.

[3]Note that we could also simply assume a ternary-branching tree as in (i).

(i) NPez $\longrightarrow$ NP Ez $\{$N | A$\}$

However, we have chosen the binary branching tree since it demonstrates the mismatch across levels of representation much more clearly.

(19) F-Structure analysis of *sher e panjaab*

```
"sher e panjAb"
 PRED  'sher'
       PRED  'panjAb'
 MOD   NTYPE NSEM [COMMON count]
             NSYN common
     30 GEND masc, MOD-TYPE ezafe, NUM sg, PERS 3
 CHECK [_EZAFE +]
 NTYPE NSEM [COMMON count]
       NSYN common
1GEND masc, NUM sg, PERS 3
```

The lexical entry for *ezafe* in the hand-coded lexicon is provided in (20). All it says is that there is a modification and that its type is of the *ezafe* type.

(20) e EZ * (↑MOD MOD-TYPE) = ezafe.

Note that we have intentionally chosen an f-structural analysis that bears similarities to the standard ParGram treatment of compounding. This is because the Urdu *ezafe* does seem to be functionally similar to compounding in the sense that a modifier is placed in a range of semantic relations, possessive among them, to a head. However, since *ezafe* is not equivalent to compounding, we have registered the type of modification at hand via the MOD-TYPE feature.

4.3 P-structure Analysis

We now turn to the prosodic analysis of *ezafe*. That is, we need to be able to model the fact that the *e* is prosodically attached to the word on its left; in a phrase like *sher e panjaab* the intonational break is after the *ezafe* and not before it. This grouping with the word on its left is not accounted for by our c- and f-structural analysis since the *ezafe* forms a constituent with the modifier at c-structure and is barely represented at f-structure.

We propose to model the prosodic grouping of *ezafe* with the word on its left via the *prosodic projection* proposed by Butt and King (1998). As shown in (21), it is at this level of representation that the *ezafe* is grouped together with the word on its left (the head noun). That is, it is prosodically incorporated into the prosodic word on its left.

(21) Prosodic Structure

```
        83{-1[CL-FORM ezafe, DOMAIN P-WORD, P-FORM sher]}
       129{-2[DOMAIN P-WORD, P-FORM panjAb]}
135{131[DOMAIN P-PHRASE
```

As can be seen, the construction is analyzed as a prosodic phrase which consists of two prosodic words: *panjAb* ‘Punjab’ and *sher* ‘lion’. The *ezafe* is not an independent prosodic word (p-word) since as a clitic it is prosodically deficient. At p-structure, it is registered as [CL-FORM ezafe], where CL stands for “clitic” and is encoded having been incorporated into the domain of the p-word *sher*. The basic prosodic bracketing is also illustrated in (22).

(22) [[sher e] panjAb]

We have now accounted for all the properties of the *ezafe* construction. At c-structure, the *ezafe* functions as a head and licenses a complement to its right, thus introducing the modifier adjective or noun. The modification relation between this noun or adjective and the head noun is modeled at f-structure. To complete the picture, the prosodic relationship of the *ezafe* clitic with the head noun is expressed in (21) by combining them within one p-word at p-structure.

We therefore conclude that the analysis in terms of the independent modules of morphology (this includes lexical phonological processes), syntax and postlexical prosody provide exactly the right results for Urdu *ezafe*. Given our analysis, we can now revisit Persian *ezafe* to see if our basic approach can also apply to the more complex Persian case. But before turning to that in section 5, for the sake of completeness, we provide a comparison with the Urdu genitive construction, which bears a functional and semantic similarity to a subset of the *ezafe* constructions.

4.4 Comparing the Representation of the Genitive with *Ezafe*

Consider the genitive equivalent of *sher=e panjaab*, shown in (23). Here the head noun *sher* ‘lion’ is phrase final and the possessor (POSS) is licensed by the genitive case clitic *kaa*. Exactly this is modeled in (22).

(23) panjaab=kaa sher
Punjab=Gen.M.Sg lion.M.Sg
‘Punjab’s lion’ Urdu

(24) C- and F-structure analysis of the genitive construction *panjaab kaa sher*

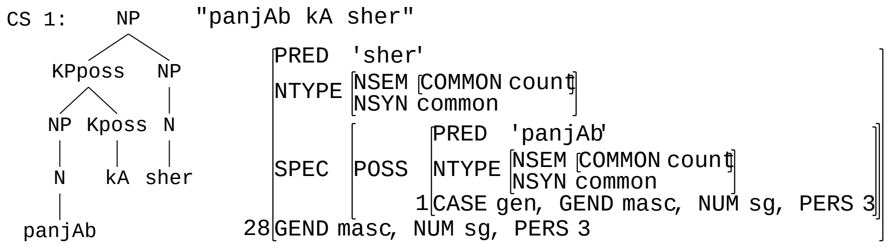

At c-structure ‘lion’ is the head of an NP, which includes an overtly case-marked possessive phrase called KPposs (for the use and justification of KP, see Butt and King 2005). The KPposs is headed by the genitive clitic, which introduces the possessor to its left. Unlike with the *ezafe*, there is no mismatch between syntactic and prosodic structure with respect to case clitics in general: they appear phrase finally in the syntax and are phrased together with the prosodic word on their left.

The f-structure analysis follows the standard ParGram LFG analysis of genitive possessors: the possessor is encoded under SPEC POSS, i.e., a specifier which provides further information about the head noun (*sher* ‘lion’ in (24)). Thus, while the c-structures of the genitive and the *ezafe* differ quite significantly, at f-structure both encode a modification relation — the genitive denotes a possessor relation, while we have analyzed *ezafe* as a semantically wider modification, resembling that found in compounding in other languages.

The example in (25) illustrates a combination of these two distinct syntactic constructions. As can be seen in (26), the head noun is again *sher* ‘lion’. It is modified by a possessor phrase (‘Lahore’) as well as by a noun (‘Punjab’) introduced through the *ezafe*. The *ezafe* itself functions as the head of its own syntactic projection, however, at p-structure it will be incorporated into the prosodic word to its left, namely *sher* ‘lion’.

(25) laahor=kaa sher=e panjaab
Lahore=Gen.M.Sg lion.M.Sg=Ez Punjab
‘Lahore’s lion of Punjab’ Urdu

(26) a. C-structure Analysis

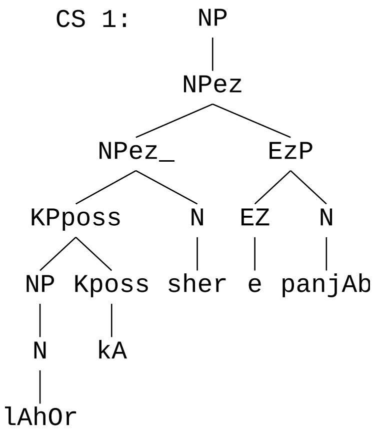

b. F-structure Analysis

```
"lAhOr kA sher e panjAb"

  ┌PRED   'sher'                                                              ┐
  │        ┌  ┌PRED  'panjAb'                                          ┐┐     │
  │MOD    {   │NTYPE ┌NSEM ┌COMMON count┐┐                             ││}    │
  │           │      └NSYN common        ┘                             ││     │
  │        └46└GEND masc, MOD-TYPE ezafe, NUM sg, PERS 3                ┘┘     │
  │CHECK  [_EZAFE +]                                                          │
  │NTYPE  ┌NSEM [COMMON count]┐                                             │
  │       └NSYN common         ┘                                             │
  │        ┌      ┌PRED     'lAhOr'                                             ┐┐
  │SPEC   │POSS  │NTYPE    ┌NSEM [PROPER [LOCATION-TYPE city, PROPER-TYPE location]]┐││
  │        │      │         └NSYN proper                                          ┘││
  │        │      │SEM-PROP [SPECIFIC +]                                           ││
  │        └     1└CASE gen, NUM sg, PERS 3                                        ┘┘
15└GEND masc, NUM sg, PERS 3                                                  ┘
```

Again, our analysis models exactly the right relationships at the right levels of analysis. We now examine Samvelian's analysis of Persian *ezafe* as a phrasal affix in some detail and suggest that given LFG's projection architecture, Persian *ezafe* could also be analyzed as a clitic along the lines of Urdu *ezafe*.

5 Revisiting Samvelian's Analysis

Samvelian (2007) analyzes the Persian *ezafe* construction as a *phrasal affix*. According to Anderson (2005), who assumes a Lexicalist Hypothesis (Anderson 1992), phrasal affixes (or Zwicky's 1977 *special clitics*) are a type of clitic which have restrictions as to which hosts they can appear with. Anderson calls these phrasal affixes the "morphology of phrases", because both derivational[4] and inflectional[5] phrasal affixes can be identified. Contra Samvelian, Anderson explicitly assumes prosodic phonology (Selkirk 1984, 1986, Nespor and Vogel 1986, Selkirk 1995) and sees phrasal affixes as being introduced postlexically. As modeled in our analysis above, clitics cannot be prosodic words on their own: they are analyzed as prosodically deficient and hence, have to be incorporated into the prosodic word or phrase of a host. We basically agree with Anderson's approach, except that we see the expression "morphology of phrases" as being about the *shape* of phrases and thus as falling squarely within the domain of syntax.

Anderson argues strongly against a syntactic approach to clitics. However, our approach does not presuppose movement for the positioning of clitics, which is what he objects to most (he proposes an Optimality Theoretic analysis in terms of alignment instead). Our analysis of Urdu *ezafe* would therefore seem to be in line with Anderson (2005), except that we see phrasal affixes as picking out those clitics

[4]Derivational clitics involve a modification of the meaning and/or discourse function, i.e. distinct interpretation of the form containing the clitic (="particles"); this could possibly include our *ezafe*.

[5]Inflectional clitics realize the phrasal properties of their domain, e.g., determiners/possessors within the NP.

which are in the process of being reanalyzed as derivational and inflectional morphology — a historical process which cannot be reflected directly in a synchronic model of grammar.

Returning to Samvelian (2007), her definition of phrasal affixes is quite different from Anderson's. She views phrasal affixes as occuring generally on the edge of a constituent, attaching to whatever word appears in this position. The phrasal affixes therefore exhibit a low degree of selection with respect to their host and resemble postlexically introduced clitics in many ways. However, she rejects Anderson's view that phrasal affixes come into play postlexically and analyzes Persian *ezafe* as part of word level morphology (following proposals by Zwicky 1987, Miller 1992) and distinguishes phrasal affixes from (postlexically introduced) clitics on this basis. As a proof for her theory she adduces three arguments:

1. Clitics do not have access to word-level properties.
2. The *haplology* criterion (Miller 1992, building on Zwicky 1987).
3. Anderson assumes a clear cut distinction between *word-level affixes* and *phrasal affixes*, but Persian *ezafe* would seem to be both at once.

The following sections take a closer look at each of these arguments.

5.1 Access to Word-Level Properties

We maintain that Samvelian's first argument is the product of fallacious reasoning about the architecture of grammars, namely that access to word-level properties of an entity can only happen within the morphological module. A quick comparison with other syntactic elements not involving clitics demonstrates this.

The word-level properties of *ezafe* need to access the class of part-of-speech that is involved, since it does not appear with just any kind of word, but is confined to adjectives, nouns and prepositions. However, exactly this kind of information is generally needed to ensure syntactic wellformedness. Consider non-clitic determiners, for example. These can never appear before verbs, but generally need to "look for" adjectives or nouns. Or consider the phenomenon of auxiliary stacking (cf. Chomsky's auxiliary hopping) in English: here each auxiliary in a chain like *John has been being seen* demands a certain morphological form on the verb or auxiliary it selects, otherwise the sentence is ill-formed. These are clearly both instances where syntactic processes must have access to word-level properties, but where one would not necessarily conclude that determiners or English auxiliaries should therefore be dealt with only within the prelexical morphological module.

5.2 The Haplology Criterion

Samvelian's use of the Haplology Criterion is taken from Miller (1992), who builds on Zwicky (1987). Haplology, as originally formulated in (27), has been used to

explain the fact that in the case of English nouns, multiple *'s* (plural/possessor) at the end of a word are supressed and only one *'s* remains, representing all others (Zwicky 1987, but also see Halpern 1995). Miller (1992) uses this as a proof of the treatment of phrasal affixes in the lexicon, because the applicability of Haplology would be hard to explain if the multiple *'s* were not added at the same (lexicon) level.

(27) **Haplology**
One syllable is deleted in the case of two identical syllables

However, as phonological processes can also apply as part of postlexical phonology, it is not clear to us why processes similar to Haplology, such as the fusion of multiple *'s* into one, should be taken as an indication of a process taking place in the prelexical morphological module (cf. Anderson 2005 for similar argumentation).

In any case, Samvelian (2007:627) builds on Miller's work and takes the Haplology Criterion to mean that one has to establish the following:

> (a) Elements are in complementary distribution (i.e., in competition) when adjoined to the right edge of the same constituent;
>
> (b) any sequence containing two or more of the same element is excluded, even when their scope is in the same constituent.

In particular, after taking some time to establish that the definite *=i* and the personal pronoun clitics are enclitics and are phrasal affixes under her definition, she compares their distribution with respect to *ezafe*. She finds that the definite *=i* and the personal pronouns are in complementary distribution/competition with *ezafe* and that there cannot be more than one of them attached to a phrase (or word). In (28a), for example, the *ezafe* is applied to the head noun, and the indefinite article is applied to the modifier. Surprisingly, in (28b), the position of the *ezafe* is taken over by the indefinite determiner, which appears on the head noun instead of the modifier. (28c) further shows that the indefinite determiner **has** to appear on the head noun instead of the modifier in order to take over the function of the *ezafe*. Otherwise, the sentence is ungrammatical.

(28) a. xâne-ye digar-i
house-Ez another-Indef
'another house' Samvelian (2007:627) Persian

b. xâne-i digar
house-Indef another
'another house' Samvelian (2007:627) Persian

c. *xâne digar-i
house another-Indef
(putatively) 'another house' Samvelian (2007:628) Persian

d. *xâne-i-e/e-i digar
house-Indef-Ez/Ez-Indef another
(putatively) 'another house' Samvelian (2007:627) Persian

Finally, in (28d), Samvelian demonstrates that the *ezafe* and the indefinite determiner cannot cooccur on the same word. Samvelian interprets these facts as due to a version of the Haplology Criterion, where the indefinite determiner is dominant over the *ezafe* and inherits its functions whenever it "suppresses" the *ezafe*. Since Samvelian has established that the indefinite =*i* is a phrasal affix, she concludes that the *ezafe* must also be a phrasal affix that cannot be introduced postlexically, but must be part of the prelexical morphological module, where it competes with the indefinite =*i*.

However, the question of dominance between the *ezafe* and the two other enclitics is not as clear anymore if we look at another of Samvelian's examples, shown in (29).

(29) lebâs-e sefid-e bi âstin-am/-i
dress-Ez white-Ez without sleeve-Pers.I.Sg/-Indef
'my/a white dress without sleeves' Samvelian(2007:621–622) Persian

Both the determiner -*i* and the personal pronoun -*am* are normally attached directly to the head noun they modify. However, in (29), the head noun *lebâs* is part of an *ezafe* construction and is marked with an *ezafe*. Instead of appearing in the normal position on the head noun and simply "suppressing" the *ezafe* as should be possible according to Samvelian's analysis, the enclitics select the only non-*ezafe*-marked modifier (*âstin*) as their new host. In this case, the *ezafe* seems to display some dominance, because the others will attach to the last (not *ezafe*-marked) modifier in case of an *ezafe*-construction.[6]

One could also argue that the *ezafe* and the indefinite determiner -*i* have overlapping functions and are therefore in complementary distribution on functional grounds. (Complementary distribution also seems to rule out a coocurrence of personal pronouns and clitics; see Samvelian (2007) for a detailed description.)

If we nevertheless consider complementary distribution (for whatever reason) as a criterion to establish prelexical morphological affixation, it is still not clear what speaks against the postlexical clitic analysis, because there are other well-known syntactic elements like indefinite vs. definite determiners or past vs. present tense auxiliaries that tend to be in complementary distribution. Furthermore, if there are clitics which fulfill a certain function or license modifiers (like the *ezafe* does), then it is not clear that they should be stackable with other clitics — this would depend on the syntactic construction and what the syntax of the language is

[6]In (29), the *ezafe* seems to banish the indefinite -*i* and the personal affixes to other positions. As Samvelian remarks in her section about these affixes, they are much more promiscuous with respect to their host than the *ezafe*. It is therefore logical that these two are more mobile and able to attach themselves to a new host while the *ezafe* stays in the position where it naturally licenses a following modifier.

able to license. Therefore we conclude that the haplology criterion is not a strong argument in favor of the lexical treatment of the *ezafe*.

5.3 Word-Level Affixes vs. Phrasal Affixes

Anderson (2005) assumes a clear cut distinction between *word-level affixes* and *phrasal affixes*, but according to Samvelian's analysis, which involves two types of lexical rules for *ezafe* (one word-to-word, the other word-to-phrase, cf. section 2), Persian *ezafe* is both at once. *Ezafe* also attaches to intermediate projections, but not maximal ones. She thus sees Persian *ezafe* as having mixed properties that cannot be accomodated within Anderson's system, in which phrasal affixes are always introduced postlexically.

However, this reasoning is not very strong, if one simply assumes that *ezafe* is syntactically placed in a certain syntactic position in order to license a modifier, as per our analysis in section 4, and then is prosodically incorporated into the prosodic word on its left in prosodic phonology. Using this solution, no special rules need to be formulated and *ezafe* is not any different from any other clitic with special syntactic distribution (Anderson's *phrasal affix*).

In addition, a very strong argument for the interpretation of the *ezafe* as a clitic is the coordination test. This is so even if one focuses exclusively on the Criteria formulated by Zwicky and Pullum (1983), which Samvelian relies on heavily. Their *Criterion E* states that no syntactic operation treats a word and a clitic as a unit, which would have to be the case with coordinated structures like the one in (30), where a coordination rule would have to treat *zard-e* 'yellow-Ez' as a unit.

(30) [[kolâh-e sefid](*-e) va [lebâs-e zard]]-e maryam
hat-Ez white(-Ez) and dress-Ez yellow-Ez Maryam
'Maryam's white hat and yellow dress' Samvelian(2007:630) Persian

Under our view, the inability of the adjective *sefid* to take an *ezafe* is because the *ezafe* is an independent syntactic element heading a constituent and introducing a complement (the *ezafe* modifier), but in terms of prosody is prosodically deficient and therefore must incorporate prosodically to the prosodic word on its left. Since clitics on the one hand are not repeated on each conjunct, and affixes on the other hand are supposed to be able to do so,[7] the analysis of the *ezafe* as a phrasal affix which is introduced prelexically in the morphological module is on shaky ground and an analysis as a clitic would seem to fit the facts better.

6 Conclusion

If one assumes a modular architecture of grammar in which phonology, morphology, syntax and prosodic phonology are all able to interact, a clean and straightforward analysis of both the Urdu and Persian *ezafe* constructions is possible. In

[7]Note the fact that personal pronouns and the determiner -*i* can attach to every conjunct.

particular one needs to acknowledge the role of prosodic phonology, formalized by us as a *prosodic projection*. With this solution one does not have to overload the morphology in order to deal with syntactic and postlexical prosodic phenomena (and thereby give up on the modularity of grammar.)

Under our analysis, the *ezafe* is treated as a lexical item in that it has its own lexical entry and is realized as a leaf in the c-structure tree. Its idiosyncratic distributional properties follow directly from the syntactic constraints as to where it can be placed and after what kinds of constituents (only nominal projections in Urdu). The ability of *ezafe* to take phrasal scope is also represented in the syntax, but the phonological/prosodic attachment to just one member of the constituent is realized postlexically in terms of the p(rosodic)-projection. That is, the *ezafe* is analyzed as being part of the same prosodic word as the preceding noun.

Based on our analysis and implementation of Urdu *ezafe*, we thus argue that there is no need to augment the existing architecture of LFG. Rather, if one analyzes/situates the right aspects of phenomena involving phrasal affixes/clitics at the right levels of representations, the existing architecture proves more than adequate.

References

Anderson, Stephen R. 1992. *A-Morphous Morphology*. Cambridge: Cambridge University Press.

Anderson, Stephen R. 2005. *Aspects of the Theory of Clitics*. Oxford: Oxford University Press.

Beesley, Kenneth and Karttunen, Lauri. 2003. *Finite State Morphology*. Stanford, CA: CSLI Publications.

Bögel, Tina, Butt, Miriam, Hautli, Annette and Sulger, Sebastian. 2007. Developing a Finite-State Morphological Analyzer for Urdu and Hindi. In *Proceedings of the Sixth International Workshop on Finite-State Methods and Natural Language Processing,* Potsdam.

Butt, Miriam, Dyvik, Helge, King, Tracy Holloway, Masuichi, Hiroshi and Rohrer, Christian. 2002. The Parallel Grammar Project. In *COLING Workshop on Grammar Engineering and Evaluation*.

Butt, Miriam and King, Tracy Holloway. 1998. Interfacing Phonology with LFG. In Miriam Butt and Tracy Holloway King (eds.), *On-line Proceedings of the LFG98 Conference*, CSLI Publications, University of Queensland, Brisbane.

Butt, Miriam and King, Tracy Holloway. 2005. The Status of Case. In Veneeta Dayal and Anoop Mahajan (eds.), *Clause Structure in South Asian Languages*, pages 153–198, Dordrecht: Kluwer Academic Publishers.

Butt, Miriam and King, Tracy Holloway. 2007. Urdu in a Parallel Grammar Development Environment. *Language Resources and Evaluation* 41, 191–207, Special Issue on Asian Language Processing: State of the Art Resources and Processing.

Gomeshi, Jila. 1997. Non-Projecting Nouns and the Ezafe Construction in Persian. *Natural Language and Linguistic Theory* 15(4), 729–788.

Halpern, Aaron. 1995. *On the Placement and Morphology of Clitics*. Stanford, CA: Stanford University.

Holmberg, Anders and Odden, David. 2005. The Noun Phrase in Hawramani, presented at the *First International Conference on Aspects of Iranian Linguistics*.

Kahnemuyipour, Arsalan. 2000. Persian Ezafe Construction Revisited: Evidence for Modifier Phrase. In *The 2000 Annual Conference of the Canadian Linguistic Association Conference*, pages 173–185.

Kaplan, Ronald M., Maxwell III, John T., King, Tracy H. and Crouch, Richard. 2004. Integrating Finite-State Technology with Deep LFG Grammars. In *Proceedings ESSLLI, Workshop on Combining Shallow and Deep Processing for NLP*.

Kiparsky, Paul. 1982. Lexical Morphology and Phonology. In *Linguistics in the Morning Calm*, pages 3–91, Seoul: Hanshin.

Larson, Richard and Yamakido, Hiroko. 2005. Ezafe and the Deep Position of Nominal Modifiers, presented at the *Barcelona Workshop on Adjectives and Adverbs*.

Luís, Ana R. and Otoguro, Ryo. 2005. Morphological and Syntactic Well-formedness: the Case of European Portuguese Proclitics. In Miriam Butt and Tracy Holloway King (eds.), *Proceedings of the LFG05 Conference*, CSLI Publications, University of Bergen.

Miller, Philip H. 1992. *Clitics and Constituents in Phrase Structure Grammar*. New York: Garland.

Mohanan, Tara. 1994. *Argument Structure in Hindi*. Stanford, CA: CSLI Publications.

Nespor, Marina and Vogel, Irene. 1986. *Prosodic Phonology*. Dordrecht: Foris.

Payne, John. 1995. Inflecting Prepositions in Indic and Kashmiri. In Frans Plank (ed.), *Double Case*, pages 283–298, London: Academic Press.

Platts, John T. 1909. *A Grammar of the Hindustani or Urdu Language*. London: Crosby Lockwood and Son, republished in 2002 by Sang-e-Meel Publications, Lahore.

Samiian, Vida. 1983. *Origins of Phrasal Categories in Persian: an X-bar Analysis*. Ph. D.thesis, University of California.

Samiian, Vida. 1994. The Ezafe Construction: Some Implications for the Theory of X-bar Syntax. In Mehdi Marashi (ed.), *Persian Studies in North America*, pages 17–41, Bethesda: Iranbooks.

Samvelian, Pollet. 2007. A (Phrasal) Affix Analysis of the Persian Ezafe. *Journal of Linguistics* 43, 605–645.

Schmidt, Ruth Laila. 1999. *Urdu — an Essential Grammar*. New York: Routledge.

Selkirk, Elizabeth. 1984. *Phonology and Syntax: The Relation between Sound and Structure*. Cambridge, MA: The MIT Press.

Selkirk, Elizabeth. 1986. On Derived Domains in Sentence Phonology. *Phonology Yearbook* 3, 371–405.

Selkirk, Elizabeth. 1995. The Prosodic Structure of Function Words. In J. Beckman, L.W. Dickey and S. Urbanczyk (eds.), *Papers in Optimality Theory*, pages 439–470, Amherst, University of Massachusetts Occasional Papers in Linguistics 18.

Zwicky, Arnold. 1977. On Clitics, Bloomington: Indiana University Linguistics Club.

Zwicky, Arnold. 1987. Suppressing the Z's. *Journal of Linguistics* 23(1), 133–148.

Zwicky, Arnold and Pullum, Geoffrey K. 1983. Clitization vs. Inflection: English n't. *Language* 59, 502–513.
