# Supplementary material for: Dependency Resolution Difficulty Increases with Distance in Persian Separable Complex Predicates: Evidence for Expectation and Memory-Based Accounts
Source: Front Psychol. 2016 Mar 30;7:403. doi: 10.3389/fpsyg.2016.00403 (PMC4812816; doi:10.3389/fpsyg.2016.00403)
Supplement: Supplementary file 1 [file DataSheet1.zip › SafaviEtAl2016DataCode/SPR/data-preparation/crit.docx]

Position of Critical Region

Persian e 1 (2,4,2,2 (overall 10) mispositions out of 144 sentences in latin square)

Condition a (light v) 🡪 4

Condition b (light v) 🡪 8

Condition c (heavy v) 🡪 4

Condition d (heavy v) 🡪 8

Exceptions :

13 d 🡪 9 / 19 b 🡪 9 / 15 c🡪 5 / 6 c 🡪 5 / 33b 🡪 9 / 19d 🡪 9 / 6d 🡪 9 / 26d 🡪 9

Persian e 2 (1,0,1,1 (overall 3) mispositions out of 144 sentences in latin square)

Condition a (light v) 🡪 4

Condition b (light v) 🡪 6

Condition c (heavy v) 🡪 4

Condition d (heavy v) 🡪 6

Exceptions :

6d 🡪 7 / 6b 🡪 7 / 12a 🡪 5
