# Supplementary figures and images for: Dependency Resolution Difficulty Increases with Distance in Persian Separable Complex Predicates: Evidence for Expectation and Memory-Based Accounts
Source: Front Psychol. 2016 Mar 30;7:403. doi: 10.3389/fpsyg.2016.00403 (PMC4812816; doi:10.3389/fpsyg.2016.00403)

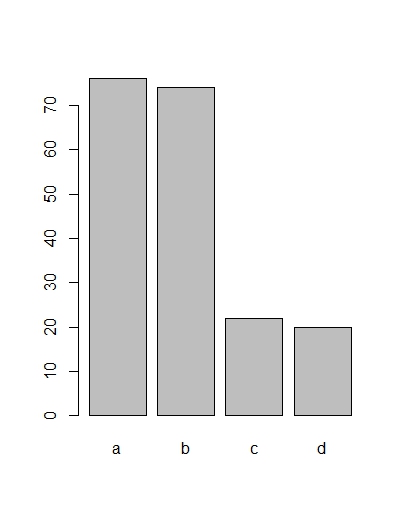

Supplement: Supplementary file 1 [file DataSheet1.zip › SafaviEtAl2016DataCode/Pretests/Pretest.plots/pretest1-plots/pretest 1, expt. 1.jpeg]

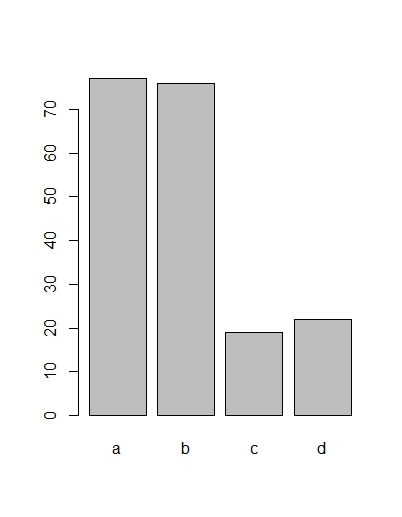

Supplement: Supplementary file 1 [file DataSheet1.zip › SafaviEtAl2016DataCode/Pretests/Pretest.plots/pretest1-plots/Pretest 1- expt. 2.jpeg]

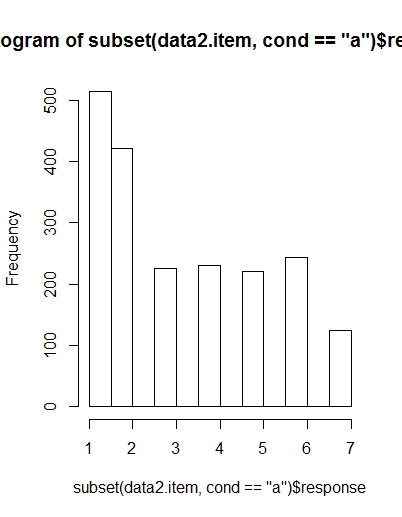

Supplement: Supplementary file 1 [file DataSheet1.zip › SafaviEtAl2016DataCode/Pretests/Pretest.plots/pretest2.plots/pretest 2 - cond a on 7 scales.jpeg]

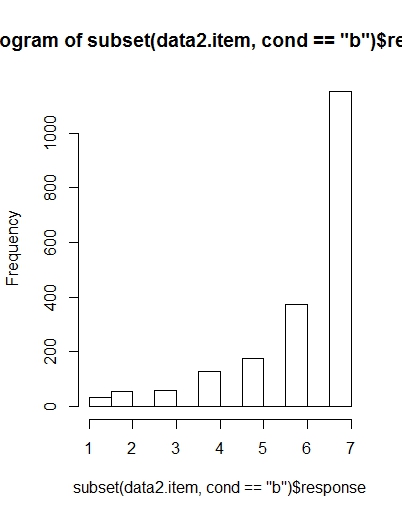

Supplement: Supplementary file 1 [file DataSheet1.zip › SafaviEtAl2016DataCode/Pretests/Pretest.plots/pretest2.plots/pretest 2 - cond b on 7 scales.jpeg]

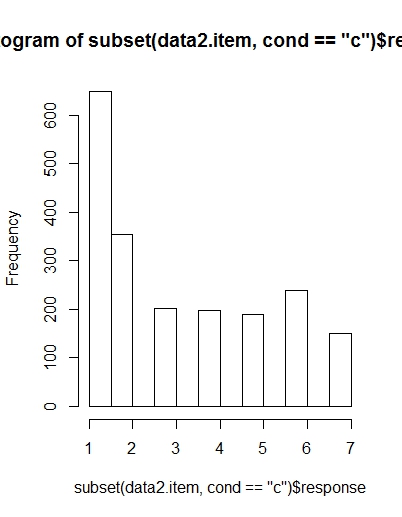

Supplement: Supplementary file 1 [file DataSheet1.zip › SafaviEtAl2016DataCode/Pretests/Pretest.plots/pretest2.plots/pretest 2 - cond c on 7 scales.jpeg]

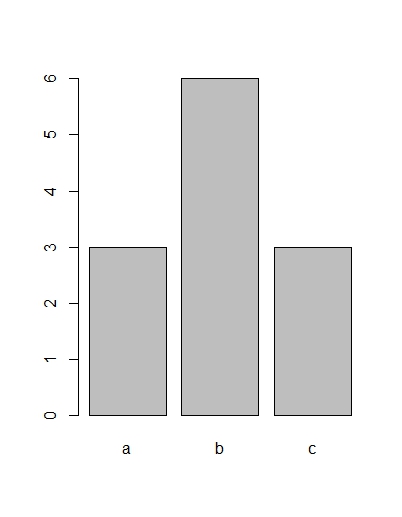

Supplement: Supplementary file 1 [file DataSheet1.zip › SafaviEtAl2016DataCode/Pretests/Pretest.plots/pretest2.plots/pretest 2 - conds.jpeg]

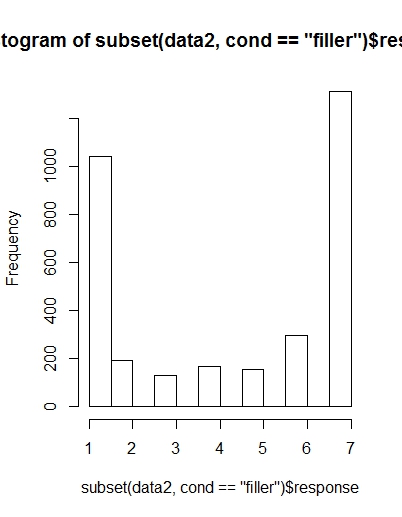

Supplement: Supplementary file 1 [file DataSheet1.zip › SafaviEtAl2016DataCode/Pretests/Pretest.plots/pretest2.plots/pretest 2 - fillers on 7 scales.jpeg]

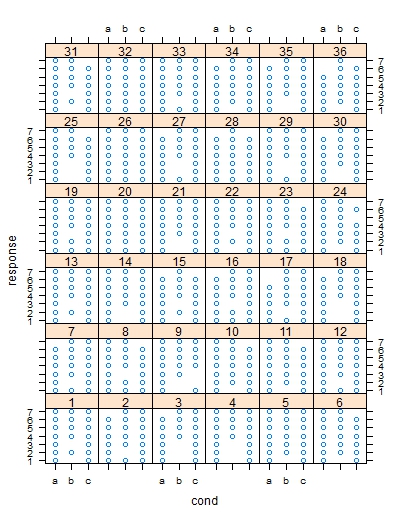

Supplement: Supplementary file 1 [file DataSheet1.zip › SafaviEtAl2016DataCode/Pretests/Pretest.plots/pretest2.plots/pretest 2 - xy plot - per participant.jpeg]

friend-ez sister-ez me

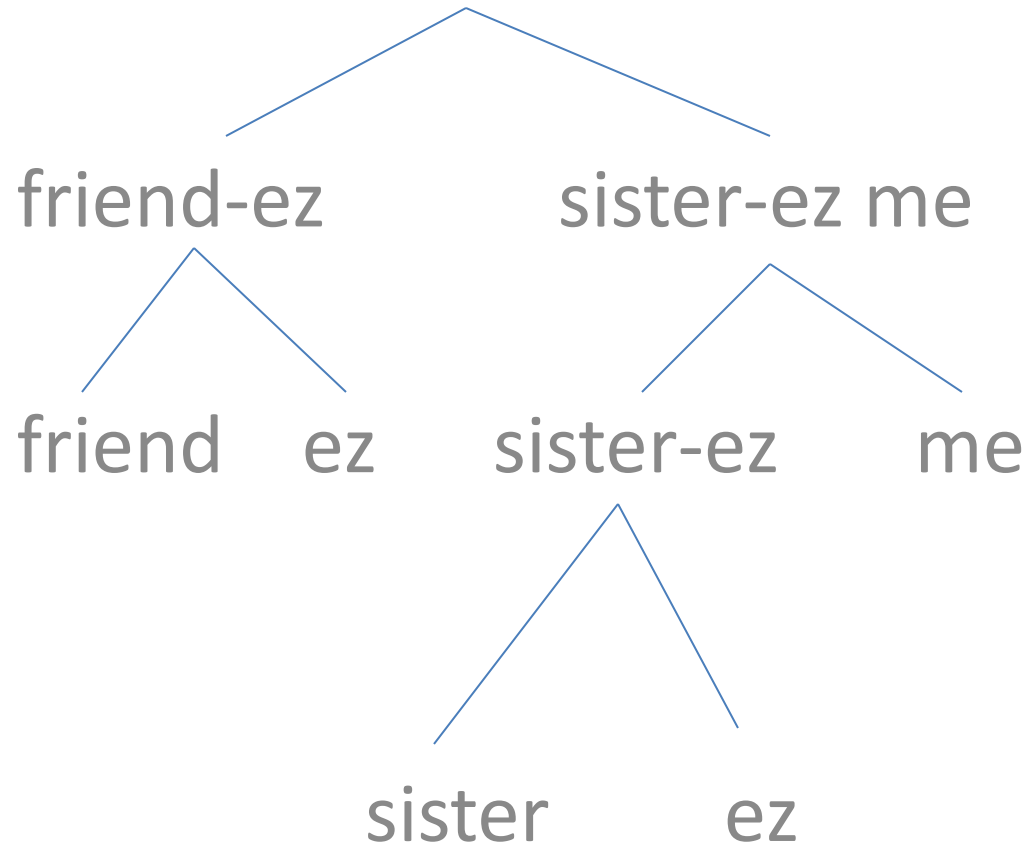

Supplement: Supplementary file 1 [file DataSheet1.zip › SafaviEtAl2016DataCode/READMELongPP/Binary tree.pdf]

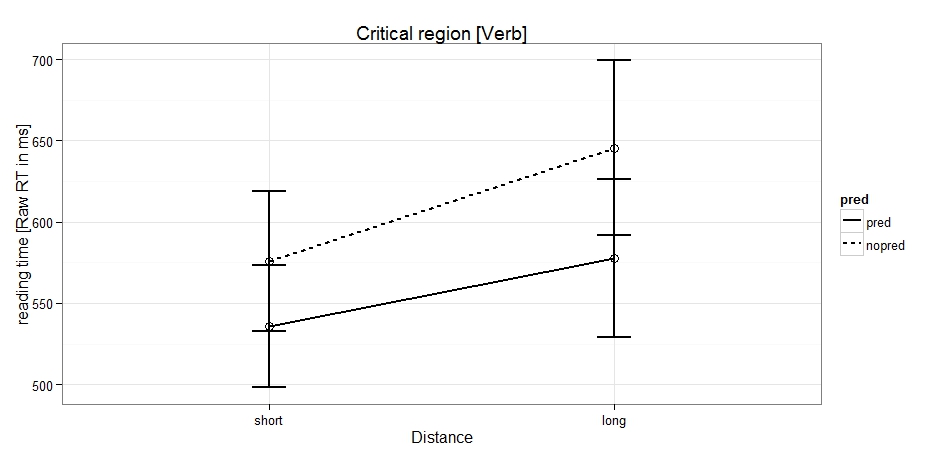

Supplement: Supplementary file 1 [file DataSheet1.zip › SafaviEtAl2016DataCode/SPR/SPR.plots/SPR1.plots/interaction plot - SPR 1.jpeg]

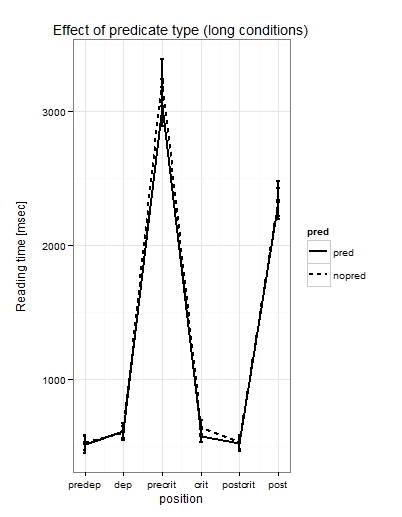

Supplement: Supplementary file 1 [file DataSheet1.zip › SafaviEtAl2016DataCode/SPR/SPR.plots/SPR1.plots/SPR 1 - by region - predicate type - long.jpeg]

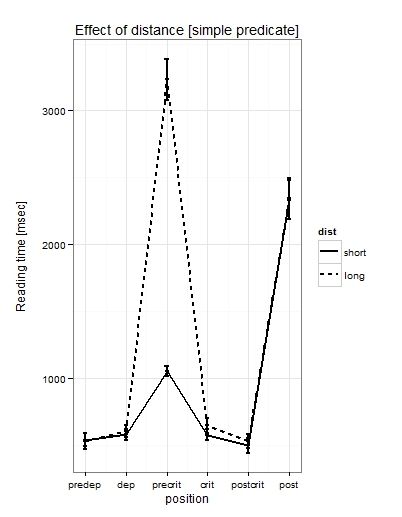

Supplement: Supplementary file 1 [file DataSheet1.zip › SafaviEtAl2016DataCode/SPR/SPR.plots/SPR1.plots/SPR 1 - byregion - distance - SP.jpeg]

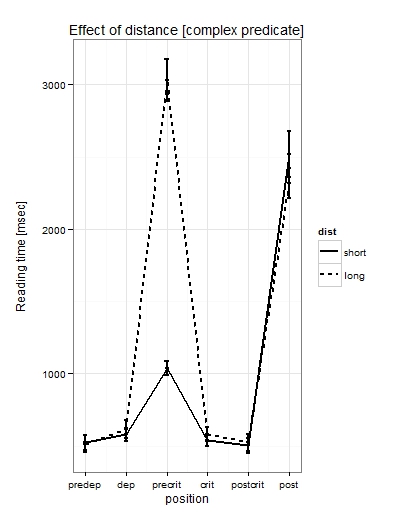

Supplement: Supplementary file 1 [file DataSheet1.zip › SafaviEtAl2016DataCode/SPR/SPR.plots/SPR1.plots/SPR 1 - byregion - distance CP.jpeg]

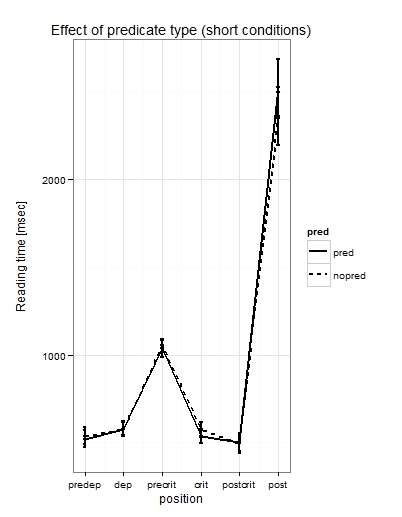

Supplement: Supplementary file 1 [file DataSheet1.zip › SafaviEtAl2016DataCode/SPR/SPR.plots/SPR1.plots/SPR 1 - byregion- predicate type- short.jpeg]

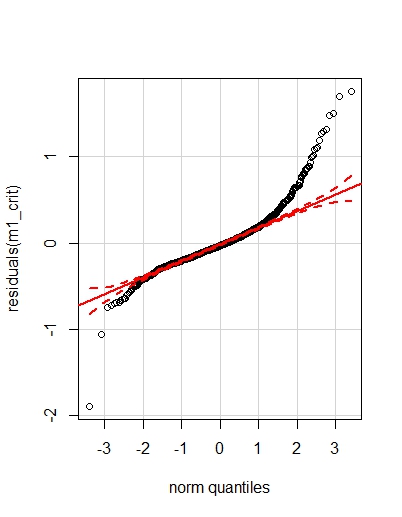

Supplement: Supplementary file 1 [file DataSheet1.zip › SafaviEtAl2016DataCode/SPR/SPR.plots/SPR1.plots/SPR 1 - residuals - postcrit.jpeg]

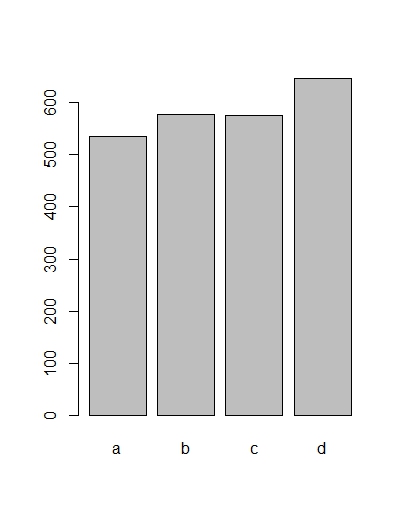

Supplement: Supplementary file 1 [file DataSheet1.zip › SafaviEtAl2016DataCode/SPR/SPR.plots/SPR1.plots/SPR 1 -rt means crit.jpeg]

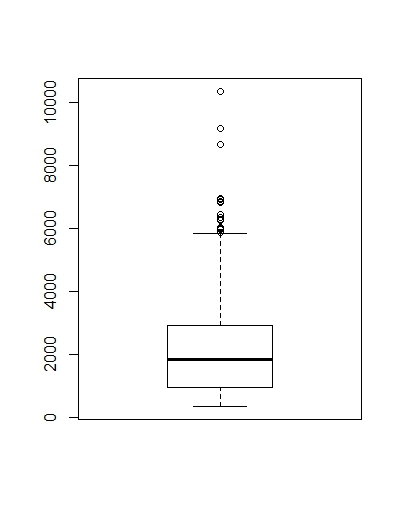

Supplement: Supplementary file 1 [file DataSheet1.zip › SafaviEtAl2016DataCode/SPR/SPR.plots/SPR1.plots/SPR 1 boxplot precrit RT.jpeg]

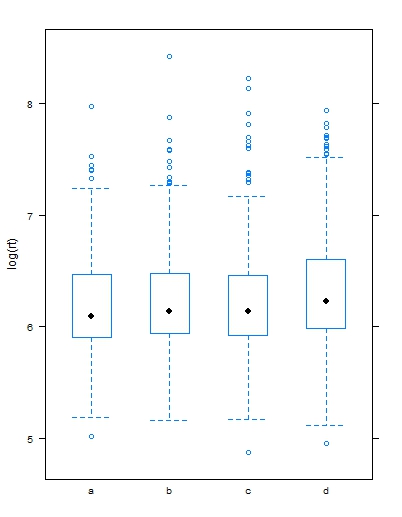

Supplement: Supplementary file 1 [file DataSheet1.zip › SafaviEtAl2016DataCode/SPR/SPR.plots/SPR1.plots/SPR 1 bwplot log rt crit.jpeg]

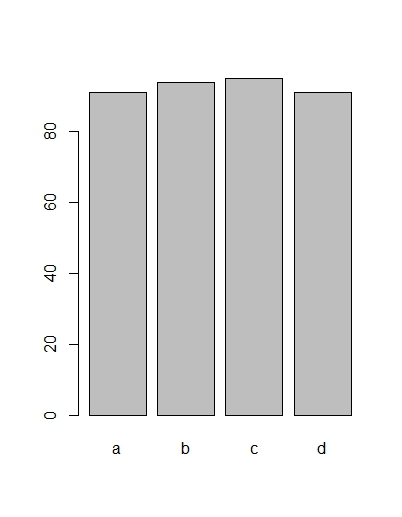

Supplement: Supplementary file 1 [file DataSheet1.zip › SafaviEtAl2016DataCode/SPR/SPR.plots/SPR1.plots/SPR e 1 - resp accuracy.jpeg]

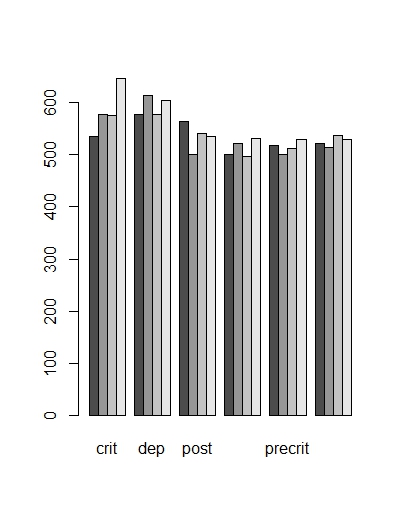

Supplement: Supplementary file 1 [file DataSheet1.zip › SafaviEtAl2016DataCode/SPR/SPR.plots/SPR1.plots/SPR e 1 means.reg.jpeg]

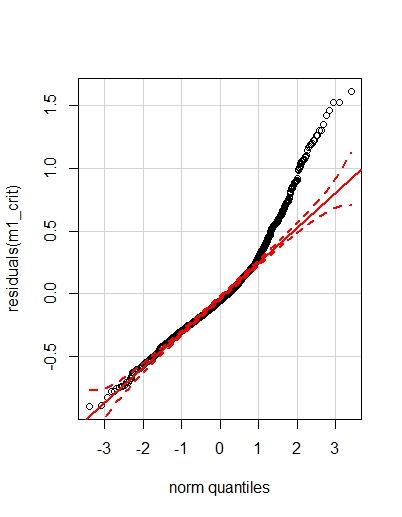

Supplement: Supplementary file 1 [file DataSheet1.zip › SafaviEtAl2016DataCode/SPR/SPR.plots/SPR1.plots/SPR e 1 residuals crit.jpeg]

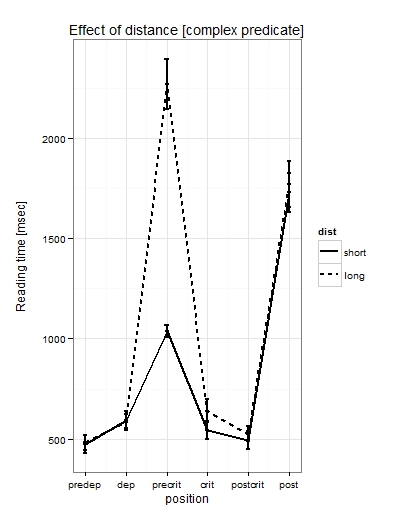

Supplement: Supplementary file 1 [file DataSheet1.zip › SafaviEtAl2016DataCode/SPR/SPR.plots/SPR2.plots/SPR 2 - byregion - distance - CP.jpeg]

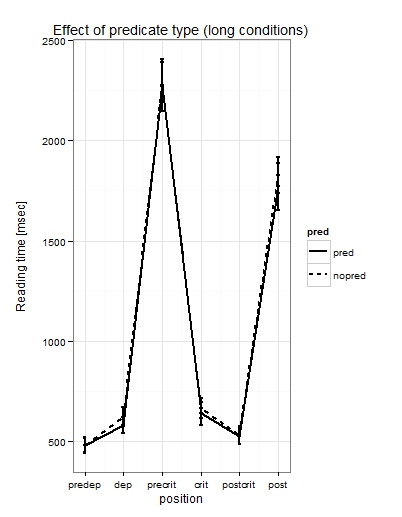

Supplement: Supplementary file 1 [file DataSheet1.zip › SafaviEtAl2016DataCode/SPR/SPR.plots/SPR2.plots/SPR 2 - byregion - predicate type - long.jpeg]

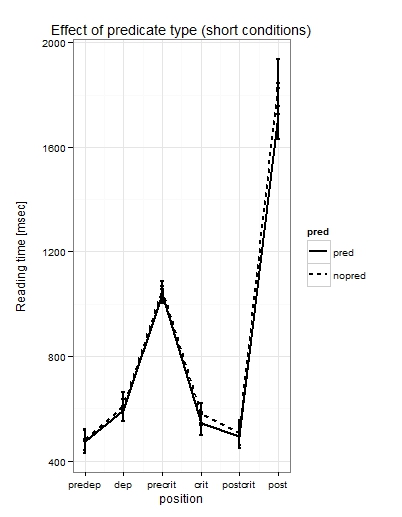

Supplement: Supplementary file 1 [file DataSheet1.zip › SafaviEtAl2016DataCode/SPR/SPR.plots/SPR2.plots/SPR 2 - byregion - predicate type - short.jpeg]

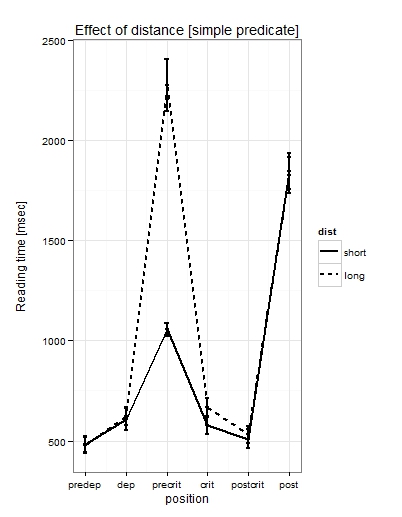

Supplement: Supplementary file 1 [file DataSheet1.zip › SafaviEtAl2016DataCode/SPR/SPR.plots/SPR2.plots/SPR 2 - byregion- distance SP.jpeg]

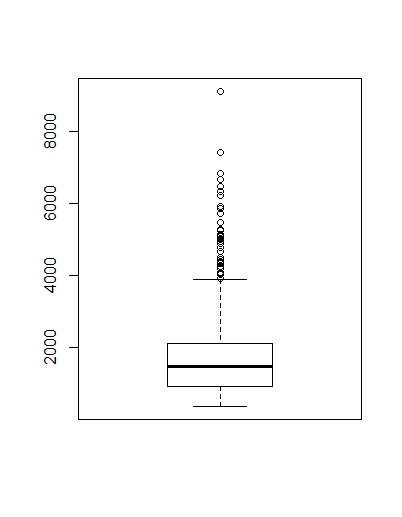

Supplement: Supplementary file 1 [file DataSheet1.zip › SafaviEtAl2016DataCode/SPR/SPR.plots/SPR2.plots/SPR 2 boxplot precrit RT.jpeg]

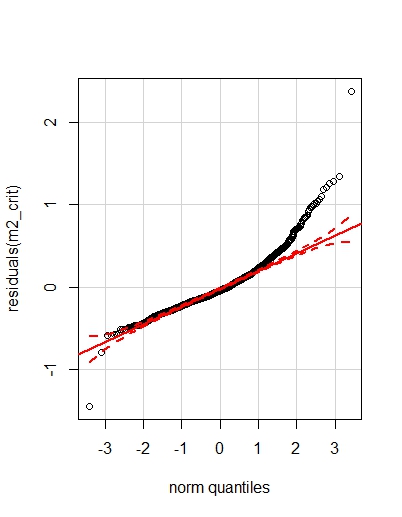

Supplement: Supplementary file 1 [file DataSheet1.zip › SafaviEtAl2016DataCode/SPR/SPR.plots/SPR2.plots/SPR 2 postcrit residuals.jpeg]

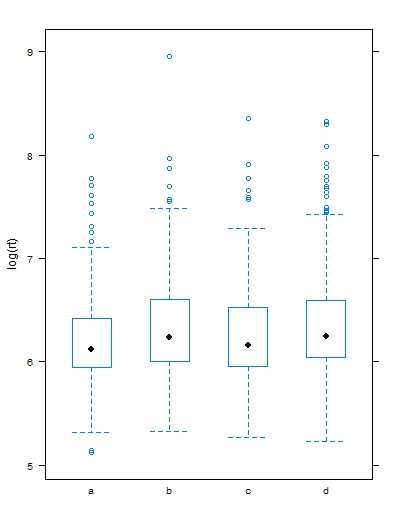

Supplement: Supplementary file 1 [file DataSheet1.zip › SafaviEtAl2016DataCode/SPR/SPR.plots/SPR2.plots/SPR e 2 - bwplot.jpeg]

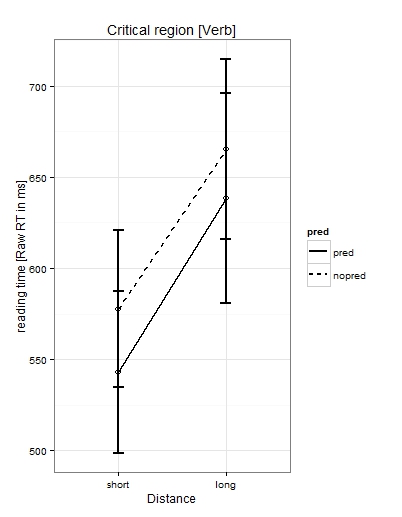

Supplement: Supplementary file 1 [file DataSheet1.zip › SafaviEtAl2016DataCode/SPR/SPR.plots/SPR2.plots/SPR e 2 - interaction plot.jpeg]

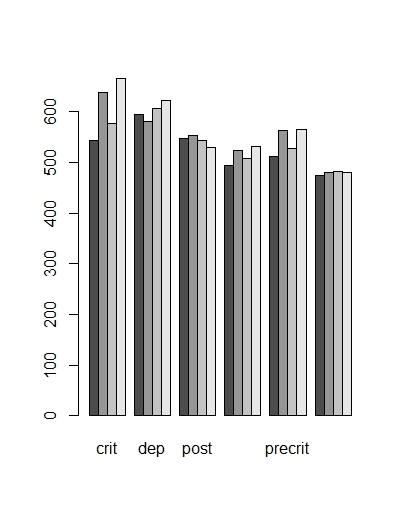

Supplement: Supplementary file 1 [file DataSheet1.zip › SafaviEtAl2016DataCode/SPR/SPR.plots/SPR2.plots/SPR e 2 - means reg.jpeg]

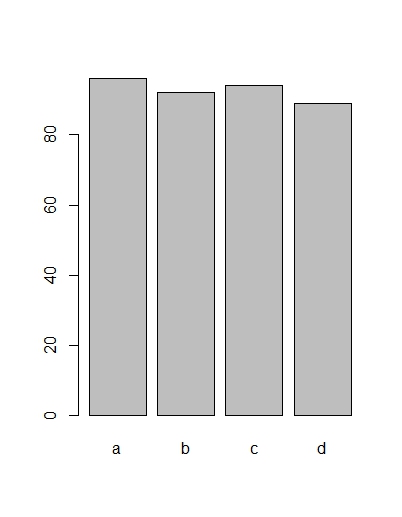

Supplement: Supplementary file 1 [file DataSheet1.zip › SafaviEtAl2016DataCode/SPR/SPR.plots/SPR2.plots/SPR e 2 - response accuracy.jpeg]

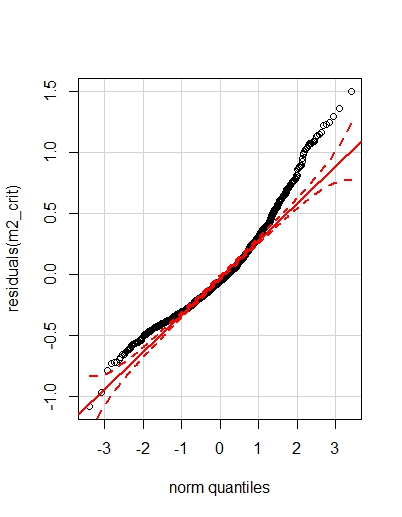

Supplement: Supplementary file 1 [file DataSheet1.zip › SafaviEtAl2016DataCode/SPR/SPR.plots/SPR2.plots/SPR e 2 crit residuals.jpeg]

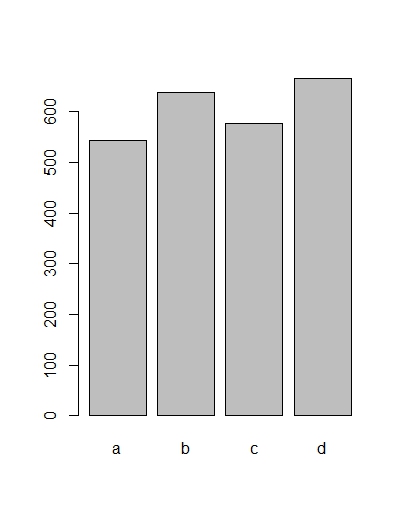

Supplement: Supplementary file 1 [file DataSheet1.zip › SafaviEtAl2016DataCode/SPR/SPR.plots/SPR2.plots/SPR e 2 means.jpeg]
